# General Methodologies Toward *cis*-Fused Quinone Sesquiterpenoids. Enantiospecific Synthesis of the *epi*-Ilimaquinone Core Featuring Sc-Catalyzed Ring Expansion

**DOI:** 10.3390/molecules22071041

**Published:** 2017-06-24

**Authors:** Hilan Z. Kaplan, Victor L. Rendina, Jason S. Kingsbury

**Affiliations:** 1 Eugene F. Merkert Chemistry Center, Boston College, 2609 Beacon Street, Chestnut Hill, MA 02467, USA; hilan.kaplan@gmail.com (H.Z.K.); vrendina@gmail.com (V.L.R.); 2 Ahmanson Science Center, California Lutheran University, 60 West Olsen Rd., Thousand Oaks, CA 91360, USA

**Keywords:** quinone natural products, quaternary carbon synthesis, *cis*-decalin, scandium triflate, catalytic ring expansion, homologation, (trimethylsilyl)diazomethane, hexahydroindanones, 5-*epi*-ilimaquinone, 5-*epi*-isospongiaquinone

## Abstract

A stereocontrolled approach to the *cis*-decalin framework of clerodane diterpenes and biologically active quinone sesquiterpenes is reported. Starting from an inexpensive optically pure tetrahydroindanone, Birch reductive alkylation builds two new contiguous chiral centers—one of which is quaternary and all-carbon-substituted. Also featured is a highly regioselective diazoalkane—carbonyl homologation reaction to prepare the 6,6-bicyclic skeleton. Therein, the utility of Sc(OTf)_3_ as a mild catalyst for formal 1C insertion in complex settings is demonstrated.

## 1. Introduction

**Figure 1:**
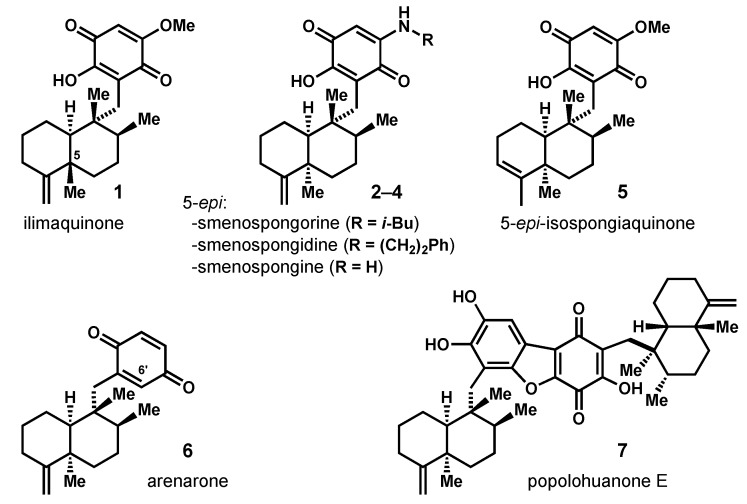
Bioactive *cis*-fused sesquiterpene quinones and their relationship to (−)-ilimaquinone.

Earth’s biodiverse oceans remain a prolific source of small-molecule natural products [1]. The medicinal profile for the sea sponge metabolite ilimaquinone [2] (**1**, Figure 1) includes antiviral, anti-inflammatory, antimicrobial, and antitumor activities [3,4] as well as the ability to protect the cell against ricin and diptheria toxins [5]. Efforts to understand how ilimaquinone causes a reversible breakdown of the Golgi apparatus [6] have been facilitated by total synthesis [7,8,9,10] and point to its influence on cellular methylation [11]. Well over a hundred sesquiterpenes with avarane frameworks fused to electrophilic quinone subunits are known [12], including those that bear an epimeric *cis* ring fusion in the decalin core (Figure 1). It is fascinating that this and other seemingly minor structural changes can translate to pharmacological effects that are unique to each natural product [13].

For instance, 5-*epi*-ilimaquinone and its amine-based variants **2**–**4** are more active than **1** in the production of interleukin-8 (IL-8), a key cytokine in tumor progression and metastasis, but only 5-*epi*-smenospongine (**4**) raises the production of TNF-α in murine leukemia cells [14]. This data, along with the fact that popolohuanone E (**7**) is a potent nanomolar inhibitor of topoisomerase II with efficacy against human non-small-cell lung cancer [15], suggests that a flexible synthesis of the *cis*-fused clerodane core structure would be valuable. The ability to target entire families of bioactive natural products rests upon the availability of inexpensive reagent or catalyst systems that are convenient, reliable, and stereodiscriminating. Herein, we report on a widely applicable synthesis of complex *cis*-decalins by a concise and scalable route featuring catalytic ring expansion methodology.

Previous synthetic studies within the *cis*-fused series have focused on arenarol [16,17,18], since its 6’-hydroxy variant is a biosynthetic precursor of the cytotoxic dimer **7** (Figure 1) [19]. Common strategies for accessing the *cis* ring juncture include bicyclization through intramolecular Hosomi-Sakurai reactions [18,20] and the substrate-controlled hydrogenation of ene-decalin derivatives that are readily prepared by Robinson annulation [16,17]. A comparable excess of syntheses have been recorded for the *trans*-fused class of targets [21,22,23,24,25], including ilimaquinone [7,8,9,10]. These cases also rely on Wieland–Miescher-type starting materials, where Birch reduction–alkylation serves well to install a thermodynamically favored *trans* ring juncture [26,27].

**Scheme 1:**
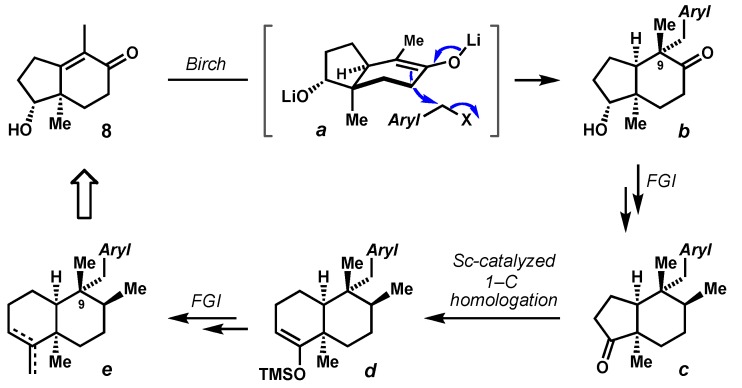
Entry to complex *cis*-decalins based on retrosynthetic simplification to a tetrahydroindanone.

In designing our approach (Scheme 1), two cases [28,29] of dissolving metal reduction with tetrahydroindanone ([4.3.0]-bicyclic) substrates were found showing a kinetic preference [30] for *cis* stereochemistry. However, in neither case was the potential for diastereoselective alkylation of the metal enolate explored. We reasoned that upon hydrogen atom abstraction by the radical anion to give ***a***, face-selective quaternization of the α-carbon would be facilitated by the cup-shaped nature of the intermediate (***a*** → ***b***). After methylenation, stereoselective hydrogenation, and oxidation (***b*** → ***c***), we planned to apply our Sc-catalyzed method [31] for regioselective ring expansion (***c*** → ***d***) with (trimethylsilyl)diazomethane (TMSD). The ring-enlarged enolsilane ***d*** could then diverge to both exocyclic and endocyclic alkenes (→ ***e***, compare 5-*epi*-**1** vs. **5**, Figure 1). Additional end-game manipulations would involve unmasking of the bioactive quinone subunits in each target molecule.

## 2. Results and Discussion

### 2.1. Diastereochemical Control at the C9 Quaternary Carbon by Birch Alkylation

**Scheme 2:**
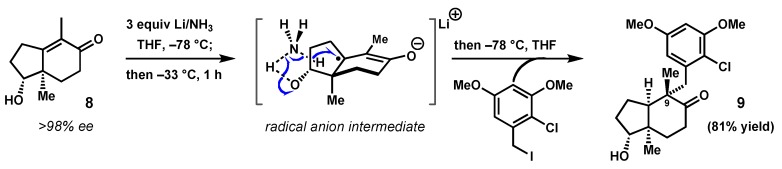
Dissolving metal reduction–alkylation of tetrahydroindanone builds contiguous stereocenters.

Our investigation began with multigram synthesis and enantioenrichment of reduced Hajos-Parrish ketone **8** according to a modified literature protocol. The efficiency of a known [32] d-Phe-catalyzed synthesis of C4-alkylated tetrahydroindandiones has been improved by minimizing solvent and sonicating the reaction mixture [33]. Complete separation of the minor enantiomer (92 → >98% ee) was achieved through a single recrystallization of α carbinol **8**, itself obtained by stereoselective borohydride reduction of the diketone. As shown below in Scheme 2, dissolution of this material in Li-NH_3_/THF at −78 °C, followed by brief warming to −33 °C, re-cooling to −78 °C, and trapping with excess 2-chloro-3,5-dimethoxybenzyl iodide affords hexahydroindanone **9** as a single diastereomer in 81% yield after aqueous ammonium chloride workup and chromatography. The only detectable byproducts stem from protonation or *O*-alkylation of the putative Li enolate. The scope of this convergent reductive C–C coupling process includes both doubly *ortho*-substituted benzylic and allylic bromides or iodides and was communicated previously by our laboratory [34].

Of interest are the perfect levels of relative configurational control imparted by the existing secondary hydroxyl and angular methyl groups. Alcoholic additives such as methanol, *t*-butanol, and even water have long been known [35] to improve efficiency and yield in dissolving metal reductions, presumably by acting as H atom sources for high energy radical anion (or even dianion) intermediates. That the chosen substrate [7] contains an unprotected alcohol as a stoichiometric, built-in proton source may be enabling. For example, an attempt to reproduce the above data with cyclopentyl ethylene ketal (derived from the dione of **8**) or the *t*-butyl dimethylsilyl (TBS) ether of **8** delivers *cis*-fused products but with noticeable reductions (>15–20%) in chemical yield. At this point, we do not rule out the possibility [34] that the more efficient transformations observed for tetrahydroindanol **8** benefit from intramolecular H atom abstraction within a radical anion intermediate formed by a kinetically favored, single-electron reduction of the enone. Molecular models do not convincingly demonstrate that the cyclopentyl hydroxyl is close enough in proximity to the β carbon to permit internal delivery, but with the potential for participation by a solvent molecule (NH_3_, Scheme 2) the suggested process is likely more facile than intermolecular alternatives. Once a *cis* ring fusion is established, stereochemical induction during formation of the congested C9 quaternary carbon is likely the result of electrophilic approach being restricted to the convex side of the intermediate.

### 2.2. Further Elaboration of the Stereotriad Gives a Lower Homologue of the epi-Ilimaquinone Core

**Scheme 3:**
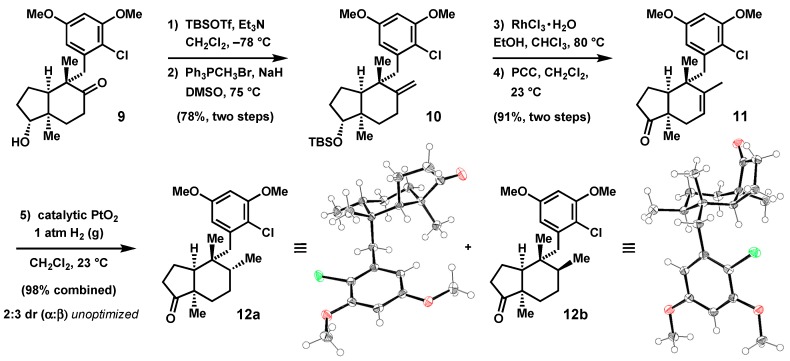
Forward synthesis before ring expansion, including Oak Ridge Thermal Ellipsoid Plot (ORTEP) plots for hexahydroindanones **12**.

Gratifyingly, further transformation of the hexahydroindanone adduct **9** described above is successful despite steric crowding imposed by the new quaternary center. These studies commenced with a standard TBS protection of the free cyclopentanol (step 1, Scheme 3). Unprotected **9** does furnish an ene-carbinol under the forcing and precedented [21] Wittig conditions shown in step 2 (methyltriphenylphosphonium bromide, dimsyl sodium, 75 °C), but it is the exclusive result of a formal 1,5-hydride shift and subsequent cyclopentanone methylenation [34]. Avoiding Na alkoxide formation prevents this transannular, acyloin-like rearrangement [36] and leads to the desired exocyclic methylene cyclohexane **10** in good yield. Unfortunately, the features that led to high stereoselectivity in the convergent Birch alkylation did not translate to alkene saturation. All efforts to hydrogenate **10** (or its free alcohol) to establish the C8 β methyl stereocenter were met with low conversion or predominant formation of the unnatural diastereomer.

Judging that the adjacent quaternary carbon was discouraging metal hydride approach overall yet allowing it *syn* to the smaller (methyl) group, migration of the alkene into the bicycle and farther from the site of steric congestion was pursued. Rhodium(III)-mediated isomerization [23] with concomitant silyl ether cleavage, followed by pyridinium chlorochromate (PCC) oxidation, provided cyclopentanone **11** in 91% yield over two steps (Scheme 3). A range of heterogeneous hydrogenation reactions were then tested for this alternative substrate. A favorable outcome is observed with Adams’ catalyst at ambient temperature and pressure, delivering epimeric hexahydroindanones **12a** and **b** in near quantitative yield and an unoptimized 2:3 dr slightly favoring the desired β methyl isomer. The compounds proved chromatographically separable and crystalline, allowing for rigorous structure proof by X-ray diffraction (Scheme 3). At this stage, our attention turned to the key catalytic ring expansion with two advanced substrates in hand (**11** and **12b**). Before embarking on this goal, we sought to establish a benchmark for reactivity and regioselectivity using a suitable model system.

### 2.3. A Steroidal Model System for Catalysis of α-Quaternary Cyclopentanone Ring Enlargements

The accepted order of reactivity for ring expansion of cycloalkanones with diazoalkanes is cyclobutanone ≈ cyclohexanone > cycloheptanone > cyclopentanone on the basis of both empirical findings and literature precedent [37]. A previous report from our laboratory [31] on Sc-catalyzed homologation focused on α-quaternary cyclobutanones, whose reactions benefit from 4C ring strain. We thus found ourselves at the opposite end of this spectrum of reactivity, seeking to ring expand very hindered, neopentylic cyclic ketones lacking much angle strain. In order to ensure that our former reaction conditions would be effective in the case of more reluctant substrates, we carried out initial experimental optimization with commercially available estrone 3-methyl ether (**13**).

**Scheme 4:**
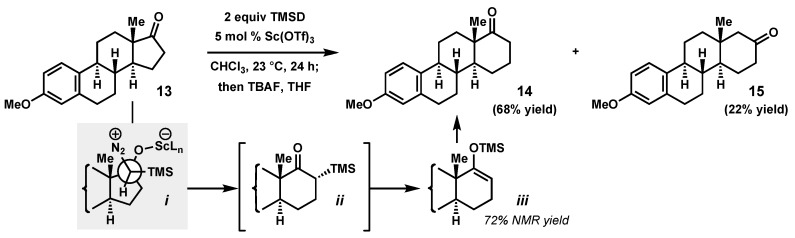
Catalytic regioselective homologation of an estrone by formal (trimethylsilyl)methine insertion.

As shown below in Scheme 4, exposure of **13** to 2 equivalents of TMSD and 5 mol % Sc(OTf)_3_ at 23 °C in chloroform for 24 h gave full conversion of starting material and a 72% NMR yield of the expected cyclohexenyl silyl ether ***iii***. Subsequent workup of the reaction mixture with tetrabutylammonium fluoride (TBAF) and purification by silica gel chromatography afforded the major regioisomer **14** in an acceptable 68% yield along with 22% of **15**. The modest (~3:1) level of regiochemical control in this reaction can be explained by a preferred approach of the diazoalkane nucleophile. Carbonyl 1,2-addition with the bulky silicon group oriented away from the quaternary α carbon situates the methylene substituent anti-coplanar to the dinitrogen leaving group (***i***, Scheme 4). Subsequent 1,2-migration generates a stereodefined α-trimethylsilyl cyclohexanone ***ii***. However, our previous results [31] confirm that the regenerated Sc(OTf)_3_ goes on to catalyze secondary 1,3-Brook rearrangement [38], affording ***iii*** as the major product prior to desilylation. Noteworthy is the fact that no unwanted hydrolysis of enol silane ***iii*** occurs under the reaction conditions: this prevents overhomologation of the more reactive products. The simple regiochemical model based on a need to minimize non-bonding interactions during diazoalkyl 1,2-addition is in accord with other literature reports [39].

Over the course of experimentation that led to the above conditions for effective preparation of homoestrone **14**, a number of important observations were made that we wish to summarize: (1) Consumption of **13** is prohibitively slow at temperatures of 0 °C or below, and solvent screening pointed to toluene, CH_2_Cl_2_, and CHCl_3_ as media that promote smooth conversion and high levels of regioselectivity. Coordinating solvents, such as Et_2_O and THF, suppress catalyst efficiency. The halogenated solvents also improve homogeneity, and thus CHCl_3_ was chosen due to its lower volatility and a preference to monitor reaction progress by ^1^H-NMR spectroscopy; (2) Other Sc(III) salts and other lanthanide triflates were examined, but Sc(OTf)_3_ remained the optimal catalyst. A regioisomeric ratio as high as 55:1 was recorded for Yb(OTf)_3_ with a monocyclic α-quaternary substrate, suggesting that the larger Lewis acid leads to a more selective addition of the diazoalkane. Unfortunately, conversion was not as high relative to Sc(OTf)_3_, and attempts to use the stronger Yb(NTf_2_)_3_ resulted in steady decomposition of the nucleophile; (3) Finally, trials performed with the commercial hydrate of Sc(OTf)_3_ or unpurified reagent and solvent gave irreproducible results. Trace amounts of adventitious water were previously found to have a profound impact on both the rate and enantioselectivity of asymmetric ring expansion reactions with chiral Sc catalysts [40]. Control experiments have shown that although TMSD itself is stable to both water and Sc(OTf)_3_ separately, the combination of all three leads to rapid destruction of TMSD, with a measured t_1/2_ of only 20 min. Sc(OTf)_3_ is a water-tolerant trication and is prepared from aqueous TfOH [41], but hydration of its coordination sphere creates an equilibrium generating the free Brønsted acid. This, in turn, allows net OH insertion by TMSD, giving TMSCH_2_OH (or CH_3_OTMS after 1,2-Brook rearrangement). All variables considered, the complex cyclopentanone homologations reported in the next section were set up in a glovebox with only rigorously dried Sc(OTf)_3_, TMSD, and CHCl_3_.

### 2.4. Advancement of Synthetic Bicyclopentanones to the epi-Ilimaquinone Core by Ring Expansion

**Scheme 5:**
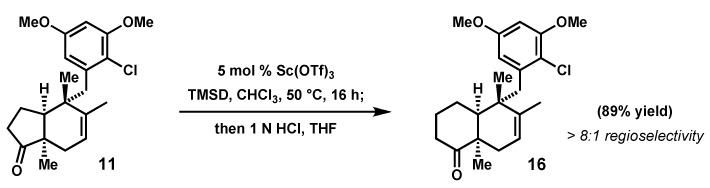
Successful ring expansion of synthetic bicyclopentanone **11** by gentle warming to 50 °C.

We were pleased to find that the conditions optimized for steroidal model **13** tracked quite well to our first synthetic cyclopentanone **11** with very little modification (Scheme 5). Exposure of **11** to 10 mol % Sc(OTf)_3_ and 1.5 equivalents of TMSD in CHCl_3_ showed only 33% conversion after 18 h at 23 °C in a J Young NMR tube. However, by simply heating the solution to 50 °C, >98% conversion was reached in 18 additional hours. Upon dilute acid hydrolysis, the regioselectivity by ^1^H-NMR analysis was approximately 6:1 favoring the desired regioisomer **16**. By dropping the catalyst loading to 5 mol %, increasing concentration, and heating the reaction from the outset, the desired *cis*-decalone (**16**) was isolated in an 89% purified yield after just 16 h. Integration of the mixture before protodesilylation in the latter case showed 8:1 regioselectivity (Scheme 5).

**Scheme 6:**
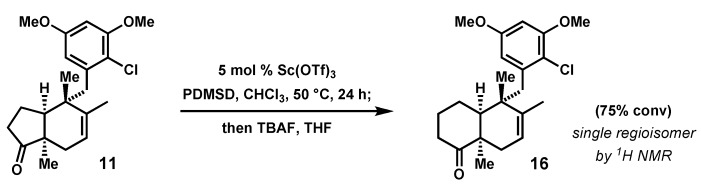
Complete regiochemical control but lower efficiency with (phenyldimethylsilyl)diazomethane.

Notwithstanding this successful opening result, we were curious to know if the regioisomeric ratio (8:1) could be further enhanced by recourse to a more hindered (trialkylsilyl)diazomethane. (Phenyldimethylsilyl)diazomethane (PDMSD) was prepared in our prior study [31] by adapting the known protocol for TMSD [42] in order to allow Fleming–Tamao oxidation [43] of β-keto silane products (see ***ii***, Scheme 4) that are stable to Brook rearrangement if Sc(hfac)_3_ is used as catalyst. As shown in Scheme 6, heating of the same ketone with 5 mol % Sc(OTf)_3_ and the bulkier PDMSD resulted in steady ring expansion, and only a single regioisomer was detectable after 24 h by ^1^H-NMR spectroscopy. Regrettably, the conversion had only reached 75% during this time period. As expected, the larger silyl substituent ensures a complete preference for the addition mode that places it opposite the quaternary center (Scheme 4). This can be useful in cases where regioisomers are not otherwise separable by column chromatography. However, we remained content with the utilization of TMSD as a commercial 1C source that clearly promotes the most efficient reactions.

Surprisingly, when the saturated β-methyl bicyclopentanone **12b** was subjected to identical homologation conditions that had cleanly transformed **11**, we were disappointed to see complete lack of reactivity. Prolonged heating of the reaction mixture at 50 °C did nothing to drive a ring expansion, and the starting material was returned unchanged. In a more forceful experiment with six equivalents of TMSD, heating to 70 °C led to extensive decomposition of both the diazoalkane and substrate. Not even trace amounts of the characteristic cyclohexyl enol silane could be found. Eventually, an experiment performed on a mixture of the α- and β-methyl cyclopentanones (**12a**,**b**) with two equivalents of TMSD and 5 mol % Sc(OTf)_3_ at 50 °C overnight provided insight. Complete conversion of the α epimer was visible by ^1^H-NMR, but the β epimer remained totally untouched. This control indicated that our reaction was working properly, yet something particular about the β isomer **12b** was preventing diazoalkane–carbonyl homologation from occurring.

Close scrutiny of the X-ray structure of **12b** (Scheme 3) reveals a sound rationale for why this substrate fails to undergo catalytic homologation even under strongly forcing conditions. Access to the π* orbital of the carbonyl is completely blocked by methyl groups on both sides of the bicycle. The α face is effectively shielded by the adjacent methyl, and the β face is even more encumbered by the axial methyl group at the C9 quaternary center. In contrast, the solid-state structure for **12a** represents the ‘chair-flip’ conformer, with each quaternary methyl group falling nearly in plane with the cyclopentanone ring and orthogonal to the π* orbital. Though each representation of the solid state may not accurately represent conformations accessible in solution—especially at higher temperatures—these structural features shed light on why **12a** readily homologates, whereas **12b** is completely inert.

**Scheme 7:**
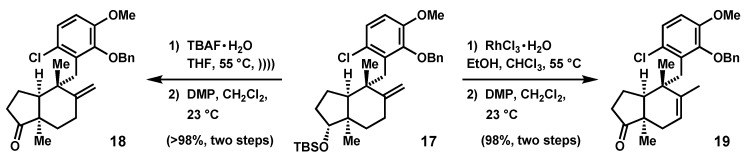
Divergent entry to new ring expansion substrates with orthogonal phenolic protecting groups.

With a success rate for ring expansion involving just two out of three substrates, we sought to devise a second-generation strategy that would address some of the deficiencies in our approach to the natural products. Foremost among these were the low degree of stereocontrol in hydrogenation with PtO_2_ and our inability to transform **12b** to a fully saturated avarane core with the correct C8 configuration for the tertiary methyl group. Specifically, we wanted to incorporate functionality into the Birch trapping agent that could be selectively unmasked and provide a means to direct a homogeneous hydrogenation catalyst to the α face of either unsaturated bicyclopentanone (see **10** or **11**, Scheme 3). Thus, 2-benzyloxy-6-chloro-3-methoxybenzyl iodide [34] was secured, containing an orthogonally protected phenol that we planned to later test as a directing group and functional handle for quinone oxidation. Under precisely the same conditions of Scheme 2, Birch alkylation with the new electrophile gave the desired keto-alcohol in 79% yield. Analogous TBS protection and Wittig olefination then delivered compound **17** (see Scheme 7) in 85% yield over two steps.

At this point in the original route, we had isomerized the exocyclic alkene to help facilitate a modestly selective hydrogenation over Adams’ catalyst. By diverging our material and bringing forward both the 1,1-disubstituted and trisubstituted olefins, we would have two more substrates to showcase the generality of the Sc-catalyzed ring enlargement. As illustrated above in Scheme 7, direct deprotection of **17** with TBAF followed by Dess–Martin oxidation furnished the exocyclic ene-bicyclopentanone **18** in quantitative yield. In parallel, rhodium-mediated isomerization and desilylation of **17** followed again by periodinane oxidation afforded its endocyclic constitutional isomer **19** in 98% yield over two steps.

We then returned to an exploration of catalytic ring expansion with two additional complex cyclopentanone substrates in hand (**18** and **19**). Both structures readily underwent 1C homologation with mild warming under the optimum conditions, reaching full conversion in less than 24 h. Still, as structural isomers likely having innate conformational preferences, it was not surprising to find measurable differences in efficiency and regiochemistry.

**Scheme 8:**
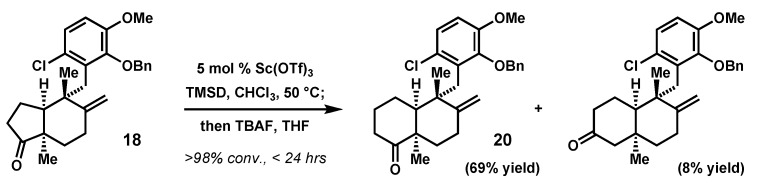
Homologating the 1,1-disubstituted exocyclic olefin **18** gives a diminished selectivity and yield.

**Scheme 9:**
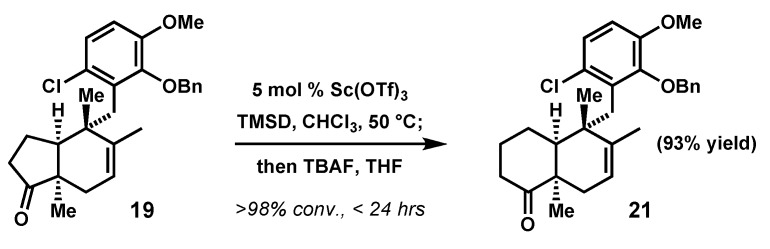
Excellent yield and >10:1 regiochemical control for ring expansion of **19**.

Shown below (Scheme 8), the exocyclic ene-cyclopentanone **18** transformed with a 7:1 ratio of cyclic enol silanes detectable by NMR prior to hydrolysis with fluoride. Upon workup and column chromatography, 69% of the major ene-decalone **20** was isolated along with 8% of its regioisomer. By contrast, the isomerized cyclopentanone **19** bearing an additional *sp*^2^ hybrid carbon in its core afforded a much higher 93% isolated yield of the target homologated product **21**, as shown below in Scheme 9. The latter outcome tracks well with the result obtained earlier for the trisubstituted alkene **11**, differing only in the substitution and pattern on the arene that is removed from the site of reaction (89% yield, >8:1 rr, Scheme 5). Overall, this direct comparison of ring expansion data underscores how seemingly subtle changes in molecular structure and solution-phase conformations have a fairly striking effect on the efficiency with which the ketone acceptor, the diazoalkane reagent, and perhaps even the Lewis acidic catalyst approach one another and engage in reaction.

**Figure 2:**
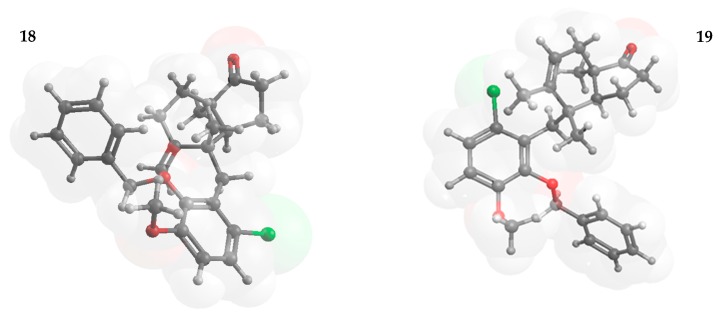
Computational models for complex cyclopentanones **18** and **19** suggest distinct chair conformers.

The above conclusion begs a question of whether everyday practitioners of organic synthesis could gain some predictive power over how different kinds of ring systems and their unique shapes will behave in this classic and strategically useful reaction. Modeling of complex cyclopentanones **18** and **19** in silico confirms that the positioning of the olefin greatly impacts the preferred chair conformation. Optimized geometries were calculated with Guassian ’09–B3LYP 3-21G/Avogadro 1.03. The exocyclic alkene bicyclopentanone **18** (left, Figure 2) prefers a twist-boat conformation in which both quaternary methyl groups are pseudoaxial and more effectively block Bürgi–Dunitz approach to the carbonyl. Though not as prohibitive a situation, this picture resembles the X-ray conformation that was characteristic of the totally unreactive saturated hexahydroindanone **12b**, and to an extent explains the less selective and efficient result recorded in Scheme 8. On the other hand, endocyclic alkene bicyclopentanone **19** adopts a half-chair structure with the arene subunit and angular methyl group in a distorted 1,3-diaxial orientation (right, Figure 2). Note that in each case, the molecules are using rotational freedom as a way to avoid a severe *syn* pentane interaction that is incurred for any perfect chair conformer given the 1,3-relationship of the fully substituted quaternary carbons. The predicted conformation for **19** reveals a more favorable and less congested environment about the ketone that, in turn, positively affects the outcome of methylene insertion.

## 3. Materials and Methods

### 3.1. General Remarks

All reactions were carried out in flame-dried glassware under an atmosphere of argon in dry, degassed solvents using standard Schlenk and vacuum-line techniques. Particularly air-sensitive manipulations were performed in an MBraun Unilab nitrogen atmosphere glove box (MBraun USA, Stratham, NH, USA). Commercial reagents were used as received unless otherwise noted. Flash column chromatography was driven by compressed air and performed with ZEOPrep 60 Eco 40–63 μm silica gel (AICMA, Framingham, MA, USA). Analytical thin-layer chromatography (TLC) was conducted with 0.25 mm silica gel 60 F254 plates purchased from EMD Chemicals (Gibbstown, NJ, USA). Sc(OTf)_3_ (99%, Sigma-Aldrich, St. Louis, MO, USA) was finely powdered, dried at 200 °C over P_2_O_5_ for 24 h under high vacuum (0.1 mm of Hg), and recovered in the glovebox. (Trimethylsilyl)diazomethane (TMSD) and (phenyldimethylsilyl)diazomethane (PDMSD) were prepared according to a known procedure [41] and stored over freshly activated 3 Å molecular sieves at −40 °C in glovebox freezer. Note: TMSD is both non-explosive and non-mutagenic; however, it is extremely toxic and should be handled with suitable precautions. A representative homologation protocol and characterization for compounds **14** and **15** are provided. Experimental procedures for structures **9**, **10**, and the ketone precursor to **17** are omitted since they have been reported elsewhere [34]. Additional details for synthesis and identification of total synthesis intermediates **8**, **11**, **12a**, **12b**, **16**, **17**, **18**, **19**, **20** and **21** were compiled from a previous source [44] and have been included as Supplementary Material.

FT-IR spectra were recorded on a Bruker Alpha-p spectrometer (Bruker Optics Inc., Billerica, MA, USA). Bands are reported as strong (s), medium (m), weak (w), broad strong (bs), broad medium (bm), and broad weak (bw). Optical rotation data was recorded on a Rudolph research Autopol IV automatic polarimeter (Rudolph Research Analytical, Hackettstown, NJ, USA) and is given as the average of five readings. Melting points were recorded on a Digimelt MPA160 SRS (Stanford Research Systems Inc., Sunnyvale, CA, USA) and are uncorrected. Sonication was performed with a Misonix Sonicator 3000 (Misonix Inc., Farmingdale, NY, USA) linked to a Laude external circulator for temperature control. ^1^H-NMR spectra were collected on Varian VNMRS (500 MHz) or INOVA (500 MHz) (Agilent Technologies, Santa Clara, CA, USA) spectrometers. Chemical shifts are reported in ppm from tetramethylsilane with the solvent resonance as the internal standard (CHCl_3_: δ 7.26). Data are reported as follows: chemical shift, multiplicities (s = singlet, d = doublet, dd = doublet of doublets, ddd = doublet of doublet of doublets, dddd = doublet of doublet of doublet of doublets, t = triplet, m = multiplet), coupling constants (Hz), and integration. ^13^C-NMR spectra were recorded on Varian VNMRS (125 MHz) or INOVA (125 MHz) spectrometers with complete proton decoupling. Chemical shifts are reported in ppm from tetramethylsilane with the solvent as the internal reference (CDCl_3_: δ 77.16). High-resolution mass spectra were collected at the Boston College Mass Spectrometry Facility (Merkert Chemistry Center, Chestnut Hill, MA, USA). Supercritical fluid chromatography (SFC) data were obtained on a Berger Instruments system (Waters Corp., Milford, MA, USA) using a Daicel CHIRALPAK AS-H column (φ 4.6 mm, 25 cm length).

### 3.2. Representative Procedure for Regioselective Sc-Catalyzed Ring Expansion with Estrone Model

*Homologous estrone 3-methyl ether—Major* (**14**). In a drybox, Sc(OTf)_3_ (3.7 mg, 0.0075 mmol, 0.05 equiv.) was weighed directly into a 1.5 mL vial equipped with a magnetic stirbar. A solution of estrone 3-methyl ether (**13**, 42.6 mg, 0.15 mmol, 1.0 equiv.) in CDCl_3_ (0.53 mL) was transferred directly to the solid Sc(OTf)_3_. The resulting cloudy gray suspension was stirred for 15 min, at which point TMSD (121 μL, 0.30 mmol, 2.0 equiv., 2.47 M in hexanes) was introduced dropwise. The entire reaction mixture (including any residual solids) was transferred with a glass pipette to a J. Young NMR tube, and the vial was rinsed with an additional 0.2 mL of CDCl_3_. The reaction tube was removed from the drybox, connected to a nitrogen manifold, and allowed to stand 24 h at 23 °C. 1,3,5-trimethoxybenzene (11.0 mg, 0.65 mmol, 4.3 equiv.) was added as an internal standard, and ^1^H-NMR integrals indicated a 72% yield of the major cyclic enol silane. The reaction mixture was poured into H_2_O (5 mL), and the product was extracted with CH_2_Cl_2_ (3 × 10 mL). The combined organic layer was dried over Na_2_SO_4_, filtered, and concentrated. The unpurified residue was then dissolved in 1 mL of THF, TBAF·xH_2_O (168 mg, 0.60 mmol, 4.0 equiv.) was added as a solid, and the reaction mixture was stirred for 30 min at 23 °C. The reaction mixture was then poured into H_2_O (5 mL) and the product was extracted with Et_2_O (3 × 5 mL). The combined organic layer was then passed through a short plug of silica gel rinsed with ethyl acetate (10 mL) and concentrated. Purification by column chromatography over silica gel (15% ethyl acetate in hexanes) delivered the desired homologated estrone derivative **14** as a white solid (30.4 mg, 68%), m.p. = 136–138 °C.

*R*_f_ = 0.30 (15% Ethyl acetate in hexanes); ^1^H-NMR (CDCl_3_, 500 MHz) δ 7.22 (dd, *J* = 8.8, 0.5 Hz, 1H), 6.72 (dd, *J* = 8.9, 2.9 Hz, 1H), 6.63 (d, 2.9 Hz, 1H), 3.78 (s, 3H), 2.88–2.83 (m, 2H), 2.67 (ddd, *J* = 14.2, 14.2, 6.8 Hz, 1H), 2.38 (dddd, 11.5, 4.2, 4.2, 4.2 Hz, 1H), 2.28–2.21 (m, 2H), 2.16–2.05 (m, 2H), 1.99–1.93 (m, 1H), 1.89 (ddd, *J* = 13.9, 3.4, 3.4 Hz, 1H), 1.73 (ddd, *J* = 13.7, 13.7, 3.9 Hz, 1H), 1.69–1.58 (m, 1H), 1.55–1.39 (m, 4H), 1.34–1.25 (m, 1H), 1.13 (s, 3H); ^13^C-NMR (CDCl_3_, 125 MHz) δ 216.45, 157.69, 137.76, 132.60, 126.48, 113.59, 111.77, 55.33, 50.44, 48.54, 43.17, 38.99, 37.32, 32.66, 30.24, 26.78, 26.07, 26.03, 23.08, 17.02; IR (neat) 2930 (bs), 2863 (bm), 1703 (s), 1610 (w), 1502 (m), 1429 (bm), 1254 (m), 1237 (m), 1040 (w) cm^−1^; HRMS (ESI+) Calcd. for C_20_H_27_O_2_ [M + H]^+^: 299.2011; Found 299.1999.

*Homologous estrone 3-methyl ether–minor* (**15**). Isolated as a minor regioisomer in the above reaction of compound **14**. Purification by column chromatography (15% ethyl acetate in hexanes) afforded the minor regioisomer **15** as a white solid (9.9 mg, 22%), m.p. = 176–180 °C.

*R*_f_ = 0.17 (15% ethyl acetate in hexanes); ^1^H-NMR (CDCl_3_, 500 MHz) δ 7.22 (d *J* = 8.3 Hz, 1H), 6.73 (dd, *J* = 8.8, 2.9 Hz, 1H), 6.64 (d, *J* = 2.9 Hz, 1H), 3.78 (s, 3H), 2.89–2.83 (m, 2H), 2.47–2.21 (m, 5H), 2.23 (d, *J* = 13.7 Hz, 1H), 2.16–2.09 (m, 1H), 2.14 (d, *J* = 13.4, 2.4 Hz, 1H), 1.67–1.42 (m, 5H), 1.41–1.24 (m, 2H), 0.83 (s, 3H); ^13^C-NMR (CDCl_3_, 125 MHz) δ 211.83, 157.74, 137.95, 132.58, 126.45, 113.64, 111.84, 56.93, 55.38, 48.12, 43.72, 41.38, 41.33, 39.66, 38.38, 30.20, 26.76, 26.50, 25.72, 17.88; IR (neat) 2922 (bs), 2861 (bm), 1709 (s), 1612 (w), 1501 (m), 1256 (s), 1038 (m), 810 (w), 79 (w) cm^−1^; HRMS (ESI+) Calcd. for C_20_H_27_O_2_ [M + H]^+^: 299.2011; Found 299.2015.

## 4. Conclusions

In summary, we paired a known [34] stereoselective Birch reduction–alkylation of [4.3.0]-bicyclic enones with catalytic diazoalkane–carbonyl homologation [45] as methods applicable to the synthesis of a wide range of bioactive quinone sesquiterpenes. Our data constitutes the first examples of Sc-catalyzed 1C homologation with α-quaternary cyclopentanones. In model systems, excellent levels of regioselectivity can be obtained by either using Yb(OTf)_3_ as the catalyst or by employing the more sterically demanding diazoalkane PDMSD (up to >50:1 rr). Rigorous control over environmental variables and reagent purity allows our procedures to be carried out reliably on scale. When extending the catalytic method to more complex substrates, high yields and good levels of regiochemical control were observed (69–93% yield, >8:1 rr).

Methylene insertion is a very common synthetic objective that continues to be carried out with diazomethane in protic solvents [46] or with TMSD in the presence of stoichiometric amounts of BF_3_·Et_2_O [47] and other Al-based promoters [48]. Compared to prior examples in the literature, the new reactions catalyzed by low loadings of Sc(OTf)_3_ are among the highest yielding and most selective [31,45]. Also worthy of note is the stability of TMSD in the presence of catalyst at high temperature and the 1,3-Brook access to enol silane products, which eliminates the possibility for overhomologation. The inability of our most advanced intermediate **12b** to react certainly implies that unforeseen conformational effects may continue to complicate mainstream use of this reaction in total synthesis. Nonetheless, we hope that our findings encourage other synthetic chemists to test the newly developed catalytic conditions in other target-based applications in the future.

## Supplementary Materials

Supplementary materials are available online. Additional experimental details for **8**, **11**, **12a**, **12b**, **16**, **17**, **18**, **19**, **20** and **21**, including copies of ^1^H- and ^13^C-NMR spectra for all new compounds.
