# Supplementary material for: General Methodologies Toward cis-Fused Quinone Sesquiterpenoids. Enantiospecific Synthesis of the epi-Ilimaquinone Core Featuring Sc-Catalyzed Ring Expansion
Source: Molecules. 2017 Jun 24;22(7):1041. doi: 10.3390/molecules22071041 (PMC6152358; doi:10.3390/molecules22071041)

General Methodologies Toward *cis*-Fused Quinone Sesquiterpenoids. Enantiospecific Synthesis of the *epi*-Ilimaquinone Core Featuring Sc-Catalyzed Ring Expansion

Hilan Z. Kaplan, Victor L. Rendina, and Jason S. Kingsbury*

Supplementary Materials

Contact information, corresponding author:

Jason S. Kingsbury, Ph.D.
Assistant Professor of Chemistry
Ahmanson Center, California Lutheran University
60 West Olsen Road # 3700
Thousand Oaks, CA 91360

Phone: (805) 493-3026

Email: jkingsbu@callutheran.edu

Table of Contents **Page**

General Procedures, Materials, and Instrumentation ………………………………………….... S2-S3
Detailed Procedures and Characterization Data ……………………………………………….. S4-S15
Copies of ^{1}H and ^{13}C NMR Spectra ……………………………………………………………. S16-S47

General Procedures

Unless stated otherwise, all reactions were carried out in flame-dried glassware under an atmosphere of argon passed through a tower of Drierite in dry and degassed solvents with standard Schlenk or vacuum-line techniques. Particularly air-sensitive manipulations were performed in an MBraun Unilab nitrogen atmosphere glovebox. Column chromatography was driven by compressed air and performed with ZEOPrep 60 Eco 40-63 μm silica gel. Analytical thin-layer chromatography (TLC) was performed with 0.25 mm silica gel 60 F254 plates from EMD Chemicals. TLC plates were visualized under UV light or by treatment with ceric ammonium molybdate, potassium permanganate, and *para*-anisaldehyde stains.

Materials

Tetrahydrofuran (THF), dichloromethane (CH_2Cl_2), diethyl ether (Et_2O), acetonitrile (CH_3CN), and *N*,*N*-dimethylformamide (DMF) were dispensed under UHP argon from a Glass Contour solvent purification system manufactured by SG Waters, LLC (Nashua, NH). Dimethyl sulfoxide (DMSO), methanol, *tert*-butyldimethylsilyl trifluoromethanesulfonate (TBSOTf), *tert*-butyldimethylsilyl chloride (TBSCl), triethylamine (Et_3N), imidazole, D-phenylalanine (D-Phe), pyridinium *p*-toluenesulfonate (PPTS), and chloroform ($CHCl_3$) were purified and dried in accordance with standard procedures.[1] Estrone 3-methylether, phosphorous pentoxide (P_2O_5), sodium borohydride ($NaBH_4$), sodium hydride (NaH), ethanol (EtOH), pyridinium chlorochromate (PCC), platinum(IV) oxide (PtO_2), tetra-*n*-butylammonium fluoride hydrate (TBAF·xH_2O), Celite 545, as well as HPLC-grade pentane, hexanes, and ethyl acetate (EtOAc) used in column chromatography were purchased from Sigma-Aldrich and used without purification. Sodium chloride (NaCl), ammonium chloride (NH_4Cl), sodium bicarbonate ($NaHCO_3$), potassium carbonate (K_2CO_3), sodium hydroxide (NaOH), sodium sulfate (Na_2SO_4), sodium thiosulfate ($Na_2S_2O_3$), and magnesium sulfate ($MgSO_4$) were purchased from Fisher Scientific and used without further purification. Methyltriphenylphosphonium iodide was prepared from triphenylphosphine (Sigma-Aldrich), and methyl iodide (Sigma-Aldrich) by stirring in dry benzene for 2 hours, filtering, washing with hexanes, and drying over P_2O_5 before use. Molecular sieves (3Å 4-8 mesh) were purchased from Sigma-Aldrich and activated by drying under vacuum (approx. 30 mm Hg) at 250 °C for at least 6 hours prior to use. Rhodium chloride hydrate ($RhCl_3 \cdot H_2O$) was purchased from Pressure Chemical Company and used without further purification.

(1) Armarego, W. L. F.; Chai, C. L. *Purification of Laboratory Chemicals*, 5th ed.; Butterworth-Heinemann: Oxford, 2003.

Dess-Martin Periodinane (DMP) was synthesized in accordance with a reported literature procedure.[2] Scandium triflate ($Sc(OTf)_3$, 99%) was purchased from Sigma-Aldrich, finely powdered, and then dried at 200 °C over P_2O_5 for 24 hours under high vacuum (0.1 mm Hg). The dry scandium triflate was then transported into a glovebox using rigorous Schlenk techniques. (Trimethylsilyl)diazomethane (TMSD) and (phenyldimethylsilyl)diazomethane (PDMSD) were obtained as discussed in the manuscript and stored over 3Å molecular sieves at −40 °C in a glovebox freezer. Note: TMSD is both non-explosive and non-mutagenic, but it is extremely toxic[3] and must be handled with the appropriate precautions.

Instrumentation

Infrared spectra were recorded on a Bruker Alpha-p spectrometer. Bands are reported as strong (s), medium (m), weak (w), broad strong (bs), broad medium (bm), and broad weak (bw). Optical rotation values were recorded on a Rudolph research Autopol IV automatic polarimeter and is reported as the average of five readings. Melting points were recorded on a Digimelt MPA160 SRS and are uncorrected. Sonication was performed with a Misonix Sonicator 3000 equipped with a Laude external circulator for temperature control. ^{1}H NMR spectra were recorded on a Varian VNMRS (500 MHz), INOVA (500 MHz), or VNMRS (400 MHz) spectrometer. Chemical shifts are reported in ppm from tetramethyl-silane with the solvent resonance as the internal standard ($CHCl_3$: δ 7.26). Data are reported as follows: chemical shift, multiplicity (s = singlet, d = doublet, dd = doublet of doublets, ddd = doublet of doublet of doublets, dddd = doublet of doublet of doublet of doublets, t = triplet, m = multiplet), coupling constants (Hz), and integration. ^{13}C NMR spectra were recorded on a Varian VNMRS (125 MHz), INOVA (125 MHz), or VNMRS (100 MHz) spectrometer with complete proton decoupling. Chemical shifts are reported in ppm from tetramethylsilane with solvent as the internal reference ($CDCl_3$: δ 77.16). High-resolution mass spectra were obtained at the Boston College Mass Spectrometry Facility. Supercritical fluid chromatography (SFC) data were obtained on a Berger Instruments system using a Daicel CHIRALPAK AS-H column (ϕ 4.6 mm, 25 cm length). Gas chromatography (GC) analysis was performed on an Agilent Technologies 7890A system equipped with a flame ionization detector and HP-5 column (30 m x 0.320 mm x 0.25 μm).

(2) Meyer, S. D.; Schreiber, S. L. Acceleration of the Dess-Martin Oxidation by Water. *J. Org. Chem.* **1994**, *59*, 7549-7552.
(3) Murphy, N. G.; Varney, S. M.; Tallon, J. M.; Thompson, J. R.; Blanc, P. D. Fatal Occupational Exposure to Trimethylsilyl-Diazomethane. *Clin. Toxicol.* **2009**, *47*, 712.

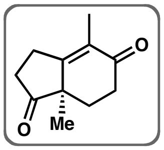

(*R*)-4,7a-Dimethyl-2,3,7,7a-tetrahydro-1*H*-indene-1,5(6*H*)-dione (precursor to **8**). A 40 mL vial (95 mm x 25 mm) equipped with a magnetic stir bar and a rubber septum was charged with 2-methyl-2-(3-oxopentyl)cyclopentane-1,3-dione[4] (2.00 g, 10.2 mmol, 1.00 equiv), D-Phe (505 mg, 3.06 mmol, 0.300 equiv), and PPTS (1.28 g, 5.09 mmol, 0.499 equiv). DMSO (0.73 mL) was added through a syringe, and the resulting suspension was stirred for 5 minutes at room temperature. The vial was then tightly sealed with a Teflon-lined screw cap and sonicated (60 W) continuously at 50 °C for 24 hours. After 20 minutes of sonication at 50 °C, the reaction mixture was observed to be dark yellow and homogeneous. The crude reaction mixture was directly loaded onto a flash column and eluted with 50% Et_2O in pentane (v/v) to furnish the desired product as a colorless oil (1.61 g, 88.6%) with 91% ee (AS-H, 50 °C, 150 psi, 1.0 mL/min, 3% MeOH, λ = 220 nm; t_R = 10.06 min (minor), 10.80 min (major)).

R_f = 0.50 (60% Et_2O in pentane v/v); ^{1}H NMR ($CDCl_3$, 500 MHz) δ 2.96-2.87 (m, 1H), 2.85-2.73 (m, 2H), 2.60-2.37 (m, 3H), 2.07 (ddd, *J* = 13.4, 5.1, 2.2 Hz, 1H), 1.85 (ddd, *J* = 13.9, 13.9, 5.9 Hz, 1H), 1.78 (d, *J* = 1.2 Hz, 3H), 1.29 (s, 3H); ^{13}C NMR ($CDCl_3$, 100 MHz) δ 217.74, 197.99, 162.55, 129.95, 48.99, 35.54, 32.92, 28.94, 24.60, 21.38, 10.89; HRMS (ESI+) Calcd. for $C_{11}H_{15}O_2$ $[M+H]^+$: 179.1072; Found 179.1076.

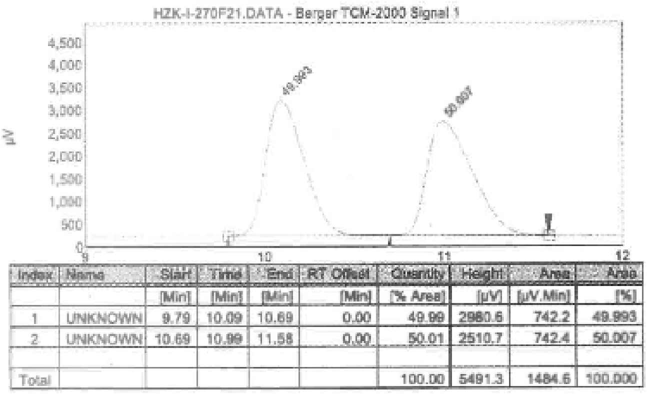

Index	Name	Start	Time	End	RT Offset	Quantity	Height	Area	Area
		[Min]	[Min]	[Min]	[Min]	[% Area]	[µV]	[µV.Min]	[%]
1	UNKNOWN	9.79	10.09	10.69	0.00	49.99	2980.6	742.2	49.993
2	UNKNOWN	10.69	10.99	11.58	0.00	50.01	2510.7	742.4	50.007
Total						100.00	5491.3	1484.6	100.000

HZK-I-270F21 -- AcqDate: 4/8/2010 4:32:08 PM
Description: AS-H, 50C, 150 psi, 1 mL/min, 3% MeOH, 220 nm, sample MeOH

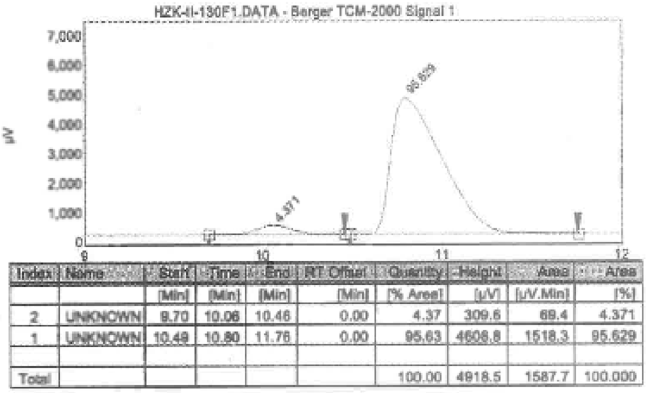

Index	Name	Start	Time	End	RT Offset	Quantity	Height	Area	Area
		[Min]	[Min]	[Min]	[Min]	[% Area]	[µV]	[µV.Min]	[%]
2	UNKNOWN	9.70	10.06	10.46	0.00	4.37	309.6	69.4	4.371
1	UNKNOWN	10.49	10.80	11.76	0.00	95.63	4608.8	1518.3	95.629
Total						100.00	4918.5	1587.7	100.000

HZK-II-130F1 -- AcqDate: 4/8/2010 4:50:39 PM
Description: AS-H, 50C, 150 psi, 1 mL/min, 3% MeOH, 220 nm, sample MeOH

Figure S1. Supercritical fluid chromatography (SFC) trace for Hajos–Parrish ketone.

(4) Hajos, Z. G.; Parrish, D. R. (+)-(7*aS*)-7a-Methyl-2,3,7,7a-tetrahydro-1*H*-indene-1,5-(6*H*)-dione. *Org. Synth.* **1990**, *Coll. Vol. 7*, 363.

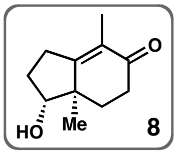

(1*R*,7a*R*)-1-Hydroxy-4,7a-dimethyl-2,3,7,7a-tetrahydro-1*H*-inden-5(6*H*)-one (8). The Hajos–Parrish ene-dione (3.49 g, 19.6 mmol, 1.00 equiv) was dissolved in 70 mL of EtOH, and the resulting homogeneous solution was cooled to −25 °C. Sodium borohydride (0.233 g, 6.16 mmol, 0.314 equiv) was added directly as a solid and the reaction was closely monitored by TLC. After 20 minutes, the reaction was judged to be complete and was quenched by the addition of saturated aqueous NaCl (30 mL) and H_2O (20 mL). The mixture was transferred to a separatory funnel and the product was extracted with Et_2O (3 x 50 mL). The combined organic layers were washed with saturated aqueous NaCl (50 mL), dried over Na_2SO_4, filtered, and concentrated. Purification by flash column chromatography (85% Et_2O in pentane v/v) afforded the desired product as a white solid (3.34 g, 94.5%). Enantioenrichment was achieved by a recrystallization from hot Et_2O and hexanes (approx. 3:1 v/v) to afford product **8** in optically pure form (2.14 g, 60.6%, 99% ee). (AS-H, 50 °C, 150 psi, 3.0 mL/min, 3% MeOH, λ = 220 nm; t_R = 16.27 min (major), 18.03 min (minor)).

R_f = 0.38 (60% EtOAc in hexanes v/v); ^{1}H NMR ($CDCl_3$, 500 MHz) δ 3.83 (ddd, *J* = 13.2, 7.3, 5.9 Hz, 1H), 2.62-2.52 (m, 2H), 2.46-2.36 (m, 2H), 2.19-2.11 (m, 1H), 2.07 (ddd, *J* = 12.7, 5.4, 2.0 Hz, 1H), 1.88-1.74 (m, 2H), 1.66 (dd, *J* = 1.2 Hz, 3H), 1.32 (s, 3H); ^{13}C NMR ($CDCl_3$, 125 MHz) δ 198.96, 168.10, 129.09, 81.05, 45.15, 34.11, 33.41, 29.60, 25.76, 15.34, 10.80; HRMS (ESI+) Calcd. for $C_{11}H_{17}O_2$ [M+H]$^+$: 181.1229; Found 181.1220.

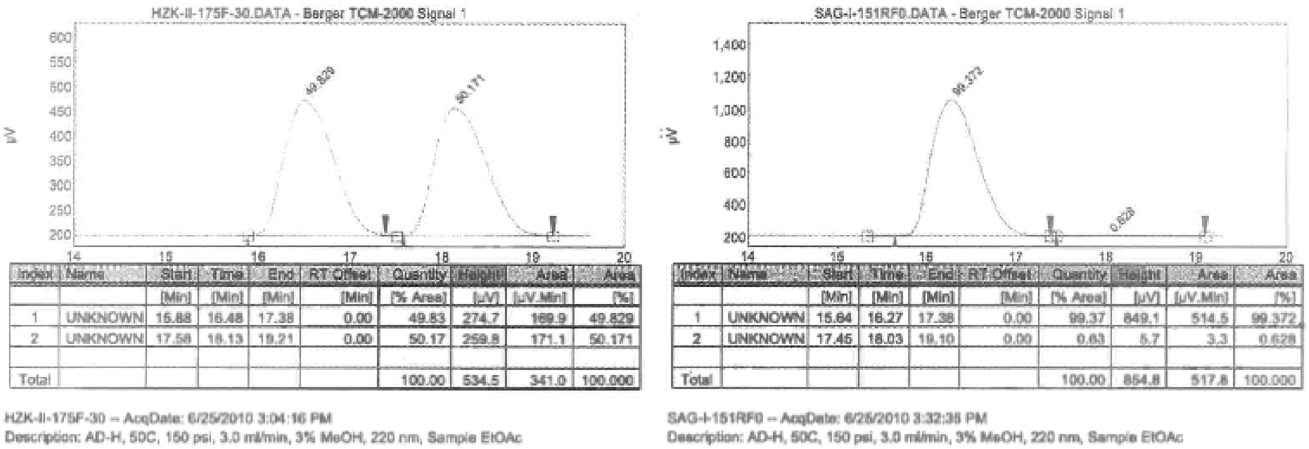

Index	Name	Start	Time	End	RT Offset	Quantity	Height	Area	Area
		[Min]	[Min]	[Min]	[Min]	[% Area]	[µV]	[µV.Min]	[%]
1	UNKNOWN	15.88	16.48	17.38	0.00	49.83	274.7	169.9	49.829
2	UNKNOWN	17.58	18.13	19.21	0.00	50.17	259.8	171.1	50.171
Total						100.00	534.5	341.0	100.000

HZK-II-175F-30 -- AcqDate: 6/25/2010 3:04:16 PM
Description: AD-H, 50C, 150 psi, 3.0 ml/min, 3% MeOH, 220 nm, Sample EtOAc

Index	Name	Start	Time	End	RT Offset	Quantity	Height	Area	Area
		[Min]	[Min]	[Min]	[Min]	[% Area]	[µV]	[µV.Min]	[%]
1	UNKNOWN	15.64	16.27	17.38	0.00	99.37	849.1	514.5	99.372
2	UNKNOWN	17.45	18.03	19.10	0.00	0.63	5.7	3.3	0.628
Total						100.00	854.8	517.8	100.000

SAG-I-151RF0 -- AcqDate: 6/25/2010 3:32:35 PM
Description: AD-H, 50C, 150 psi, 3.0 ml/min, 3% MeOH, 220 nm, Sample EtOAc

Figure S2. Supercritical fluid chromatography (SFC) trace for tetrahydroindanol **8**.

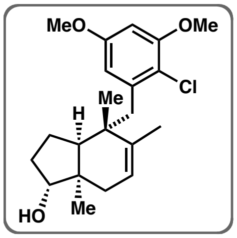

(1*R*,3a*S*,4*S*,7a*R*)-4-(2-Chloro-3,5-dimethoxybenzyl)-4,5,7a-trimethyl-2,3,3a,4,7,7a-hexahydro-1*H*-inden-1-ol (intermediate precursor to **11**). Exocyclic methylene **10**[5] (1.00 g, 2.09 mmol, 1.00 equiv) and $RhCl_3 \cdot H_2O$ (87.3 mg, 0.417 mmol, 0.200 equiv) were weighed into a 100 mL round bottom flask equipped with a magnetic stir bar and dissolved in 21 mL of $CHCl_3$ and 21 mL of EtOH. The resulting deep red solution was refluxed for a period of 2.5 days, during which time the solution got darker in color and a metallic precipitate formed. The reaction mixture was concentrated, and the crude residue was purified by flash column chromatography (60% Et_2O in pentane v/v) to afford the desired desilylated product as a white solid (749 mg, 98.2%), mp 44–48 °C.

$[\alpha]^{20}_D$ = −130.65 (c 0.39, $CHCl_3$); R_f = 0.36 (50% Et_2O in pentane v/v); ^{1}H NMR ($CDCl_3$, 500 MHz) δ 6.45 (d, *J* = 2.7 Hz, 1H), 6.38 (d, *J* = 2.7 Hz, 1H), 5.58-5.62 (m, 1H), 3.86 (s, 3H), 3.76 (s, 3H), 3.56 (ddd, *J* = 8.8, 6.1, 6.1 Hz, 1H), 3.19 (d, *J* = 12.9 Hz, 1H), 2.75 (d, *J* = 13.2 Hz, 1H), 2.12-1.95 (m, 3H), 1.85 (dddd, *J* = 6.1, 6.1, 6.1, 6.1 Hz, 1H), 1.73-1.66 (m, 1H), 1.46 (s, 3H), 1.42-1.32 (m, 2H), 1.16 (s, 3H), 1.09-0.99 (m, 1H), 0.94 (s, 3H); ^{13}C NMR ($CDCl_3$, 125 MHz) δ 158.02, 155.65, 141.40, 139.51, 122.22, 116.08, 108.25, 97.96, 82.56, 56.29, 55.95, 55.53, 43.45, 42.90, 39.97, 34.98, 31.60, 26.58, 25.23, 22.14, 21.50; IR (neat) 3407 (bm), 2956 (m), 2873 (m), 1590 (s), 1455 (s), 1329 (m), 1202 (m), 1162 (s), 1118 (m), 1036 (m), 811 (w) cm^{-1}; HRMS (ESI+) Calcd. for $C_{21}H_{30}ClO_3$ $[M+H]^+$: 365.1884; Found 365.1879.

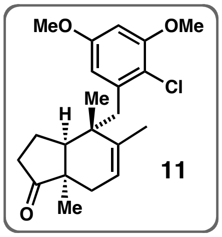

(3a*S*,4*S*,7a*R*)-4-(2-Chloro-3,5-dimethoxybenzyl)-4,5,7a-trimethyl-2,3,3a,4,7,7a-hexahydro-1*H*-inden-1-one (**11**). The endocyclic ene-carbinol (610 mg, 1.67 mmol, 1.00 equiv) and Celite 545 (720 mg) were weighed into a 25 mL round bottom flask equipped with a magnetic stir bar and suspended in 8.4 mL of CH_2Cl_2. PCC (721 mg, 3.34 mmol, 2.00 equiv) was then added directly as a solid, causing a black discoloration, and the mixture was stirred at room temperature for 2 hours. The reaction mixture was then diluted with 50 mL of Et_2O, filtered through Celite 545 on a sintered glass frit, and concentrated. The crude extract was purified by flash column chromatography (25% Et_2O in pentanes v/v) to furnish the desired cyclopentanone **11** as a white solid (561 mg, 92.6%), mp 130–135 °C.

(5) Prepared as previously reported, see: Kaplan, H. Z.; Rendina, V. L.; Kingsbury, J. S. Diastereoselective synthesis of complex *cis*-hexahydroindanes by reductive alkylation. *J. Org. Chem.* **2013**, *78*, 4620-4626.

$[\alpha]^{20}_D$ = −99.36 (c 1.68, $CHCl_3$); R_f = 0.29 (20% Et_2O in pentane v/v); ^1H NMR ($CDCl_3$, 500 MHz) δ 6.41 (d, *J* = 2.7 Hz, 1H), 6.40 (d, *J* = 2.7 Hz, 1H), 5.51-5.47 (m, 1H), 3.88 (s, 3H), 3.76 (s, 3H), 3.24 (d, *J* = 13.2 Hz, 1H), 2.80 (d, *J* = 13.2 Hz, 1H), 2.35-2.25 (m, 2H), 2.25-2.15 (m, 2H), 1.99-1.91 (m, 2H), 1.54-1.52 (m, 3H), 1.41-1.32 (m, 4H), 1.03 (s, 3H); ^{13}C NMR ($CDCl_3$, 125 MHz) δ 223.25, 158.10, 155.82, 139.81, 138.97, 120.87, 116.05, 108.61, 97.88, 56.30, 55.54, 53.45, 47.24, 42.34, 40.94, 36.15, 31.39, 26.91, 23.98, 21.51, 21.27; IR (neat) 2964 (m), 2937 (m), 2839 (w), 1737 (s), 1590 (s), 1455 (s), 1330 (m), 1205 (m), 1163 (s), 1086 (m), 1036 (m) cm^{-1}; HRMS (ESI+) Calcd. for $C_{21}H_{28}ClO_3$ $[M+H]^+$: 363.1727; Found 363.1726.

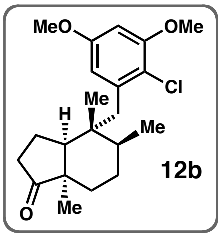

(3a*S*,4*R*,5*S*,7a*R*)-4-(2-Chloro-3,5-dimethoxybenzyl)-4,5,7a-trimethyloctahydro-1*H*-inden-1-one (**12b**). To a solution of racemic trisubstituted enone **11** (18.6 mg, 0.0513 mmol, 1.00 equiv) in 0.30 mL of CH_2Cl_2 at room temperature, PtO_2 (1.2 mg, 0.0051 mmol, 0.10 equiv) was added as a solid. With vigorous stirring, the suspension was purged for 1 minute with hydrogen from a balloon, at which point the brown PtO_2 catalyst turned black, signifying reduction to active Pt(0). The reaction was stirred for 3.5 hours under a positive pressure of hydrogen and then filtered through Celite 545. Upon removal of solvent, ^1H NMR analysis of the crude mixture showed incomplete conversion, so the material was re-subjected to the reaction conditions. Following another 6 hours of stirring under hydrogen, the suspension was again filtered and concentrated. Purification by silica gel chromatography (15% Et_2O, 75% pentane, 10% CH_2Cl_2 v/v/v) gave the desired β-methyl product **12b** as a white solid (12.1 mg, 64.6%), mp 131–133 °C. Single crystals for X-ray diffraction were obtained upon recrystallization from hot Et_2O and hexanes (approx. 5:1 v/v).

R_f = 0.43 (30% Et_2O in pentane v/v); ^1H NMR ($CDCl_3$, 500 MHz) δ 6.42 (d, *J* = 2.9 Hz, 1H), 6.41 (d, *J* = 2.7 Hz, 1H), 3.89 (s, 3H), 3.80 (s, 3H), 3.02 (d, *J* = 13.7 Hz, 1H), 2.63 (d, *J* = 13.7 Hz, 1H), 2.32-2.23 (m, 1H), 2.09-1.97 (m, 3H), 1.88 (d, *J* = 7.6 Hz, 1H) 1.79-1.70 (m, 1H), 1.53-1.45 (m, 1H), 1.33-1.27 (m, 1H), 1.20-1.04 (m, 2H), 1.00 (d, *J* = 6.8 Hz, 3H), 0.93 (s, 3H), 0.71 (s, 3H); ^{13}C NMR ($CDCl_3$, 125 MHz) δ 222.16, 158.01, 155.88, 138.93, 116.17, 108.63, 97.80, 56.33, 55.55, 49.60, 49.39, 42.14, 41.71, 37.07, 34.74, 30.23, 27.81, 27.41, 21.05, 17.37, 15.89; IR (neat) 2961 (m), 2926 (m), 1732 (s), 1589 (s), 1454 (s), 1329 (m), 1202 (s), 1164 (s), 1087 (m), 1038 (m) cm^{-1}; HRMS (ESI+) Calcd. for $C_{21}H_{30}ClO_3$ $[M+H]^+$: 365.1884; Found 365.1895.

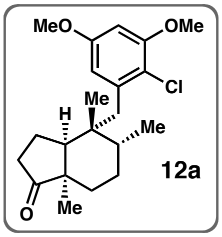

(3a*S*,4*R*,5*R*,7a*R*)-4-(2-Chloro-3,5-dimethoxybenzyl)-4,5,7a-trimethyloctahydro-1*H*-inden-1-one (**12a**). Recovered from the hydrogenation reaction above, the α-methyl diastereomer **12a** was isolated a white solid (6.3 mg, 33.7%), mp 147–149 °C. Single crystals for X-ray diffraction were obtained upon recrystallization from hot Et_2O and hexanes (approx. 5:1 v/v).

R_f = 0.31 (15% Et_2O, 10% CH_2Cl_2 in pentane v/v/v); ^{1}H NMR ($CDCl_3$, 500 MHz) δ 6.46 (d, *J* = 2.9 Hz, 1H), 6.40 (d, *J* = 2.9 Hz, 1H), 3.88 (s, 3H), 3.78 (s, 3H), 3.07 (d, *J* = 13.4 Hz, 1H), 2.82 (d, *J* = 13.7 Hz, 1H), 2.41 (ddd, *J* = 19.3, 8.5, 2.4 Hz, 1H), 2.28 (ddd, *J* = 10.5, 7.0, 0 Hz, 1H), 2.19-2.10 (m, 1H), 1.87-1.74 (m, 2H), 1.74-1.65 (m, 1H), 1.51 (m, 3H), 1.38 (s, 3H), 1.27-1.21 (m, 1H), 1.10 (d, *J* = 7.0 Hz, 3H), 0.76 (s, 3H); ^{13}C NMR ($CDCl_3$, 125 MHz) δ 222.87, 158.05, 156.01, 139.90, 116.15, 109.01, 97.26, 56.32, 55.58, 49.76, 48.71, 39.01, 37.49, 35.86, 35.01, 28.95, 26.32, 23.85, 22.18, 20.86, 16.19; IR (neat) 2963 (m), 2877 (m), 1732 (s), 1590 (s), 1455 (s), 1327 (m), 1201 (s), 1162 (s), 1092 (m), 1035 (m), 732 (m) cm^{-1}; HRMS (ESI+) Calcd. for $C_{21}H_{30}ClO_3$ $[M+H]^+$: 365.1884; Found 365.1885.

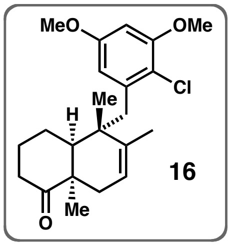

(4a*S*,5*S*,8a*R*)-5-(2-Chloro-3,5-dimethoxybenzyl)-5,6,8a-trimethyl-3,4,4a,5,8,8a-hexahydronaphthalen-1(2*H*)-one (**16**). In a glovebox $Sc(OTf)_3$ (5.2 mg, 0.011 mmol, 0.052 equiv) was weighed directly into a 1.5 mL vial equipped with a magnetic stir bar. A solution of cyclopentanone **11** (76.6 mg, 0.211 mmol, 1.00 equiv) in $CDCl_3$ (0.8 mL) was transferred directly to the solid $Sc(OTf)_3$. The cloudy gray suspension was stirred for 15 minutes, at which point TMSD (215 μL of a 1.96 M solution in hexanes, 0.422 mmol, 2.00 equiv) was added dropwise. The entire reaction mixture (including any residual solids) was transferred via glass pipette to a J. Young NMR tube, and the vial was rinsed with an additional 0.2 mL of $CDCl_3$. The vessel was removed from the glovebox, connected to a nitrogen manifold, and placed in an oil bath at 50 °C. After 16 hours of heating, the reaction was cooled to room temperature. ^{1}H NMR analysis indicated full conversion and an approximately 8.5:1 ratio of regioisomeric enoltrimethylsilane products. The mixture was rinsed from the NMR tube with Et_2O (5 mL) and concentrated to give a yellow oil. The residue was immediately dissolved in 4 mL of 1:1 (v/v) 1N HCl: THF and stirred for 2 hours. That solution was then poured into saturated $NaHCO_3$ (20 mL) and extracted with Et_2O (3 x 20 mL). The organic layer was washed with saturated aqueous NaCl (50 mL), dried over Na_2SO_4, filtered, and concentrated. Purification by chromatography (18% EtOAc in hexanes v/v) gave homologous cyclohexanone **16** (71.1 mg, 88.9%) and a minor amount of the minor regioisomer (8.6 mg, 10.8%) as colorless oils.

$[\alpha]^{20}_{D}$ = +4.12 (c 1.33, $CHCl_3$); R_f = 0.35 (18% EtOAc in hexanes); ^{1}H NMR ($CDCl_3$, 500 MHz) δ 6.46 (d, *J* = 2.7 Hz, 1H), 6.39 (d, *J* = 2.7 Hz, 1H), 5.54-5.51 (m, 1H), 3.87 (s, 3H), 3.74 (m, 3H), 3.22 (d, *J* = 14.4 Hz, 1H), 2.83 (d, *J* = 14.4 Hz, 1H), 2.63-2.56 (m , 1H), 2.48-2.44 (m, 1H), 2.12 (ddd, *J* = 8.1, 4.9, 0 Hz, 1H), 2.09-2.03 (m, 1H), 1.93-1.86 (m, 1H), 1.81-1.72 (m, 4H), 1.64-1.53 (m, 2H), 1.34 (s, 3H), 1.01 (s, 3H); ^{13}C NMR ($CDCl_3$, 125 MHz) δ 216.38, 158.10, 155.76, 139.31, 138.47, 121.64, 115.92, 107.47, 97.82, 56.29, 55.52, 49.03, 48.89, 42.66, 40.15, 36.66, 32.58, 26.44, 24.27, 24.05, 23.22, 20.19; IR (neat) 2963 (m), 2940 (m), 1701 (s), 1590 (s), 1454 (s), 1330 (m), 1203 (s), 1163 (s), 1087 (m), 1036 (w), 830 (w) cm^{-1}; HRMS (ESI+) Calcd. for $C_{22}H_{30}ClO_3$ $[M+H]^+$: 377.1884; Found 377.1891.

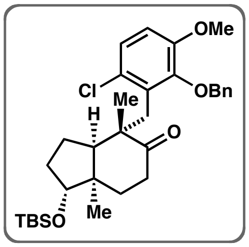

(1*R*,3a*R*,4*S*,7a*R*)-4-(2-(Benzyloxy)-6-chloro-3-methoxybenzyl)-1-(*tert*-butyldimethylsilyloxy)-4,7a-dimethylhexahydro-1*H*-inden-5(6*H*)-one (precursor to **17**). To a solution of the corresponding keto carbinol[5] (1.03 g, 2.32 mmol, 1.00 equiv) in 12 mL of DMF were added imidazole (474 mg, 6.97 mmol, 3.00 equiv) and TBSCl (1.05 g, 6.97 mmol, 3.00 equiv) sequentially as solids. After 5 hours of stirring at room temperature, 5 mL of MeOH was added and the reaction was stirred for an additional 15 minutes. The reaction mixture was then poured into saturated NH_4Cl (20 mL) and the product was extracted with Et_2O (5 x 10 mL). The organic layer was washed with 1 N HCl (30 mL), H_2O (30 mL), and saturated aqueous NaCl (30 mL) before being dried over Na_2SO_4, filtered, and concentrated to give the target silyl ether as a thick oil that was used without further purification (1.14 g, 88.4%).

$[\alpha]^{20}_{D}$ = −31.95 (c 0.87, $CHCl_3$); ^{1}H NMR ($CDCl_3$, 500 MHz) δ 7.40-7.32 (m, 5H), 7.04 (d, *J* = 8.8 Hz, 1H), 6.76 (d, *J* = 9.0 Hz, 1H), 5.02 (d, *J* =11.2 Hz, 1H), 4.91 (d, *J* = 11.0 Hz, 1H), 3.83 (s, 3H), 3.66 (dd, *J* = 7.3, 5.9 Hz, 1H), 3.42 (d, *J* = 13.4 Hz, 1H), 2.86 (d, *J* = 13.4 Hz, 1H), 2.61 (ddd, *J* = 17.4, 5.9, 5.9 Hz, 1H), 2.07-1.97 (m, 2H), 1.84-1.77 (m, 1H), 1.77-1.67 (m, 2H), 1.52-1.44 (m, 1H), 1.44-1.36 (m, 1H), 1.10 (s, 3H), 0.91 (s, 9H), 0.80 (s, 3H), 0.03-0.02 (m, 6H); ^{13}C NMR ($CDCl_3$, 125 MHz) δ 215.05, 151.19, 147.94, 137.56, 130.67, 128.79, 128.55, 128.32, 127.28, 124.39, 111.91, 80.62, 75.12, 56.89, 56.09, 52.03, 42.41, 37.09, 35.24, 32.13, 32.06, 27.20, 26.01, 25.26, 19.14, 18.24, −4.21, −4.77; IR (neat) 2956 (bs), 2876 (bm), 1705 (s), 1465 (bs), 1377 (bw), 1277 (s), 1214 (m), 1115 (bm), 1060 (s), 981 (bm), 836 (s), 775 (s), 698 (m) cm^{-1}; HRMS (ESI+) Calcd. for $C_{32}H_{46}ClO_4Si$ $[M+H]^+$: 557.2854; Found 557.2836.

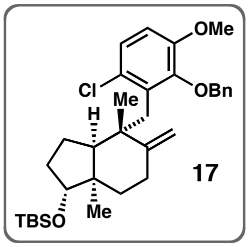

((1*R*,3a*S*,4*S*,7a*R*)-4-(2-(Benzyloxy)-6-chloro-3-methoxybenzyl)-4,7a-dimethyl-5-methyleneoctahydro-1*H*-inden-1-yloxy)(*tert*-butyl)dimethylsilane (17). In a glovebox, NaH (92.6 mg, 3.86 mmol, 7.00 equiv) was added to a 2-neck, 25 mL round bottom flask equipped with a magnetic stir bar. Upon removing the flask from the glovebox, a reflux condenser was installed. DMSO (4.2 mL) was added and the suspension was heated at 75 °C for 1 hour. During this time, the reaction became homogeneous, forming a teal-colored, clear solution. This solution was cooled to ambient temperature and a solution of Ph_3PCH_3I (2.01 g, 4.96 mmol, 9.00 equiv) in 6.8 mL of DMSO was added over 30 minutes via syringe pump. After addition of the salt solution, the reaction mixture turned bright yellow. Upon completion of the addition, the mixture was stirred for an additional 30 minutes at room temperature, at which point a solution of the cyclohexanone (307 mg, 0.551 mmol, 1.00 equiv) in 1.5 mL of DMSO and 1.5 mL of THF was added dropwise. The reaction mixture was then heated to 75 °C and stirred for 16 hours. The resulting amber solution was cooled to room temperature and acidified by the addition of 5 mL of saturated aqueous NH_4Cl. The reaction mixture was then diluted with H_2O (15 mL), transferred to a separatory funnel, and extracted with Et_2O (3 x 20 mL). The organic layer was washed with H_2O (25 mL) and saturated aqueous NaCl (20 mL) before being dried over Na_2SO_4, filtered, and concentrated. Purification by silica gel chromatography (20% Et_2O in pentane v/v) afforded the desired compound **17** as a white solid (296 mg, 96.7%), mp 98–105 °C.

$[\alpha]^{20}_D$ = +15.37 (c 1.05, $CHCl_3$); R_f = 0.33 (3% Et_2O in pentane v/v); ^{1}H NMR ($CDCl_3$, 500 MHz) δ 7.43-7.29 (m, 5H), 7.04 (d, *J* = 8.8 Hz, 1H) 6.73 (d, *J* = 8.8 Hz, 1H), 4.98-4.81 (m, 2H), 4.75-4.71 (m, 1H), 4.35-4.30 (m, 1H), 3.84 (s, 3H), 3.51 (dd, *J* = 5.9, 2.0 Hz, 1H), 3.36 (d, *J* = 13.2 Hz, 1H), 2.76-2.65 (m, 1H), 2.73 (d, *J* = 12.9 Hz, 1H), 2.05-1.93 (m, 2H), 1.93-1.84 (m, 1H), 1.77-1.68 (m, 1H), 1.43-1.34 (m, 1H), 1.30-1.20 (m, 1H), 1.20-1.09 (m, 2H), 1.14 (s, 3H), 0.92 (s, 9H), 0.82 (s, 3H), 0.04 (s, 3H), 0.03 (s, 3H); ^{13}C NMR ($CDCl_3$, 125 MHz) δ 151.93, 151.25, 148.43, 137.74, 133.02, 128.62, 128.49, 128.10, 127.82, 124.31, 111.03, 110.56, 83.86, 75.02, 56.04, 55.78, 45.67, 43.81, 38.05, 33.26, 31.72, 30.03, 26.65, 26.12, 23.10, 22.71, 18.32, −4.26, −4.69; IR (neat) 2954 (bs), 2933 (bs), 2856 (bm), 1463 (s), 1438 (m), 1371 (bw), 1277 (bm), 1074 (bs), 1006 (m), 836 (s), 740 (m), 697 (m) cm^{-1}; HRMS (ESI+) Calcd. for $C_{33}H_{48}ClO_3Si$ $[M+H]^+$: 555.3061; Found 555.3084.

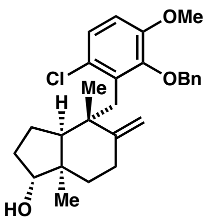

(1*R*,3a*S*,4*S*,7a*R*)-4-(2-(Benzyloxy)-6-chloro-3-methoxybenzyl)-4,7a-dimethyl-5-methyleneoctahydro-1*H*-inden-1-ol (intermediate precursor to **18**). To a solution of TBS ether **17** (1.27 g, 2.29 mmol, 1.00 equiv) in 5.7 mL of THF was added TBAF·xH_2O (10.5 g, 37.5 mmol, 16.4 equiv) as a solid. The resulting suspension was then sonicated (60 W) continuously at 50 °C for 12 hours, a period shortly into which the reaction mixture became homogeneous. The residue was then directly loaded onto a pad of silica gel and eluted with EtOAc to afford the desired exocyclic ene carbinol as a solid that was used without further purification (1.01 g, quantitative), mp 122–125 °C.

$[\alpha]^{20}_D$ = +34.99 (c 0.96, $CHCl_3$); R_f = 0.48 (50% EtOAc in hexanes v/v); ^{1}H NMR ($CDCl_3$, 500 MHz) δ 7.42-7.30 (m, 5H), 7.04 (d, *J* = 8.8 Hz, 1H), 6.73 (d, *J* = 8.8 Hz, 1H), 4.97-4.85 (m, 2H), 4.77-4.74 (m, 1H), 4.37-4.33 (m, 1H), 3.84 (s, 3H), 3.56 (ddd, *J* = 5.6, 4.2, 1.2 Hz, 1H), 3.33 (d, *J* = 12.9 Hz, 1H), 2.75-2.63 (m, 1H), 2.73 (d, *J* = 13.2 Hz, 1H), 2.04-1.92 (m, 3H), 1.81-1.72 (m, 1H), 1.46-1.38 (m, 1H), 1.37-1.27 (m, 2H), 1.20 (s, 3H), 1.19-1.12 (m, 1H), 0.82 (s, 3H); ^{13}C NMR ($CDCl_3$, 125 MHz) δ 151.56, 151.18, 148.45, 137.83, 132.73, 128.53, 128.47, 128.07, 127.77, 124.25, 111.06, 110.79, 83.99, 75.10, 56.02, 55.30, 45.32, 43.56, 37.89, 33.02, 30.71, 29.79, 26.45, 22.60, 21.87; IR (neat) 3377 (bw), 3058 (bw), 2917 (bw), 2848 (bw), 1647 (bw), 1479 (m), 1295 (bm), 1063 (bs), 925 (bm), 737 (s), 688 (bs) cm^{-1}; HRMS (ESI+) Calcd. for $C_{27}H_{34}ClO_3$ $[M+H]^+$: 441.2196; Found 441.2174.

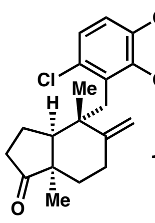

18

(3a*S*,4*S*,7a*R*)-4-(2-(Benzyloxy)-6-chloro-3-methoxybenzyl)-4,7a-dimethyl-5-methyleneoctahydro-1*H*-inden-1-one (**18**). The ene cyclopentanol (1.01 g, 2.29 mmol, 1.00 equiv) was weighed into a 50 mL round bottom flask equipped with a magnetic stir bar and dissolved in 23 mL of wet CH_2Cl_2. The solution was cooled to 4 °C and DMP (2.91 g, 6.87 mmol, 3.00 equiv) was added as a solid. The reaction mixture was stirred for 12 hours at 4 °C, at which point additional DMP (2.02 g, 4.58 mmol, 2.00 equiv) and 50 μL of H_2O were added. The reaction was warmed to room temperature and stirred for an additional hour. The mixture was then poured over 100 mL of 1 N NaOH and extracted with CH_2Cl_2 (3 x 25 mL). The combined organic layers were dried over Na_2SO_4, filtered, and concentrated. Purification by column chromatography (20% EtOAc in hexanes v/v) afforded cyclopentanone **18** as a white foam (1.00 g, quantitative), mp 95–98 °C.

$[\alpha]^{20}_{D}$ = +11.12 (c 1.17, $CHCl_3$); R_f = 0.27 (20% EtOAc in hexanes v/v); ^{1}H NMR ($CDCl_3$, 500 MHz) δ 7.40-7.30 (m, 5H), 7.06 (d, *J* = 8.8 Hz, 1H), 6.76 (d, *J* = 8.8 Hz, 1H), 4.98-4.86 (m, 2H), 4.80-4.77 (m, 1H), 4.43-4.39 (m, 1H), 3.86 (s, 3H), 3.34 (d, *J* = 12.9 Hz, 1H), 2.74 (d, *J* = 12.9 Hz, 1H), 2.71-2.60 (m, 1H), 2.38 (dd, *J* = 19.3, 8.5 Hz, 1H), 2.11-1.96 (m, 2H), 1.92-1.81 (m, 2H), 1.52-1.42 (m, 1H), 1.35 (ddd, *J* = 13.4, 13.4, 3.9 Hz, 1H), 1.23 (s, 3H), 1.20-1.12 (m, 1H), 0.89 (s, 3H); ^{13}C NMR ($CDCl_3$, 125 MHz) δ 222.21, 151.19, 150.50, 148.50, 137.64, 132.08, 128.64, 128.54, 128.27, 127.63, 124.34, 111.32, 111.24, 75.30, 57.35, 56.03, 48.82, 43.20, 38.17, 35.51, 30.61, 29.22, 21.81, 21.58, 21.49; IR (neat) 2935 (bm), 2856 (bw), 1734 (s), 1575 (w), 1464 (bs), 1406 (m), 1277 (s), 1234 (bm), 1072 (m), 978 (bm), 896 (bm), 798 (m), 698 (m) cm^{-1}; HRMS (ESI+) Calcd. for $C_{27}H_{32}ClO_3$ $[M+H]^+$: 439.2040; Found 439.2024.

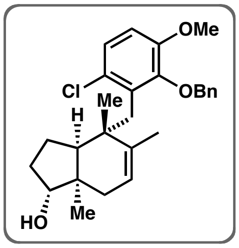

(1*R*,3a*S*,4*S*,7a*R*)-4-(2-(Benzyloxy)-6-chloro-3-methoxybenzyl)-4,5,7a-trimethyl-2,3,3a,4,7,7a-hexahydro-1*H*-inden-1-ol (intermediate precursor to **19**). The TBS ether **17** (263 mg, 0.473 mmol, 1.00 equiv) and $RhCl_3 \cdot H_2O$ (14.7 mg, 0.0702 mmol, 0.148 equiv) were added to a 5 mL 2-neck round bottom flask equipped with a magnetic stir bar and a reflux condenser. The solids were dissolved in 1.2 mL of EtOH and 1.2 mL of $CHCl_3$, and the resulting deep red solution was heated at 55 °C for 15 hours. The reaction mixture was cooled to ambient temperature and concentrated. The crude residue was purified by column chromatography (25% EtOAc in hexanes) to give the desired alcohol as a colorless oil that was used directly in the next step (208 mg, quantitative).

$[\alpha]^{20}_{D}$ = −103.49 (c 0.85, $CHCl_3$); R_f = 0.38 (30% EtOAc in hexanes v/v); ^{1}H NMR ($CDCl_3$, 500 MHz) δ 7.45-7.41 (m, 2H), 7.39-7.30 (m, 3H), 7.07 (d, *J* = 8.8 Hz, 1H), 6.75 (d, *J* = 8.8 Hz, 1H), 5.35-5.32 (m, 1H), 4.94 (d, *J* = 11.0 Hz, 1H), 4.88 (d, *J* = 11.0 Hz, 1H), 3.86 (s, 3H), 3.52 (ddd, *J* = 6.1, 6.1, 0 Hz, 1H), 3.24 (d, *J* = 12.9 Hz, 1H), 2.75 (d, *J* = 12.9 Hz, 1H), 2.07-2.01 (m, 1H), 1.92-1.85 (m, 1H), 1.84-1.76 (m, 2H), 1.67 (dddd, *J* = 15.3, 7.6, 7.6, 2.0 Hz, 1H), 1.47-1.43 (m, 3H), 1.39-1.30 (m, 2H), 1.14 (s, 3H), 1.11-1.02 (m, 1H), 0.87 (s, 3H); ^{13}C NMR ($CDCl_3$, 125 MHz) δ 151.49, 148.35, 140.37, 137.71, 133.32, 128.45, 128.40, 128.09, 127.78, 124.63, 121.62, 111.00, 82.91, 74.81, 56.01, 55.17, 42.96, 42.51, 36.88, 34.60, 31.52, 26.91, 24.69, 22.15, 21.29; IR (neat) 3389 (bw), 3064 (bw), 2955 (bm), 2873 (bm), 1574 (w), 1464 (bs), 1373 (m), 1276 (s), 1178 (m), 1074 (bm), 981 (bm), 797 (m), 697 (m) cm^{-1}; HRMS (ESI+) Calcd. for $C_{27}H_{33}ClO_3$ $[M+H]^+$: 441.2196; Found 441.2182.

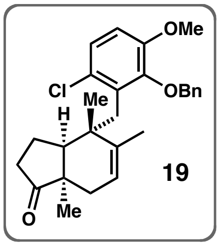

(3a*S*,4*S*,7a*R*)-4-(2-(Benzyloxy)-6-chloro-3-methoxybenzyl)-4,5,7a-trimethyl-2,3,3a,4,7,7a-hexahydro-1*H*-inden-1-one (**19**). The cyclopentanol above (208 mg, 0.473 mmol, 1.00 equiv) was weighed into a 10 mL round bottom flask equipped with a magnetic stir bar and dissolved in 4.7 mL of wet CH_2Cl_2. DMP (602 mg, 1.42 mmol, 3.00 equiv) was then added directly as a solid and the reaction mixture was stirred for 1.5 hours at room temperature. The mixture was then poured into 1 N NaOH (20 mL) and washed with CH_2Cl_2 (3 x 10 mL). The pooled organic layers were dried over Na_2SO_4, filtered, and concentrated. Purification by column chromatography (12% EtOAc in hexanes v/v) afforded the desired bicyclopentanone **19** as a colorless oil (203 mg, 97.8%, 2 steps).

$[\alpha]^{20}{}_D$ = −84.42 (c 0.95, $CHCl_3$); R_f = 0.33 (15% EtOAc in hexanes v/v); ^1H NMR ($CDCl_3$, 500 MHz) δ 7.40-7.30 (m, 5H), 7.09 (d, *J* = 8.8 Hz, 1H), 6.77 (d, *J* = 8.8 Hz, 1H), 5.29-5.26 (m, 1H), 4.96 (d, *J* = 11.0 Hz, 1H), 4.90 (d, *J* = 11.2 Hz, 1H), 3.87 (s, 3H), 3.20 (d, *J* = 13.2, Hz, 1H), 2.77 (d, *J* = 13.0 Hz, 1H), 2.32 (dd, *J* = 18.6, 7.6 Hz, 1H), 2.18 (dd, *J* = 11.7, 6.4 Hz, 1H), 2.13- 2.05 (m, 1H), 2.05-1.98 (m, 1H), 1.91-1.83 (m, 1H), 1.72 (dddd, *J* = 18.1, 2.0, 2.0, 2.0 Hz, 1H), 1.50-1.45 (m, 3H), 1.34 (dddd, *J* = 12.2, 12.2, 12.2, 8.5 Hz, 1H), 1.25 (s, 3H), 0.94 (s, 3H); ^{13}C NMR ($CDCl_3$, 125 MHz) δ 223.13, 151.52, 148.33, 139.33, 137.56, 132.77, 128.51, 128.40, 128.22, 127.75, 124.73, 119.92, 111.18, 74.92, 56.03, 54.11, 47.07, 41.61, 37.68, 36.04, 30.82, 25.03, 23.58, 21.89, 21.05; IR (neat) 2966 (bm), 2935 (bw), 1736 (s), 1464 (bs), 1372 (m), 1277 (s), 1242 (bm), 1076 (m), 984 (bm), 798 (m), 698 (m) cm^{-1}; HRMS (ESI+) Calcd. for $C_{27}H_{34}ClO_3$ $[M+H]^+$: 439.2040; Found 439.2037.

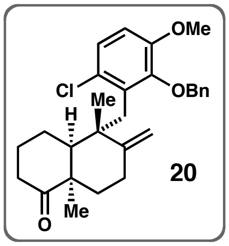

(4a*S*,5*S*,8a*R*)-5-(2-(Benzyloxy)-6-chloro-3-methoxybenzyl)-5,8a-dimethyl-6-methyleneoctahydronaphthalen-1(2*H*)-one (**20**). In a glovebox, $Sc(OTf)_3$ (4.2 mg, 0.0086 mmol, 0.050 equiv) was weighed directly into a J. Young NMR tube. A solution of cyclopentanone **18** (75.2 mg, 0.171 mmol, 1.00 equiv) in 0.48 mL of $CDCl_3$ was transferred directly to the solid $Sc(OTf)_3$. The cloudy gray suspension was allowed to stand for 15 minutes, at which point TMSD (174 μL of a 2.47 M solution in hexanes, 0.342 mmol, 2.00 equiv) was introduced dropwise. The NRM tube was removed from the glovebox, connected to a nitrogen manifold, and allowed to stand at room temperature for 12 hours. The reaction mixture was then warmed to 50 °C for 48 hours. ^1H NMR analysis indicated 98% conversion and a 5:1 ratio of regioisomeric enoltrimethylsilane products. The mixture was poured into H_2O (5 mL) and extracted with

Et_2O (20 mL). The organic layers were washed with saturated aqueous NaCl (10 mL), dried over Na_2SO_4, filtered, and concentrated. The enol silane products were then purified away from trace amounts of starting material by column chromatography (7% EtOAc in hexanes v/v). The flashed product mixture was then dissolved in 2 mL of THF and TBAF·xH_2O (95.8 mg, 0.342 mmol, 2.00 equiv) was added as a solid, at which point the reaction mixture was allowed to stand for 10 minutes at room temperature. The solution was concentrated and purified by column chromatography (15 to 25% EtOAc in hexanes v/v) to give the desired homologous exocyclic ene decalone **20** as a white solid (53.4 mg, 68.8%), mp 88–92 °C.

$[\alpha]^{20}_{D}$ = +8.23 (c 0.65, $CHCl_3$); R_f = 0.34 (15% EtOAc in hexanes v/v); ^1H NMR ($CDCl_3$, 500 MHz) δ 7.40-7.30 (m, 5H), 7.05 (d, *J* = 8.8 Hz, 1H), 6.75 (d, *J* = 8.8 Hz, 1H), 4.97 (d, *J* = 11.0 Hz, 1H), 4.88 (d, *J* = 11.0 Hz, 1H), 4.77-4.74 (m, 1H), 4.46-4.43 (m, 1H), 3.86 (s, 3H), 3.37 (d, *J* = 12.9 Hz, 1H), 2.82 (d, *J* = 12.9 Hz, 1H), 2.63 (ddd, *J* = 13.9, 4.4, 4.4 Hz, 1H), 2.45-2.37 (m, 1H), 2.33-2.26 (m, 1H), 2.12 (ddd, *J* = 14.6, 5.1, 5.1 Hz, 1H), 1.95-1.76 (m, 4H), 1.59-1.49 (m, 1H), 1.38-1.31 (m, 2H), 1.33 (s, 3H), 0.90 (s, 3H); ^{13}C NMR ($CDCl_3$, 125 MHz) δ 216.60, 151.80, 151.31, 148.28, 137.69, 132.98, 128.55, 128.25, 127.59, 124.43, 111.07, 110.30, 75.16, 56.00, 55.82, 50.90, 44.72, 39.92, 37.07, 32.57, 29.70, 24.24, 24.16, 22.82; IR (neat) 3087 (bw), 2938 (bm), 2861 (bm), 1698 (s), 1575 (w), 1462 (s), 1438 (m), 1372 (m), 1276 (s), 1214 (bm), 980 (bm), 798 (m), 698 (m) cm^{-1}; HRMS (ESI+) Calcd. for $C_{28}H_{34}ClO_3$ $[M+H]^+$: 453.2196; Found 453.2209.

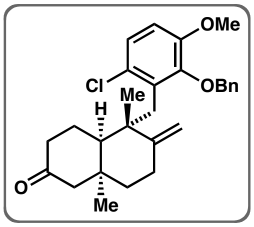

(4a*R*,5*S*,8a*S*)-5-(2-(Benzyloxy)-6-chloro-3-methoxybenzyl)-5,8a-dimethyl-6-methyleneoctahydronaphthalen-2(1*H*)-one (minor regioisomer in reaction of **18**). This material proved to be a colorless oil (6.5 mg, 8.4%).

$[\alpha]^{20}_{D}$ = +25.56 (c 0.59, $CHCl_3$); R_f = 0.25 (15% EtOAc in hexanes v/v); ^1H NMR ($CDCl_3$, 500 MHz) δ 7.42-7.32 (m, 5H), 7.6 (d, *J* = 8.8 Hz, 1H), 6.76 (d, *J* = 8.8 Hz, 1H), 5.01 (d, *J* = 10.2, 1H), 5.01 (d, *J* = 10.2 Hz, 1H), 4.89 (d, *J* = 11.2 Hz, 1H), 4.76-4.72 (m, 1H), 4.43-4.39 (m, 1H), 3.86 (s, 3H), 3.38 (d, *J* = 12.9 Hz, 1H), 2.88 (d, *J* = 13.2 Hz, 1H), 2.67-2.58 (m, 1H), 2.24 (d, *J* = 13.9, 1H), 2.24-2.18 (m, 2H), 2.08 (ddd, *J* = 14.6, 4.4, 4.4 Hz, 1H), 2.03-1.96 (m, 1H), 1.97 (d, *J* = 13.7, 1H), 1.76-1.69 (m, 1H), 1.52-1.44 (m, 2H), 1.31-1.24 (m, 1H), 1.18 (s, 3H), 0.90 (s, 3H); ^{13}C NMR ($CDCl_3$, 125 MHz) δ 212.83, 151.32, 151.06, 148.34, 137.92, 132.92, 128.55, 128.48, 128.19, 127.63, 124.42, 111.14, 110.74, 75.12, 56.20, 56.05, 53.03, 44.95, 40.33, 40.24, 39.50, 34.10, 31.46, 29.98, 26.03, 23.18; IR (neat) 3086 (bw), 2936 (bm), 2853 (bm), 1716 (s), 1464 (s), 1438 (bm), 1373 (bw), 1275 (s), 1215 (bm), 1102 (bm), 985 (bm), 798 (m), 698 (m) cm^{-1}; HRMS (ESI+) Calcd. for $C_{28}H_{34}ClO_3$ $[M+H]^+$: 453.2196; Found 453.2218.

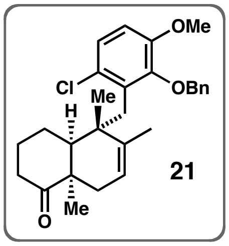

(4a*S*,5*S*,8a*R*)-5-(2-(Benzyloxy)-6-chloro-3-methoxybenzyl)-5,6,8a-trimethyl-3,4,4a,5,8,8a-hexahydronaphthalen-1(2*H*)-one (**21**). In a glovebox, $Sc(OTf)_3$ (6.5 mg, 0.015 mmol, 0.045 equiv) was added directly to a 1.5 mL vial equipped with a magnetic stir bar. A solution of bicyclopentanone **19** (128 mg, 0.292 mmol, 1.00 equiv) in $CDCl_3$ (0.53 mL) was added directly to the solid $Sc(OTf)_3$. The resulting cloudy gray suspension was stirred for 15 minutes, at which point TMSD (236 μL of a 2.47 M solution in hexanes, 0.583 mmol, 2.00 equiv) was added dropwise. The entire reaction mixture (including any residual solids) was then transferred via a glass pipette to a J. Young NMR tube, and the vial was rinsed with 0.2 mL of $CDCl_3$. The reaction tube was removed from the glovebox, connected to a nitrogen manifold, and submerged in an oil bath heated to 50 °C. After 16 hours of heating, the reaction was cooled to room temperature. ^{1}H NMR analysis indicated complete consumption of **19**. The reaction mixture was poured into H_2O (5 mL) and extracted with Et_2O (20 mL). The pooled organic layers were washed with saturated aqueous NaCl (10 mL), dried over Na_2SO_4, filtered, and concentrated. The residue was then dissolved in 2 mL of THF and after TBAF·xH_2O (164 mg, 0.584 mmol, 2.00 equiv) was added as a solid, the reaction mixture was allowed to stir for 10 minutes at 23 °C. The solution was concentrated and purified by chromatography (15% EtOAc in hexanes v/v) to give the desired homologous trisubstituted ene-decalone **21** as a colorless oil (124 mg, 93.4%).

$[\alpha]^{20}_D$ = −32.35 (c 0.83, $CHCl_3$); R_f = 0.57 (30% EtOAc in hexanes v/v); ^{1}H NMR ($CDCl_3$, 500 MHz) δ 7.42-7.39 (m, 2H), 7.38-7.30 (m, 3H), 7.09 (d, *J* = 8.8 Hz, 1H), 6.77 (d, *J* = 8.8 Hz, 1H), 5.36-5.32 (m, 1H), 4.96 (d, *J* = 10.7 Hz, 1H), 4.88 (d, *J* = 10.7 Hz, 1H), 3.88 (s, 3H), 3.14 (d, *J* = 13.7 Hz, 1H), 2.99 (d, *J* = 13.9 Hz, 1H), 2.50 (ddd, *J* = 14.6, 12.6, 6.8, 1H), 2.46-2.40 (m, 1H), 2.39-2.35 (m, 1H), 2.27-2.21 (m, 1H), 1.93-1.86 (m, 1H), 1.78-1.72 (m, 1H), 1.68-1.66 (m, 3H), 1.64-1.58 (m, 1H), 1.35 (s, 3H), 1.34-1.18 (m, 2H), 0.82 (s, 3H); ^{13}C NMR ($CDCl_3$, 125 MHz) δ 216.42, 151.57, 148.21, 139.89, 137.28, 133.52, 128.72, 128.56, 128.29, 127.58, 124.82, 119.27, 111.02, 74.83, 55.97, 51.23, 49.70, 42.12, 37.94, 37.23, 32.88, 24.73, 24.47, 23.74, 20.49; IR (neat) 3030 (bw), 2955 (bm), 2861 (bm), 1698 (s), 1574 (m), 1462 (bs), 1371 (m), 1276 (s), 1214 (bm), 1080 (bs), 979 (bs), 924 (bm), 797 (s), 732 (s), 697 (s) cm^{-1}; HRMS (ESI+) Calcd. for $C_{28}H_{34}ClO_3$ $[M+H]^+$: 453.2196; Found 453.2210.

14

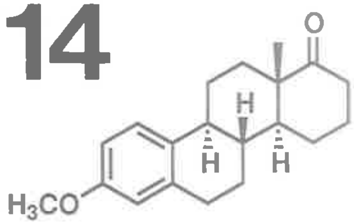

Sample Name:
VR-IV-014Fa
Archive directory:

Sample directory:

FidFile: VR-IV-014Fa

Pulse Sequence: Proton (s2pul)
Solvent: cdcl3
Data collected on: Aug 30 2011

Temp. 25.0 C / 298.1 K
Operator: jsk
INOVA-500 "nmr16"

Relax. delay 10.000 sec
Pulse 45.0 degrees
Acq. time 2.049 sec
Width 8012.8 Hz
9 repetitions
OBSERVE H1, 499.8808016 MHz
DATA PROCESSING
Resol. enhancement -0.0 Hz
FT size 65536
Total time 3 min 37 sec

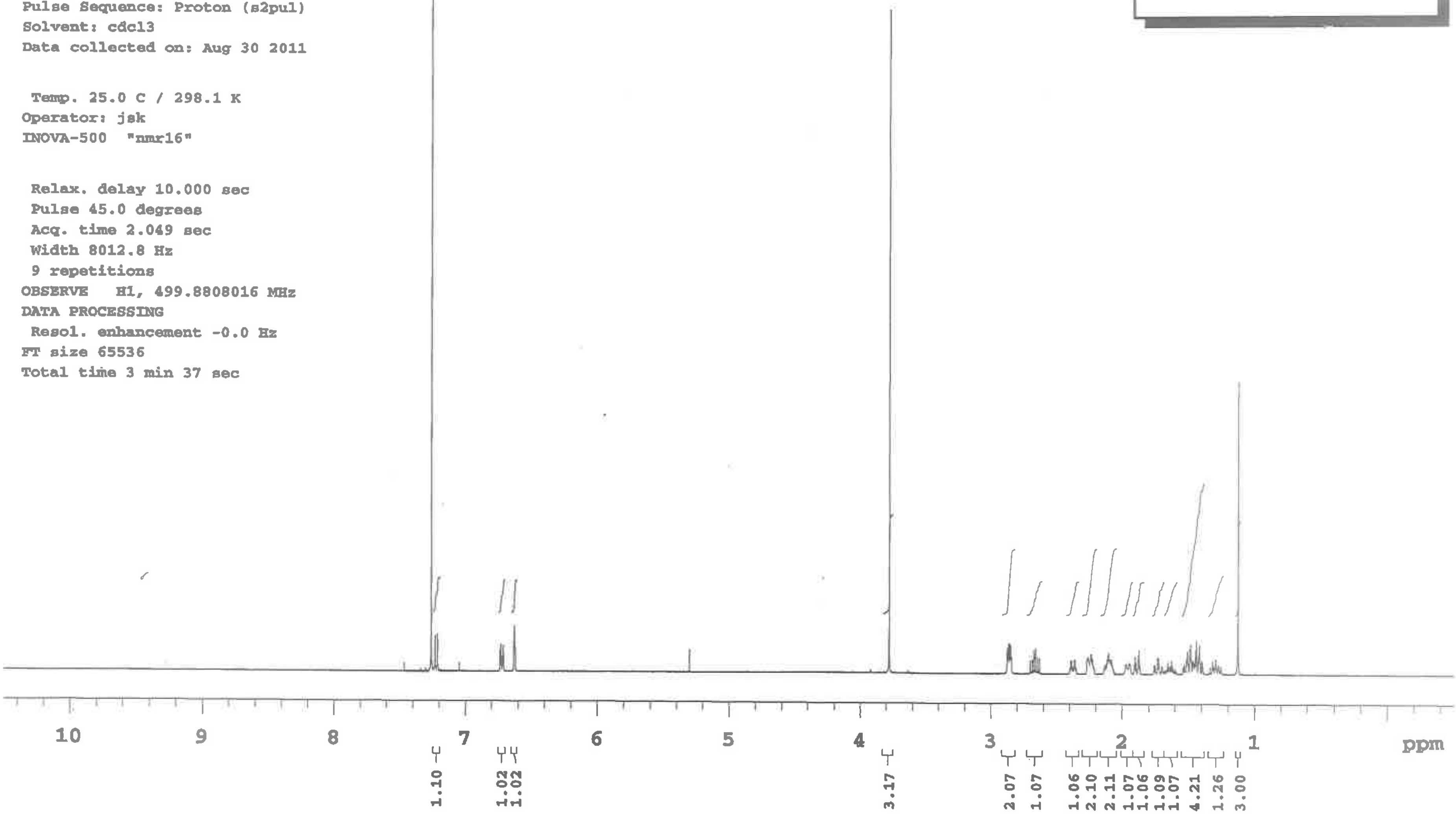

14

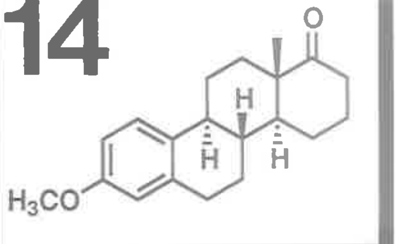

Sample Name:
VR-IV-014Fa-carbon
Archive directory:

Sample directory:

FidFile: VR-IV-014Fa-carbon

Pulse Sequence: Carbon (s2pul)
Solvent: cdcl3
Data collected on: Aug 30 2011

Temp. 25.0 C / 298.1 K
Operator: jsk
INOVA-500 "nmr16"

Relax. delay 1.000 sec
Pulse 45.0 degrees
Acq. time 1.300 sec
Width 30487.8 Hz
124 repetitions
OBSERVE C13, 125.6951105 MHz
DECOUPLE H1, 499.8833015 MHz
Power 40 dB
continuously on
WALTZ-16 modulated
DATA PROCESSING
Line broadening 0.5 Hz
FT size 131072
Total time 39 min

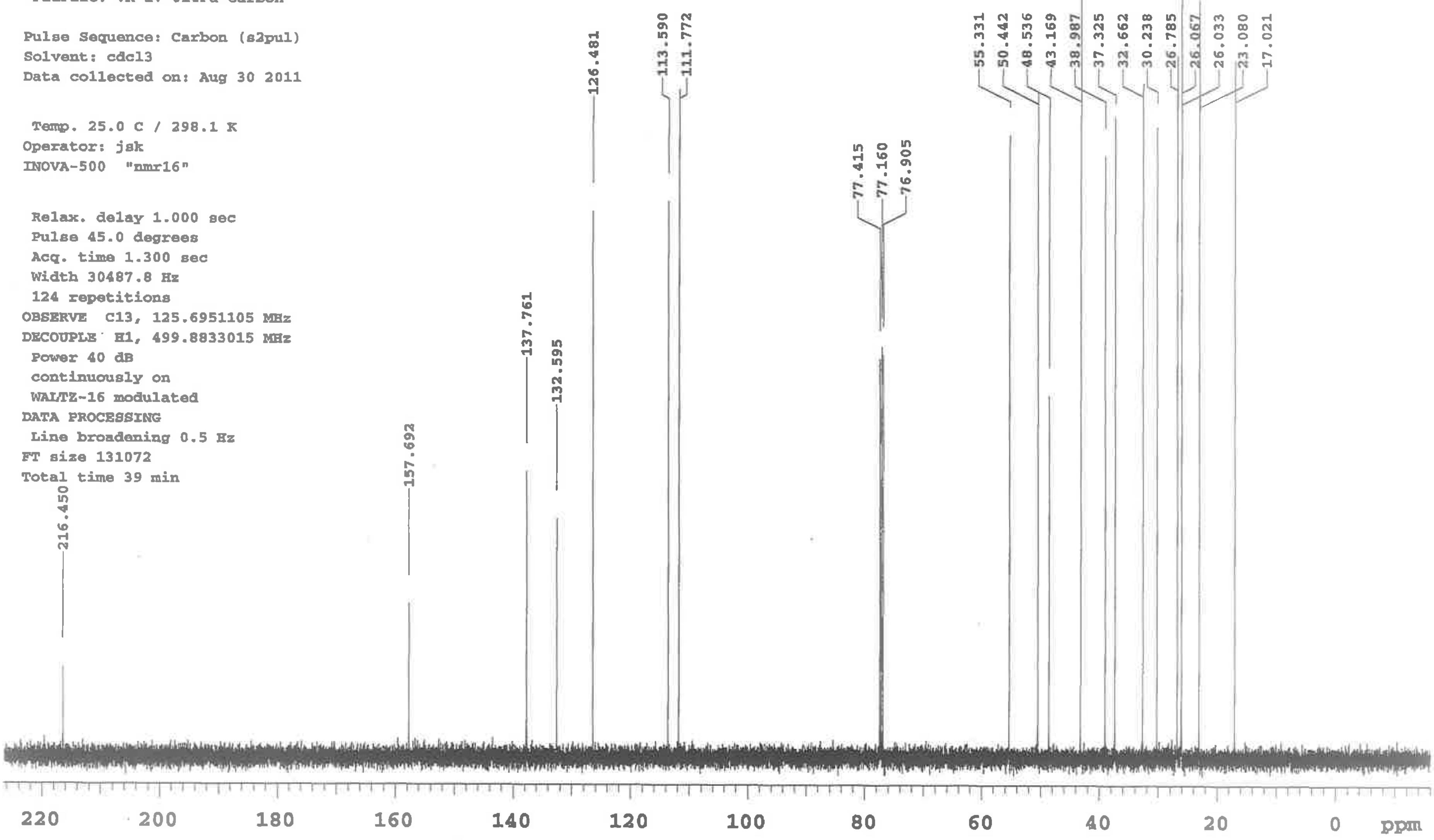

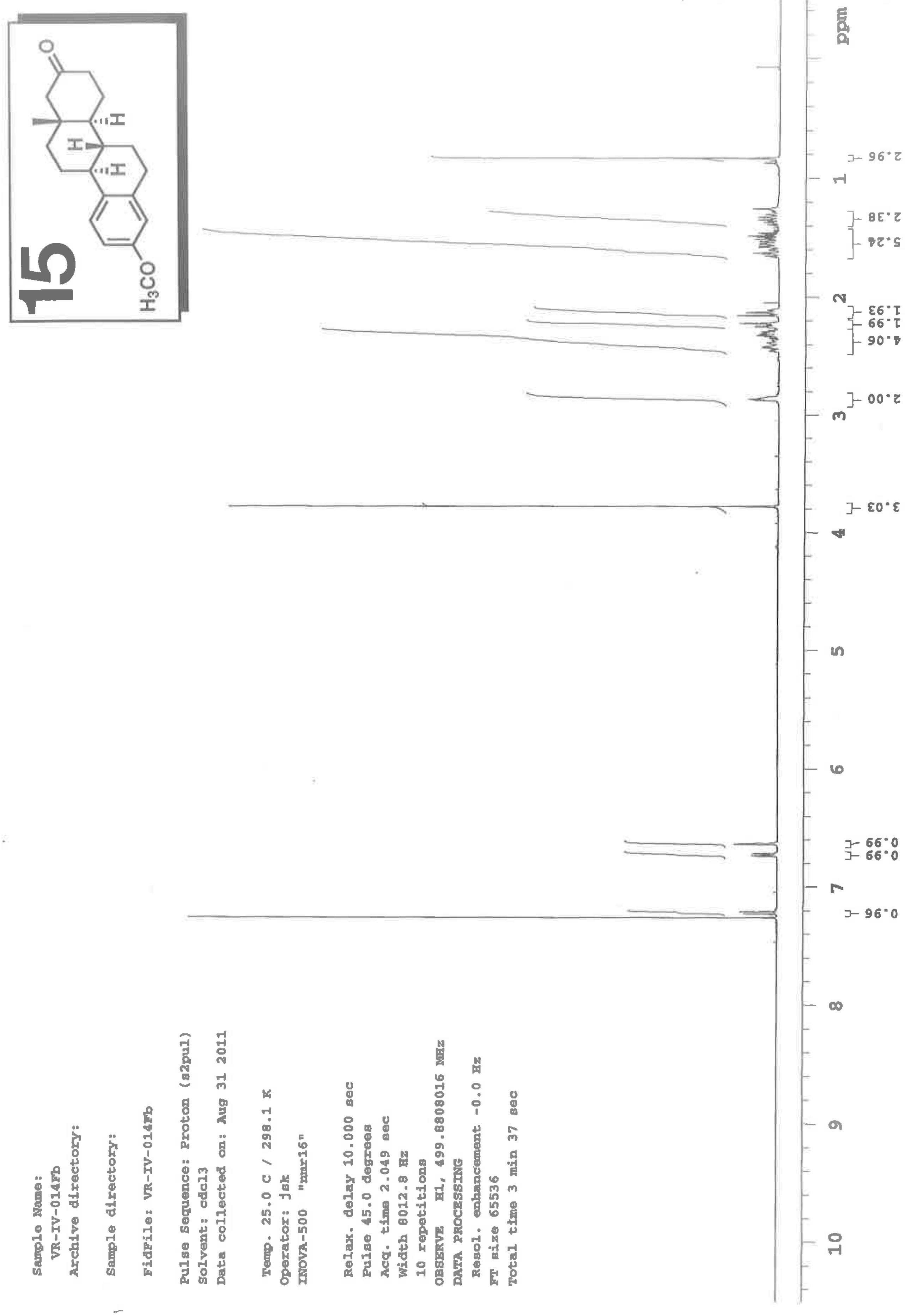
15
H3CO
Sample Name:
VR-IV-014Fb
Archive directory:
Sample directory:
FidFile: VR-IV-014Fb
Pulse Sequence: Proton (s2pul)
Solvent: cdcl3
Data collected on: Aug 31 2011
Temp. 25.0 C / 298.1 K
Operator: jsk
INOVA-500 "nmr16"
Relax. delay 10.000 sec
Pulse 45.0 degrees
Acq. time 2.049 sec
Width 8012.8 Hz
10 repetitions
OBSERVE H1, 499.8808016 MHz
DATA PROCESSING
Resol. enhancement -0.0 Hz
FT size 65536
Total time 3 min 37 sec
ppm
2.96
2.38
5.24
1.93
1.99
4.06
2.00
3.03
0.99
0.99
0.96

15

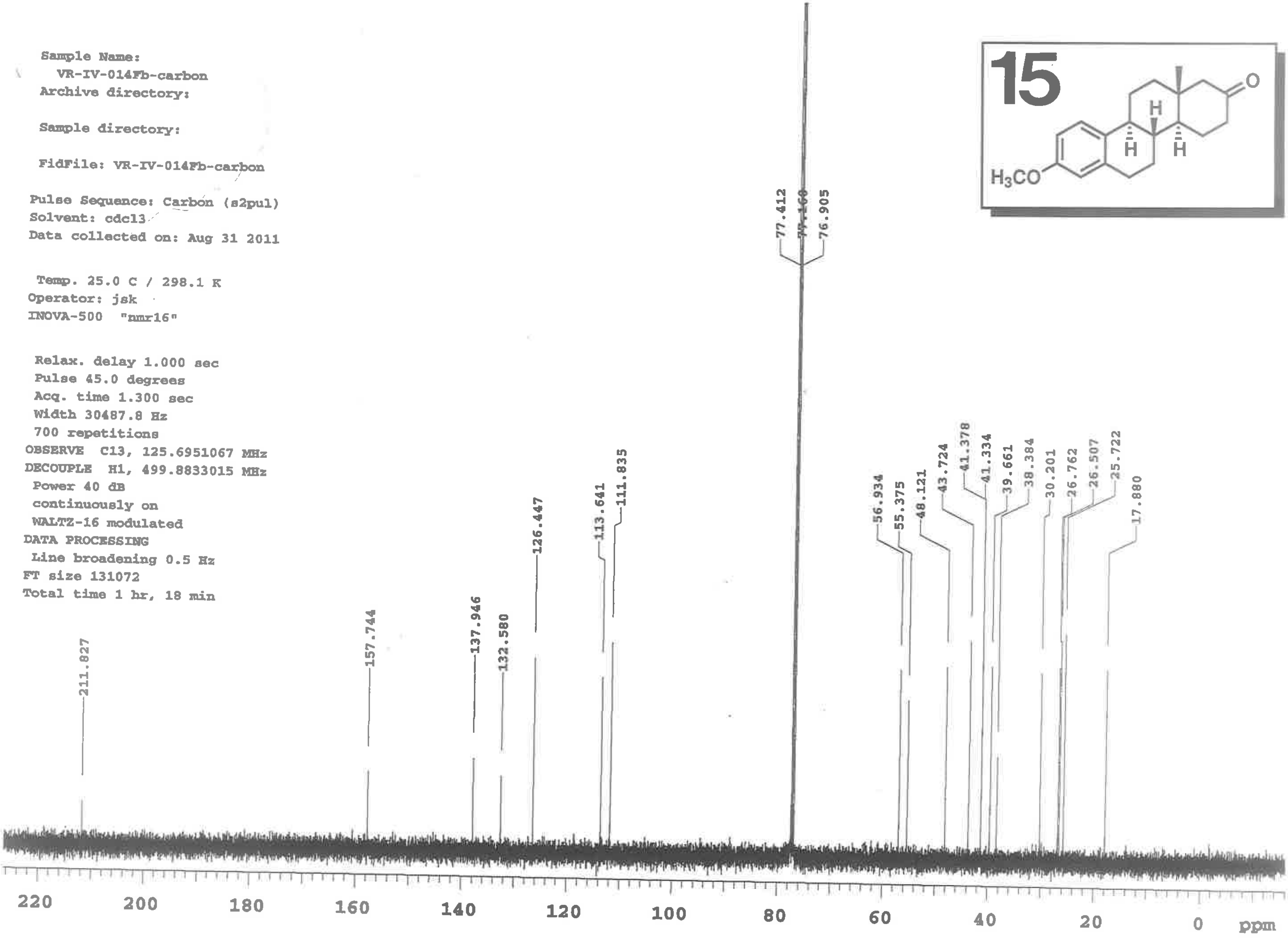
Sample Name:
VR-IV-014Fb-carbon
Archive directory:
Sample directory:
FidFile: VR-IV-014Fb-carbon
Pulse Sequence: Carbon (s2pul)
Solvent: cdcl3
Data collected on: Aug 31 2011
Temp. 25.0 C / 298.1 K
Operator: jsk
INOVA-500 "nmr16"
Relax. delay 1.000 sec
Pulse 45.0 degrees
Acq. time 1.300 sec
Width 30487.8 Hz
700 repetitions
OBSERVE C13, 125.6951067 MHz
DECOUPLE H1, 499.8833015 MHz
Power 40 dB
continuously on
WALTZ-16 modulated
DATA PROCESSING
Line broadening 0.5 Hz
FT size 131072
Total time 1 hr, 18 min
211.827
157.744
137.946
132.580
126.447
113.641
111.835
77.412
76.905
56.934
55.375
48.121
43.724
41.378
41.334
39.661
38.384
30.201
26.762
26.507
25.722
17.880
220
200
180
160
140
120
100
80
60
40
20
0
ppm

Sample Name:
HZK-II-144F
Archive directory:

Sample directory:

FidFile: HZK-II-144F

Pulse Sequence: Proton (s2pul)
Solvent: cdcl3
Data collected on: Jul 19 2011

Operator: jsk
INOVA-500 "nmr16"

Relax. delay 10.000 sec
Pulse 45.0 degrees
Acq. time 3.000 sec
Width 7996.0 Hz
8 repetitions
OBSERVE H1, 499.7720266 MHz
DATA PROCESSING
Resol. enhancement -0.0 Hz
FT size 65536
Total time 3 min 54 sec

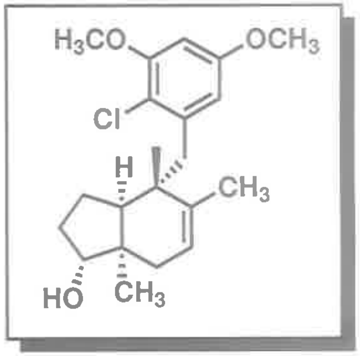

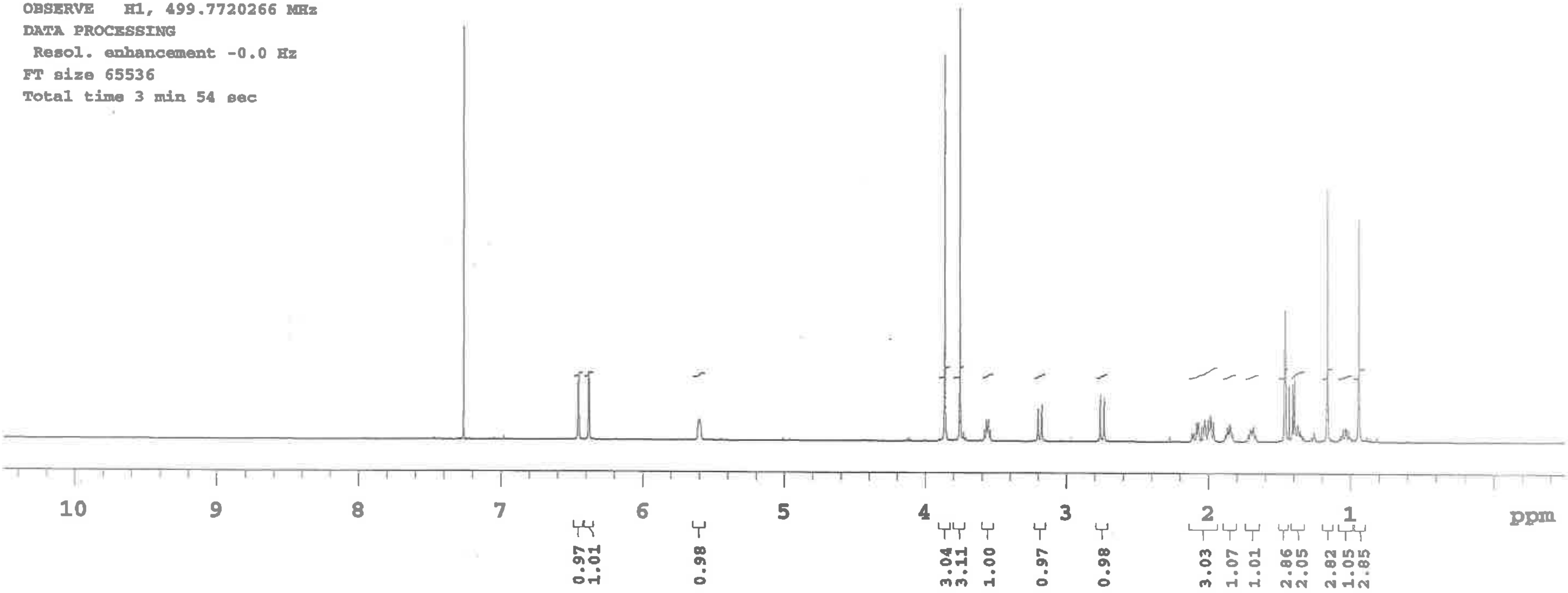

HZK-II-263F2-C

Sample Name:
HZK-II-263F2-C
Archive directory:

Sample directory:

FidFile: HZK-II-263F2-C

Pulse Sequence: Carbon (s2pul)
Solvent: cdcl3
Data collected on: Sep 6 2010

Temp. 25.0 C / 298.1 K
Operator: jsk
INOVA-500 "nmr16"

Relax. delay 1.000 sec
Pulse 45.0 degrees
Acq. time 1.300 sec
Width 30487.8 Hz
500 repetitions
OBSERVE C13, 125.6951067 MHz
DECOUPLE H1, 499.8833015 MHz
Power 40 dB
continuously on
WALTZ-16 modulated
DATA PROCESSING
Line broadening 0.5 Hz
FT size 131072
Total time 19 min

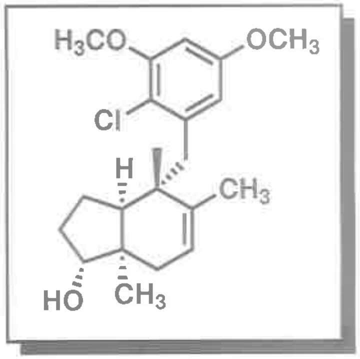

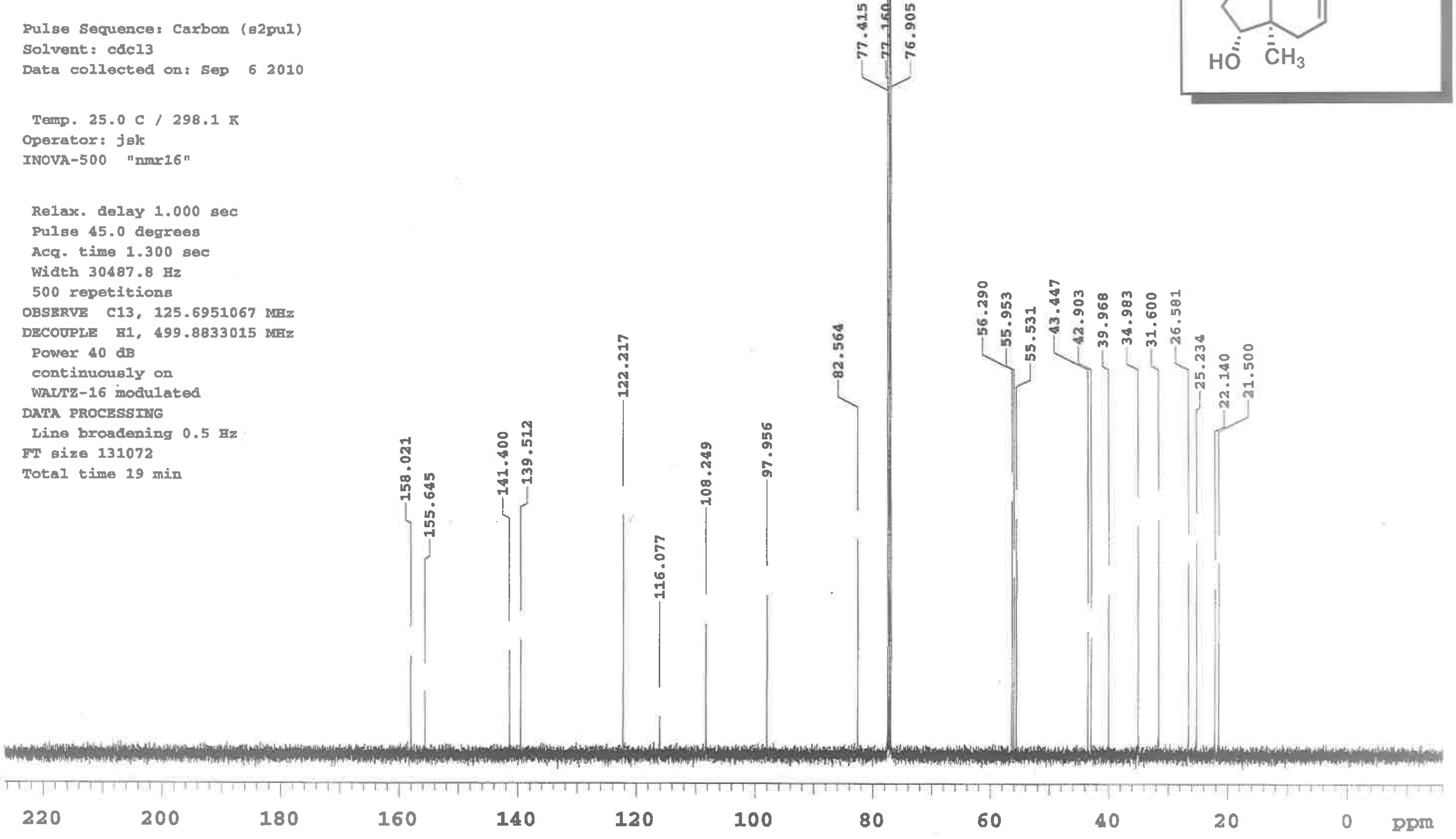

Sample Name:
HZK-II-290FPa
Archive directory:

Sample directory:

FidFile: HZK-II-290FPa

Pulse Sequence: Proton (s2pul)
Solvent: cdcl3
Data collected on: Jul 21 2011

Operator: jsk
INOVA-500 "nmr16"

Relax. delay 10.000 sec
Pulse 45.0 degrees
Acq. time 3.000 sec
Width 7996.0 Hz
12 repetitions
OBSERVE H1, 499.7720603 MHz
DATA PROCESSING
Resol. enhancement -0.0 Hz
FT size 65536
Total time 3 min 54 sec

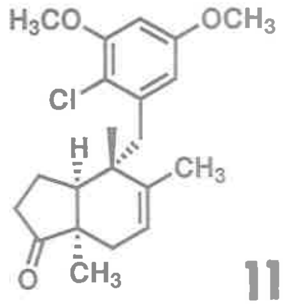

11

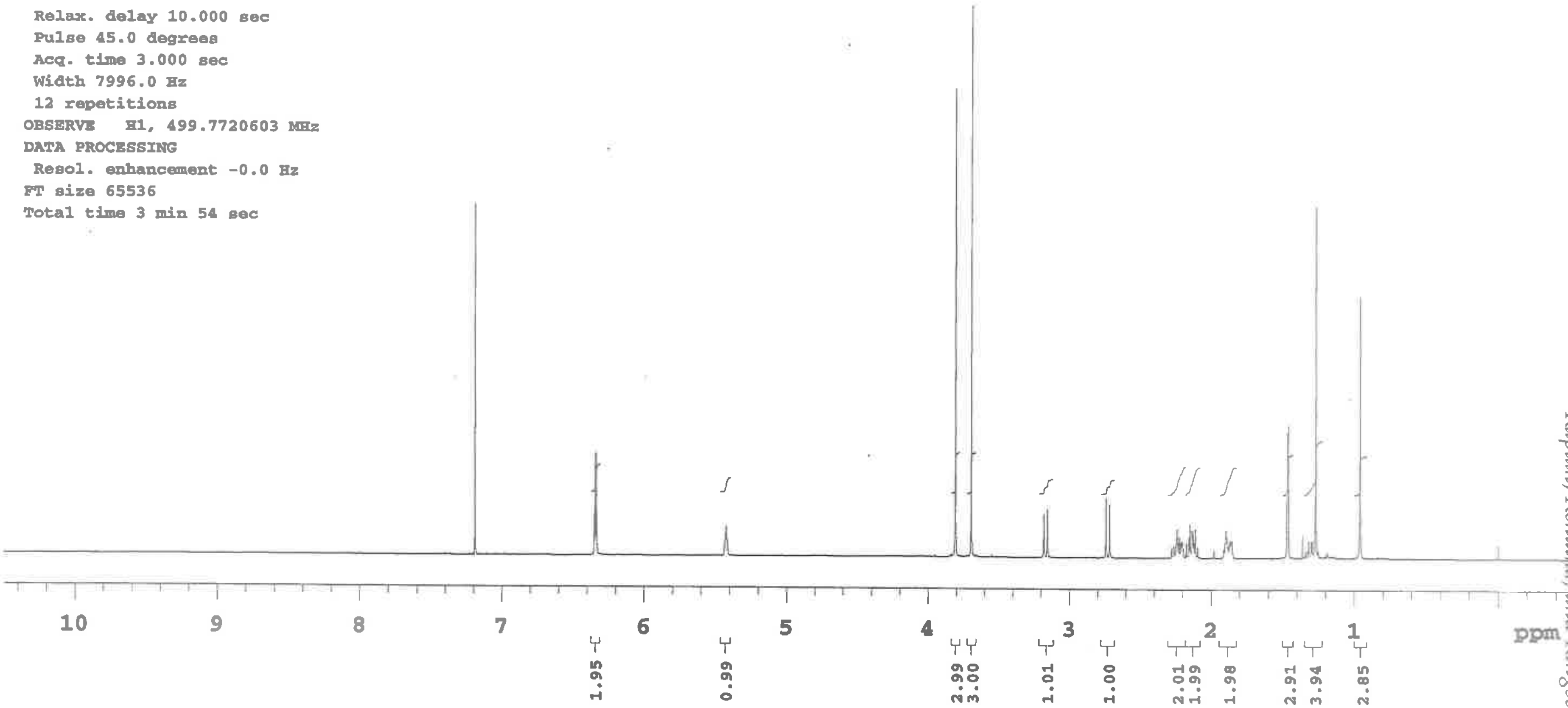

HZK-II-267F-C

Sample Name:
HZK-II-267F-C
Archive directory:

Sample directory:

FidFile: HZK-II-267F-C_II_267_01

Pulse Sequence: Carbon (s2pul)
Solvent: cdcl3
Data collected on: Sep 7 2010

Temp. 25.0 C / 298.1 K
Sample #27, Operator: kaplanhi
INOVA-500 "nmr16"

Relax. delay 1.000 sec
Pulse 45.0 degrees
Acq. time 1.300 sec
Width 24509.8 Hz
500 repetitions
OBSERVE C13, 100.5212941 MHz
DECOUPLE H1, 399.7682756 MHz
Power 40 dB
continuously on
WALTZ-16 modulated
DATA PROCESSING
Line broadening 0.5 Hz
FT size 65536
Total time 19 min

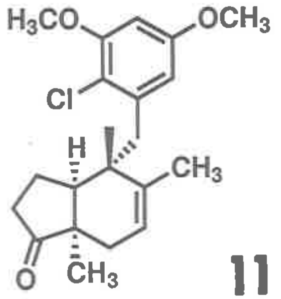

11

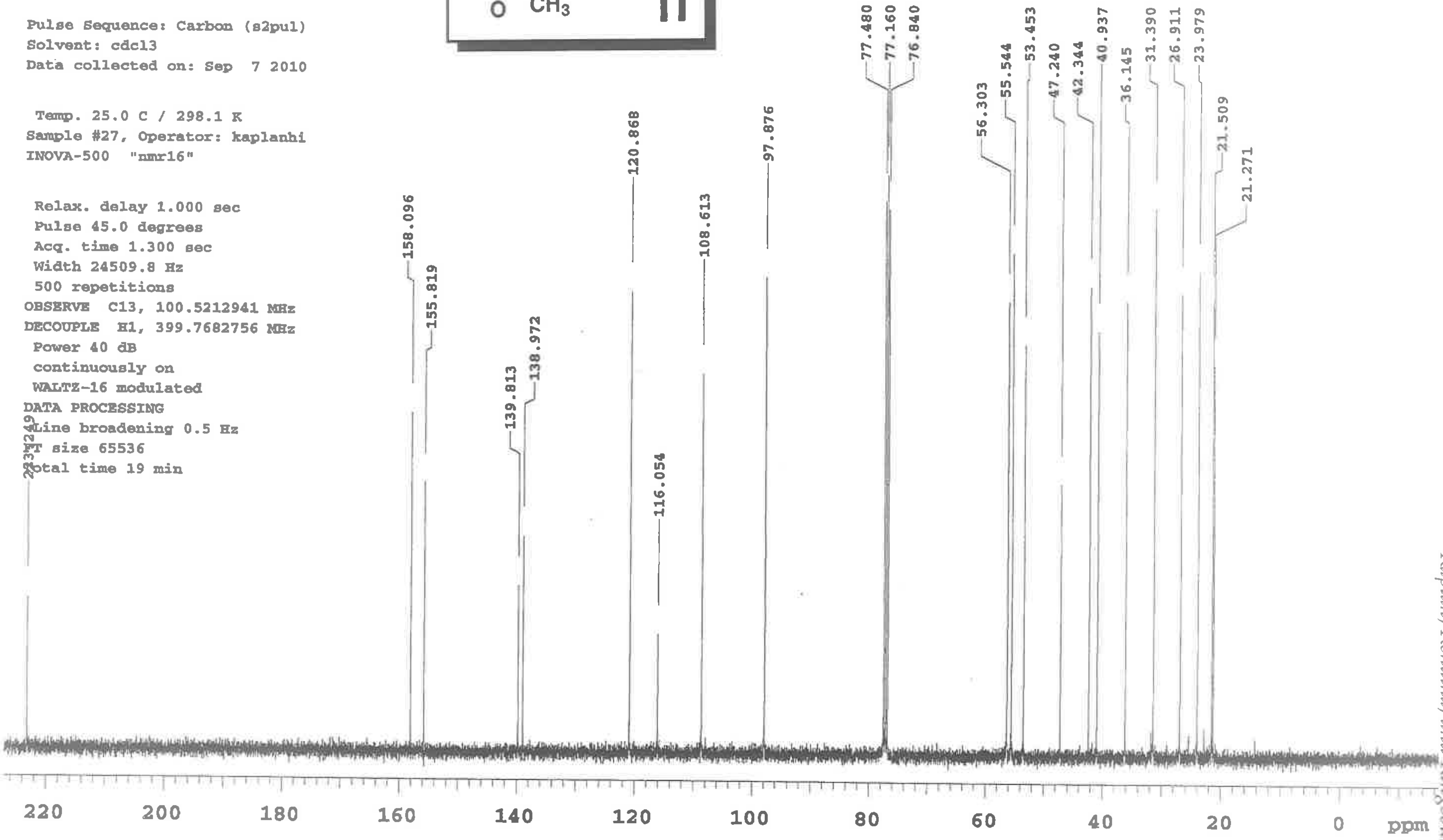

Sample Name:
 HZK-II-253F
Archive directory:

Sample directory:

FidFile: Proton

Pulse Sequence: Proton (s2pul)
Solvent: cdcl3
Data collected on: Jul 18 2011

Operator: jsk
INOVA-500 "nmr16"

Relax. delay 5.000 sec
Pulse 45.0 degrees
Acq. time 3.000 sec
Width 7996.0 Hz
8 repetitions
OBSERVE H1, 499.7720264 MHz
DATA PROCESSING
Resol. enhancement -0.0 Hz
FT size 65536
Total time 1 min 20 sec

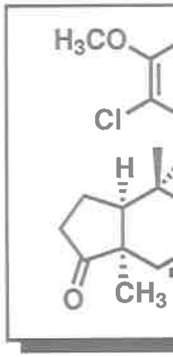

12B

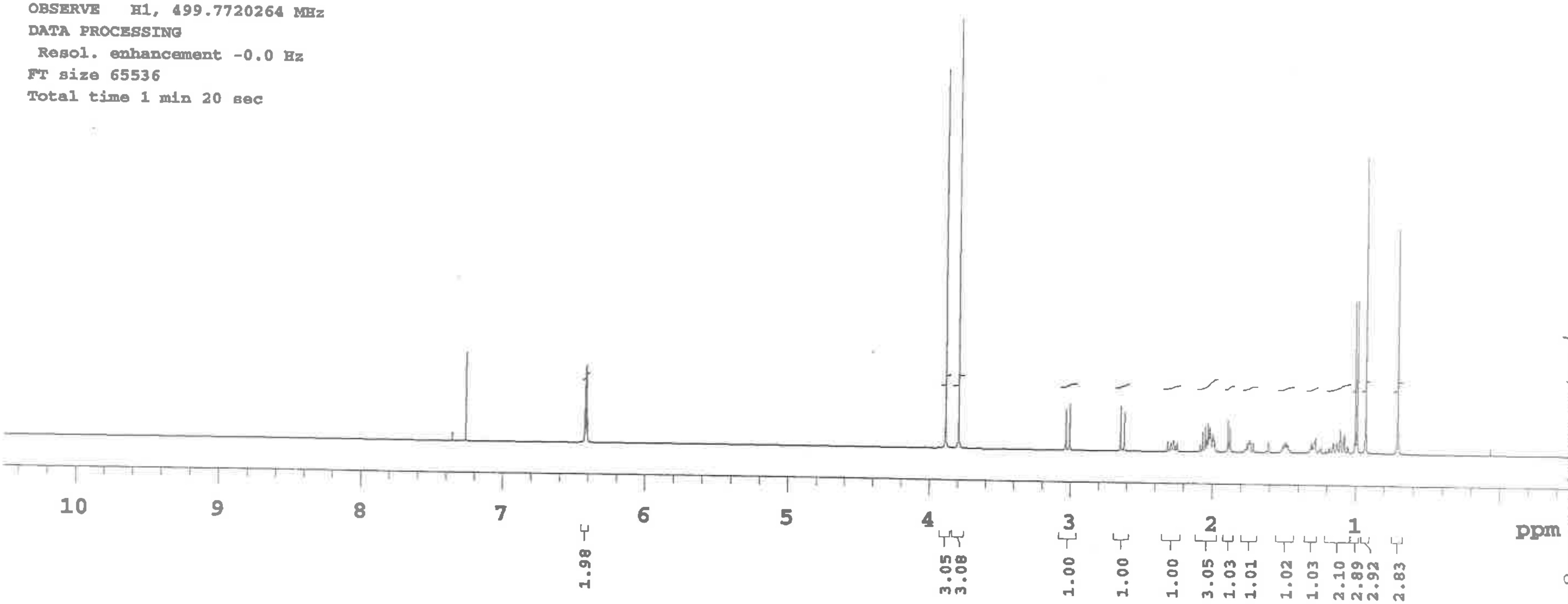

Sample Name:
HZK-II-253F-carbon
Archive directory:

Sample directory:

FidFile: HZK-II-253F-carbon

Pulse Sequence: Carbon (s2pul)
Solvent: cdcl3
Data collected on: Jul 18 2011

Operator: jsk
INOVA-500 "nmr16"

Relax. delay 1.000 sec
Pulse 45.0 degrees
Acq. time 1.300 sec
Width 30165.9 Hz
20000 repetitions
OBSERVE C13, 125.6677586 MHz
DECOUPLE H1, 499.7745112 MHz
Power 45 dB
continuously on
WALTZ-16 modulated
DATA PROCESSING
Line broadening 0.5 Hz
FT size 131072
Total time 12 hr, 46 min

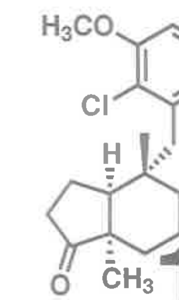

12B

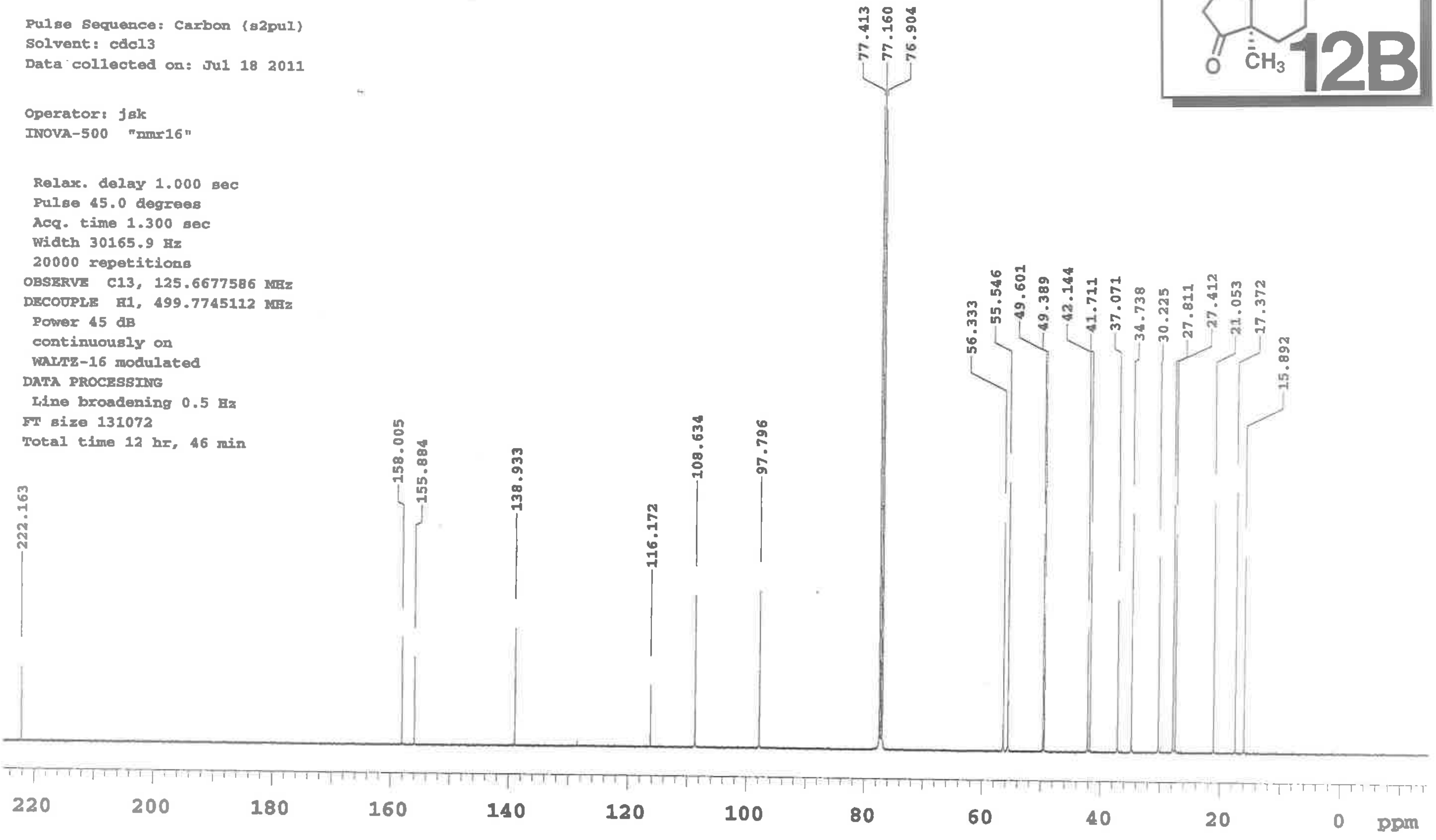

Sample Name:
HZK-III-043Pb
Archive directory:

Sample directory:

FidFile: HZK-III-043Pb

Pulse Sequence: Proton (s2pul)
Solvent: cdcl3
Data collected on: Jul 19 2011

Operator: jsk
INOVA-500 "nmr16"

Relax. delay 10.000 sec
Pulse 45.0 degrees
Acq. time 3.000 sec
Width 7996.0 Hz
8 repetitions
OBSERVE H1, 499.7720264 MHz
DATA PROCESSING
Resol. enhancement -0.0 Hz
FT size 65536
Total time 2 min 10 sec

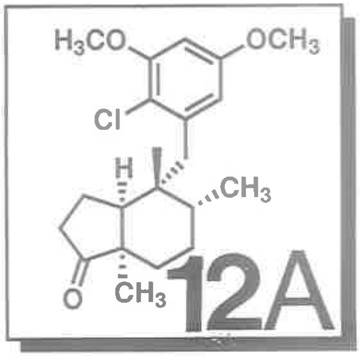

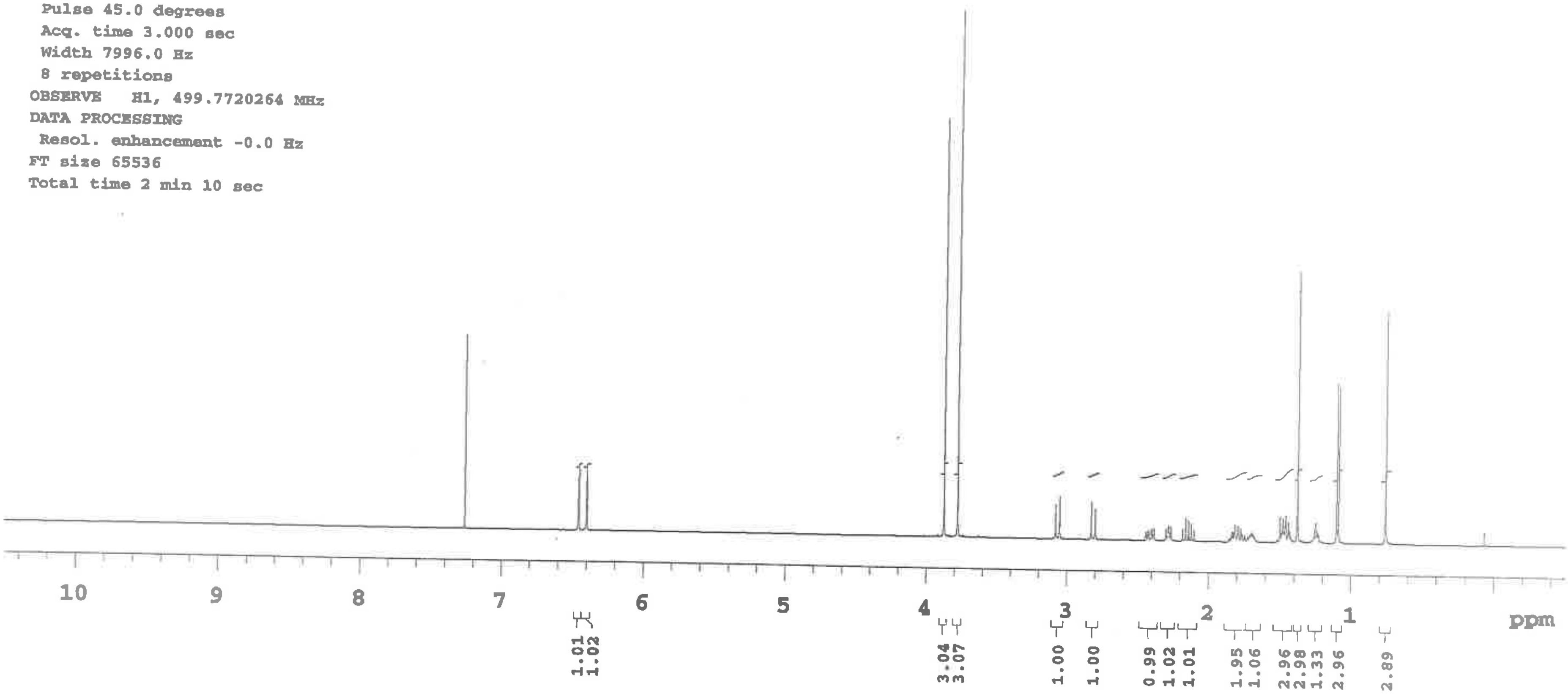

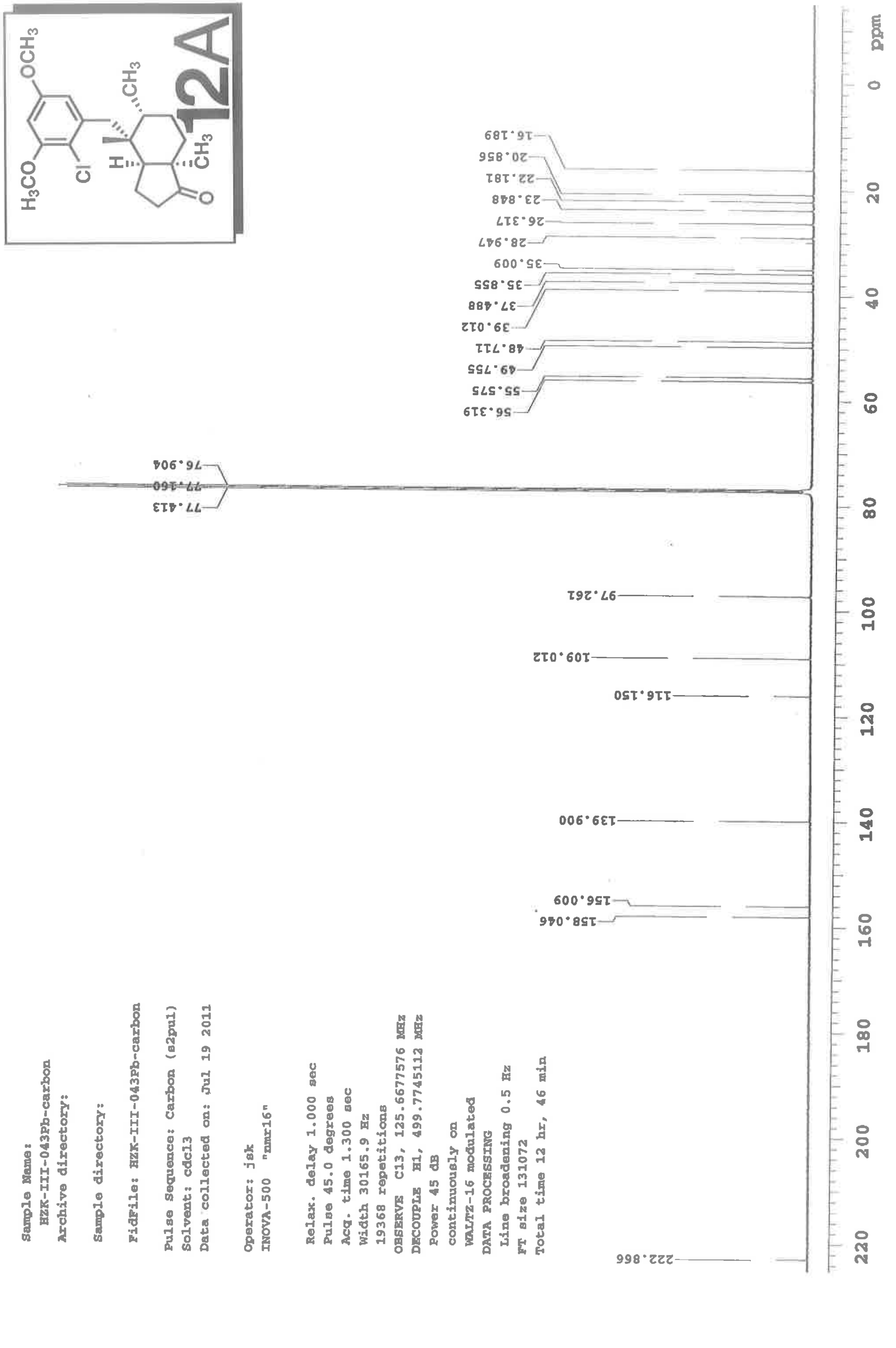
12A
Sample Name:
HZK-III-043Pb-carbon
Archive directory:
Sample directory:
FidFile: HZK-III-043Pb-carbon
Pulse Sequence: Carbon (s2pul)
Solvent: cdcl3
Data collected on: Jul 19 2011
Operator: jsk
INOVA-500 "nmr16"
Relax. delay 1.000 sec
Pulse 45.0 degrees
Acq. time 1.300 sec
Width 30165.9 Hz
19368 repetitions
OBSERVE C13, 125.6677576 MHz
DECOUPLE H1, 499.7745112 MHz
Power 45 dB
continuously on
WALTZ-16 modulated
DATA PROCESSING
Line broadening 0.5 Hz
FT size 131072
Total time 12 hr, 46 min
222.866
158.046
156.009
139.900
116.150
109.012
97.261
77.413
77.160
76.904
56.319
55.575
49.755
48.711
39.012
37.488
35.855
35.009
28.947
26.317
23.848
22.181
20.856
16.189
ppm

HZK-III-118P

Sample Name:
HZK-III-118P
Archive directory:

Sample directory:

FidFile: HZK-III-118P

Pulse Sequence: Proton (s2pul)
Solvent: cdcl3
Data collected on: Jul 22 2011

Operator: jsk
INOVA-500 "nmr16"

Relax. delay 10.000 sec
Pulse 45.0 degrees
Acq. time 2.049 sec
Width 8012.8 Hz
8 repetitions
OBSERVE H1, 499.8808016 MHz
DATA PROCESSING
Resol. enhancement -0.0 Hz
FT size 65536
Total time 2 min 0 sec

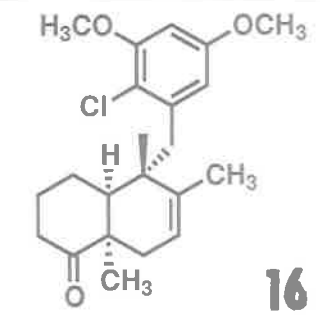

16

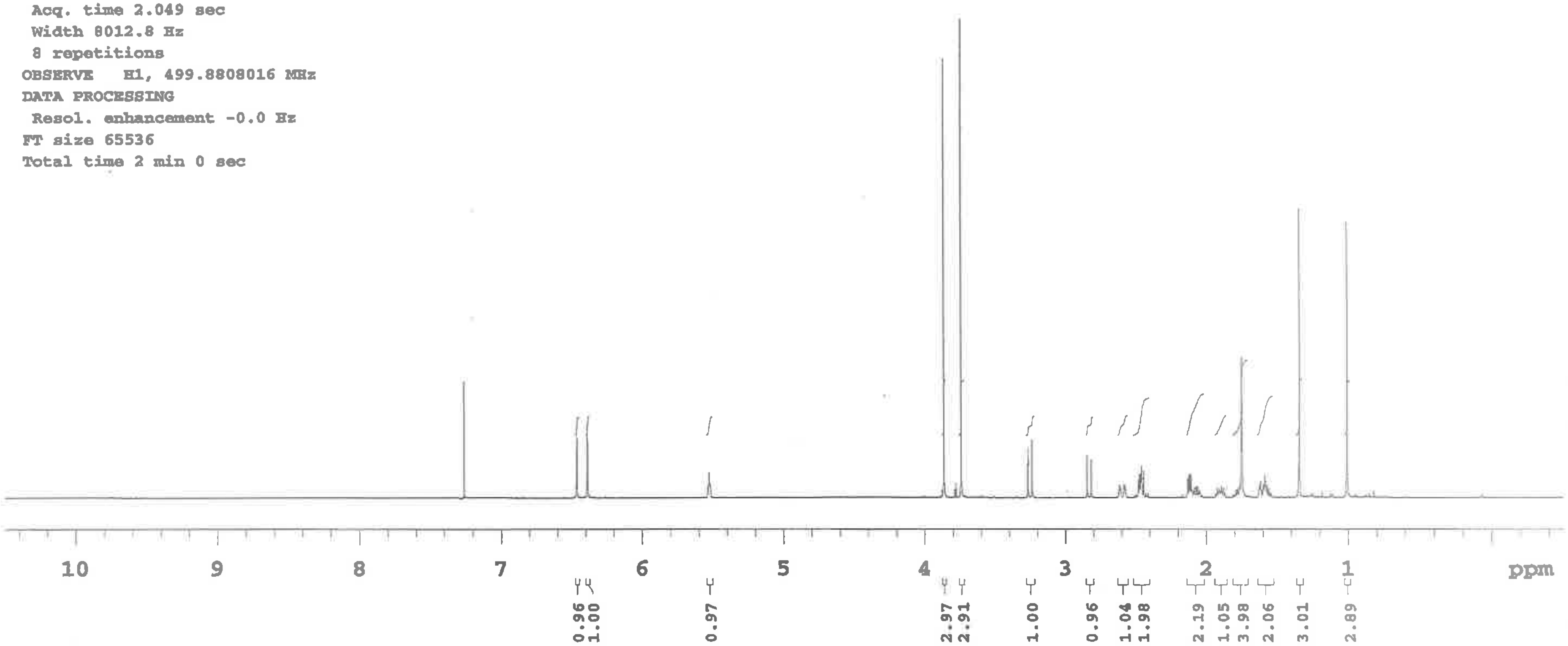

HZK-III-118P-carbon

Sample: HZK-III-118P-carbon
File: exp

Pulse Sequence: s2pul

Solvent: cdcl3
Ambient temperature
Operator: jsk
INOVA-500 "nmr11"

Relax. delay 1.000 sec
Pulse 45.0 degrees
Acq. time 1.300 sec
Width 30165.9 Hz
15000 repetitions
OBSERVE C13, 125.6677586 MHz
DECOUPLE H1, 499.7745112 MHz
Power 45 dB
continuously on
WALTZ-16 modulated
DATA PROCESSING
Line broadening 0.5 Hz
FT size 131072
Total time 9 hr, 37 min, 21 sec

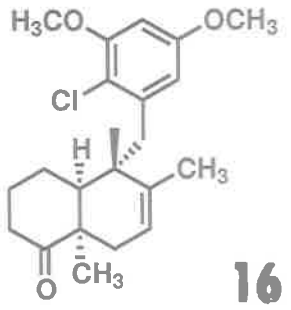

16

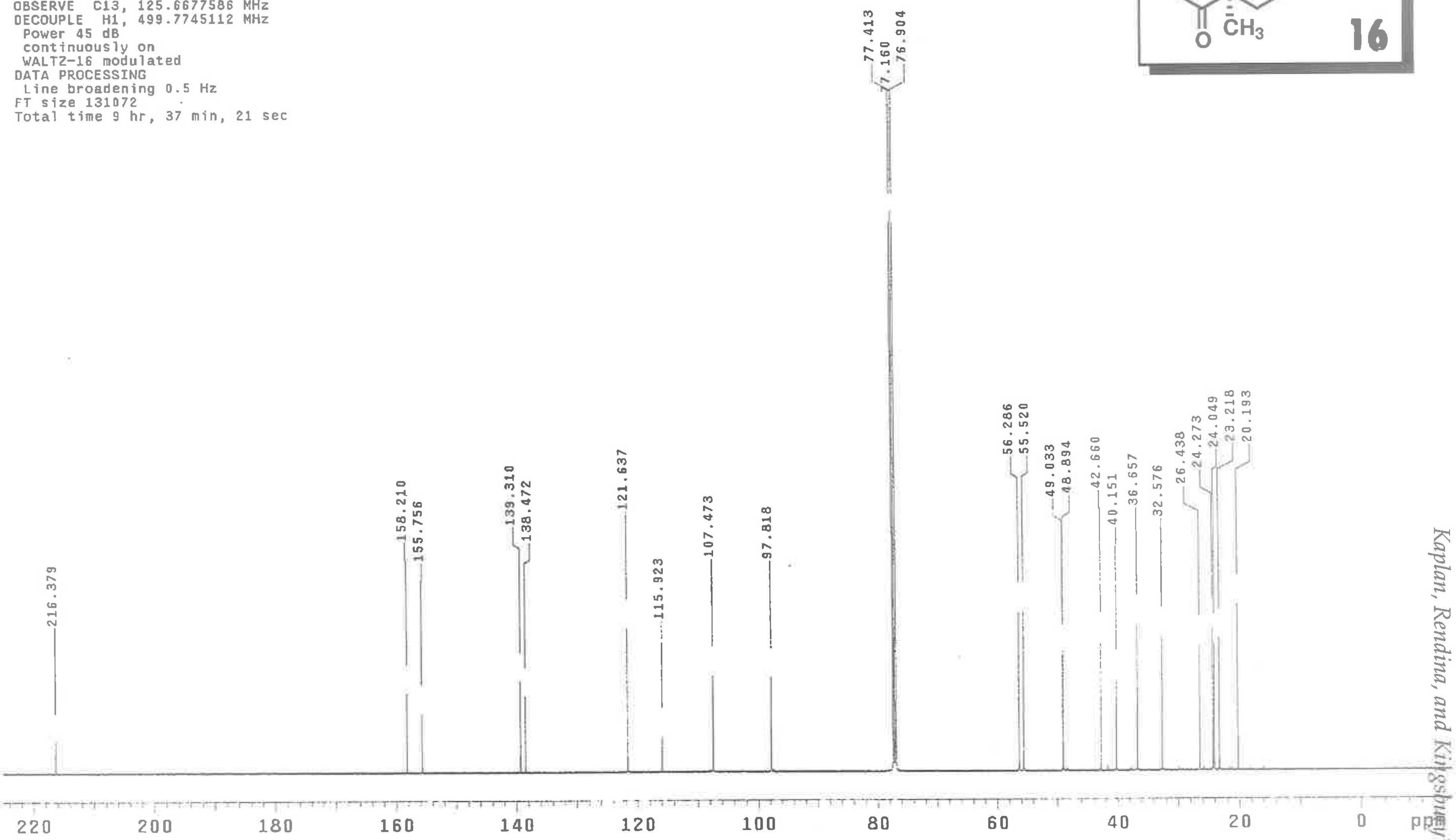

Sample Name:
HZK-III-213F
Archive directory:

Sample directory:

FidFile: Proton

Pulse Sequence: Proton (s2pul)
Solvent: cdcl3
Data collected on: Jun 2 2011

Temp. 25.0 C / 298.1 K
Operator: jsk
INOVA-500 "nmr16"

Relax. delay 10.000 sec
Pulse 45.0 degrees
Acq. time 2.049 sec
Width 8012.8 Hz
7 repetitions
OBSERVE H1, 499.8807991 MHz
DATA PROCESSING
Resol. enhancement -0.0 Hz
FT size 65536
Total time 3 min 37 sec

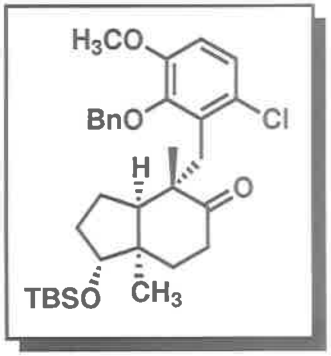

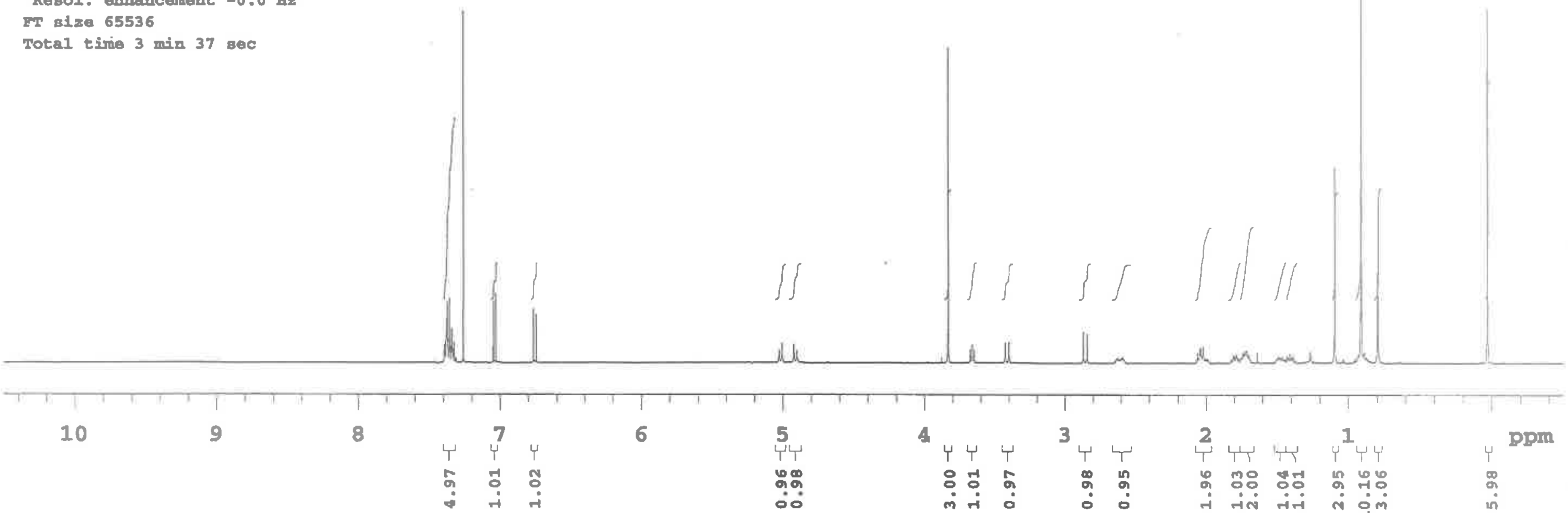

Sample Name:
HZK-III-213F-carbon
Archive directory:

Sample directory:

FidFile: HZK-III-213F-carbon

Pulse Sequence: Carbon (s2pul)
Solvent: cdcl3
Data collected on: Jun 3 2011

Temp. 25.0 C / 298.1 K
Operator: jsk
INOVA-500 "nmr16"

Relax. delay 1.000 sec
Pulse 45.0 degrees
Acq. time 1.300 sec
Width 30487.8 Hz
180 repetitions
OBSERVE C13, 125.6951086 MHz
DECOUPLE H1, 499.8833015 MHz
Power 40 dB
continuously on
WALTZ-16 modulated
DATA PROCESSING
Line broadening 0.5 Hz
FT size 131072
Total time 19 min

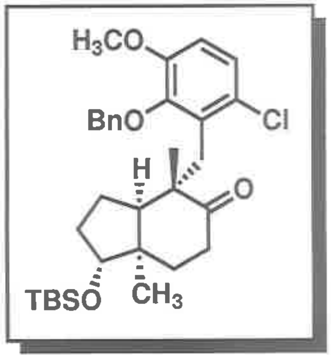

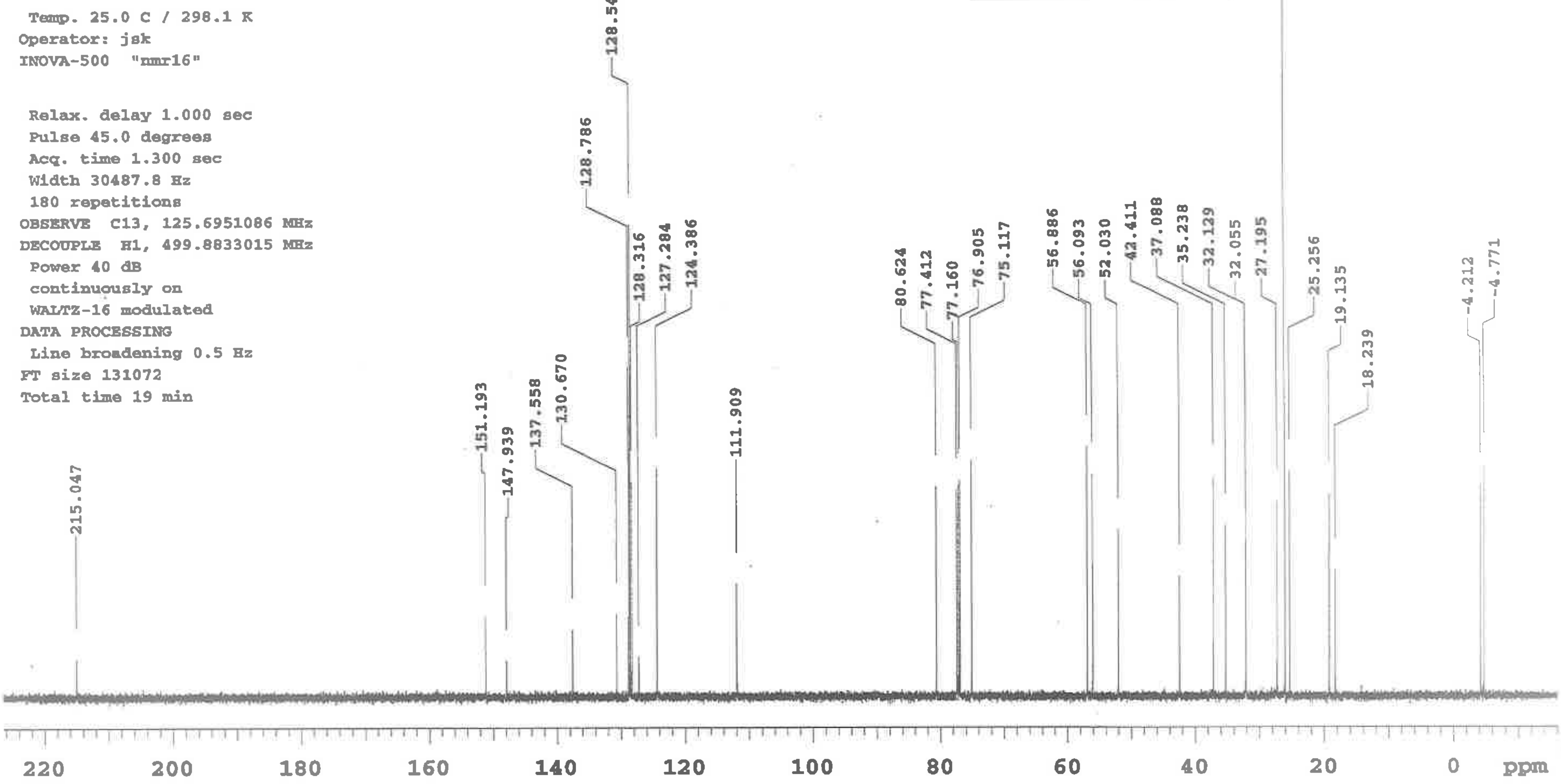

Sample Name:
HZK-III-233F
Archive directory:

Sample directory:

FidFile: HZK-III-233F

Pulse Sequence: Proton (s2pul)
Solvent: cdcl3
Data collected on: Jun 8 2011

Temp. 25.0 C / 298.1 K
Operator: jsk
INOVA-500 "nmr16"

Relax. delay 10.000 sec
Pulse 45.0 degrees
Acq. time 2.049 sec
Width 8012.8 Hz
8 repetitions
OBSERVE H1, 499.8807996 MHz
DATA PROCESSING
Resol. enhancement -0.0 Hz
FT size 65536
Total time 2 min 0 sec

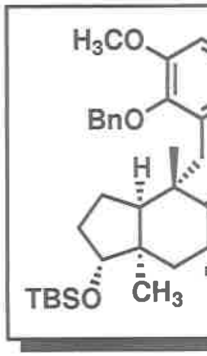

17

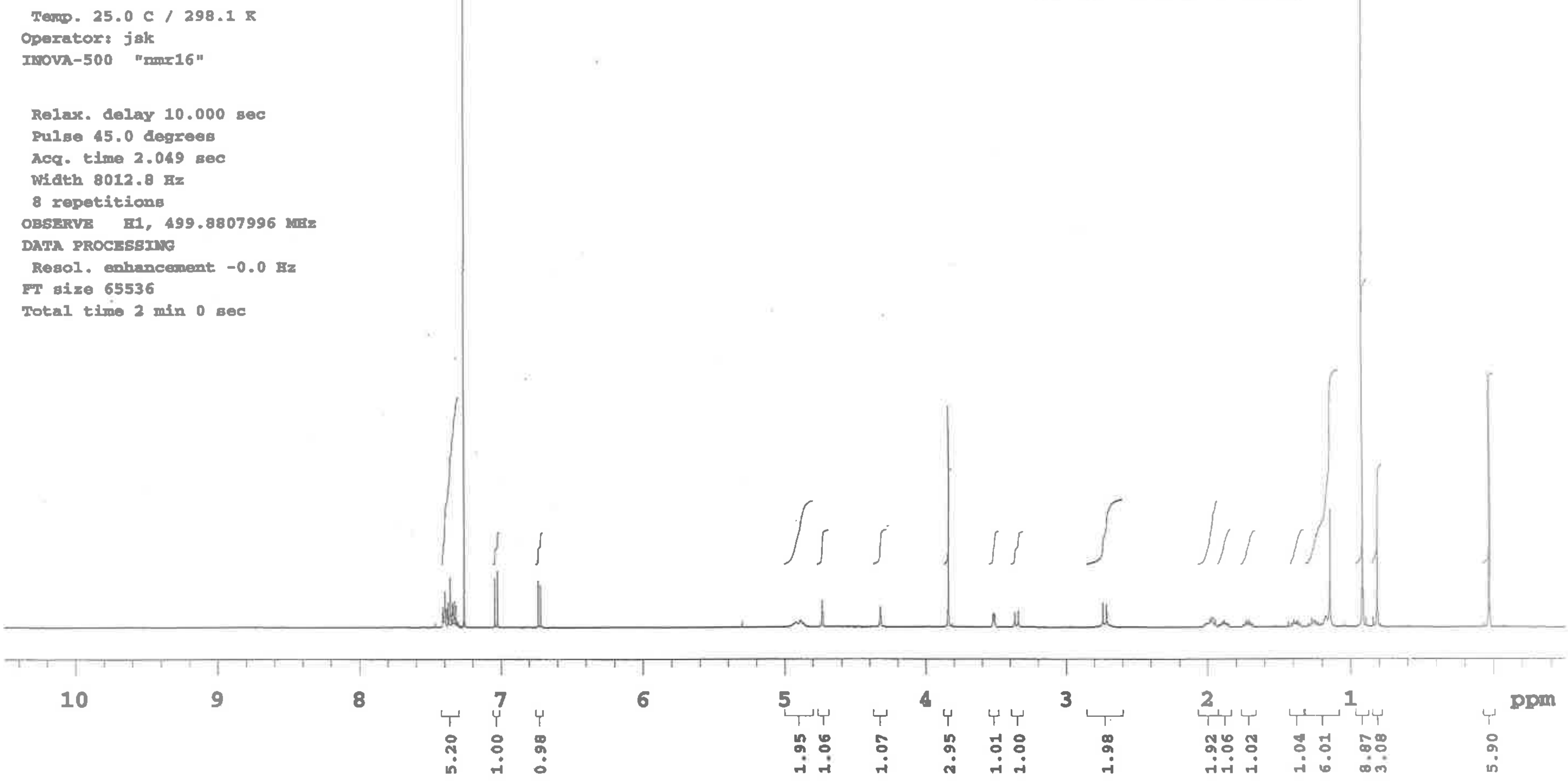

Sample Name:
HZK-III-233F-carbon
Archive directory:

Sample directory:

FidFile: HZK-III-233F-carbon

Pulse Sequence: Carbon (s2pul)
Solvent: cdcl3
Data collected on: Jun 8 2011

Temp. 25.0 C / 298.1 K
Operator: jsk
INOVA-500 "nmr16"

Relax. delay 1.000 sec
Pulse 45.0 degrees
Acq. time 1.300 sec
Width 30165.9 Hz
292 repetitions
OBSERVE C13, 125.6677590 MHz
DECOUPLE H1, 499.7745112 MHz
Power 45 dB
continuously on
WALTZ-16 modulated
DATA PROCESSING
Line broadening 0.5 Hz
FT size 131072
Total time 19 min

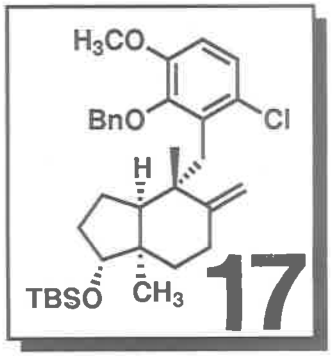

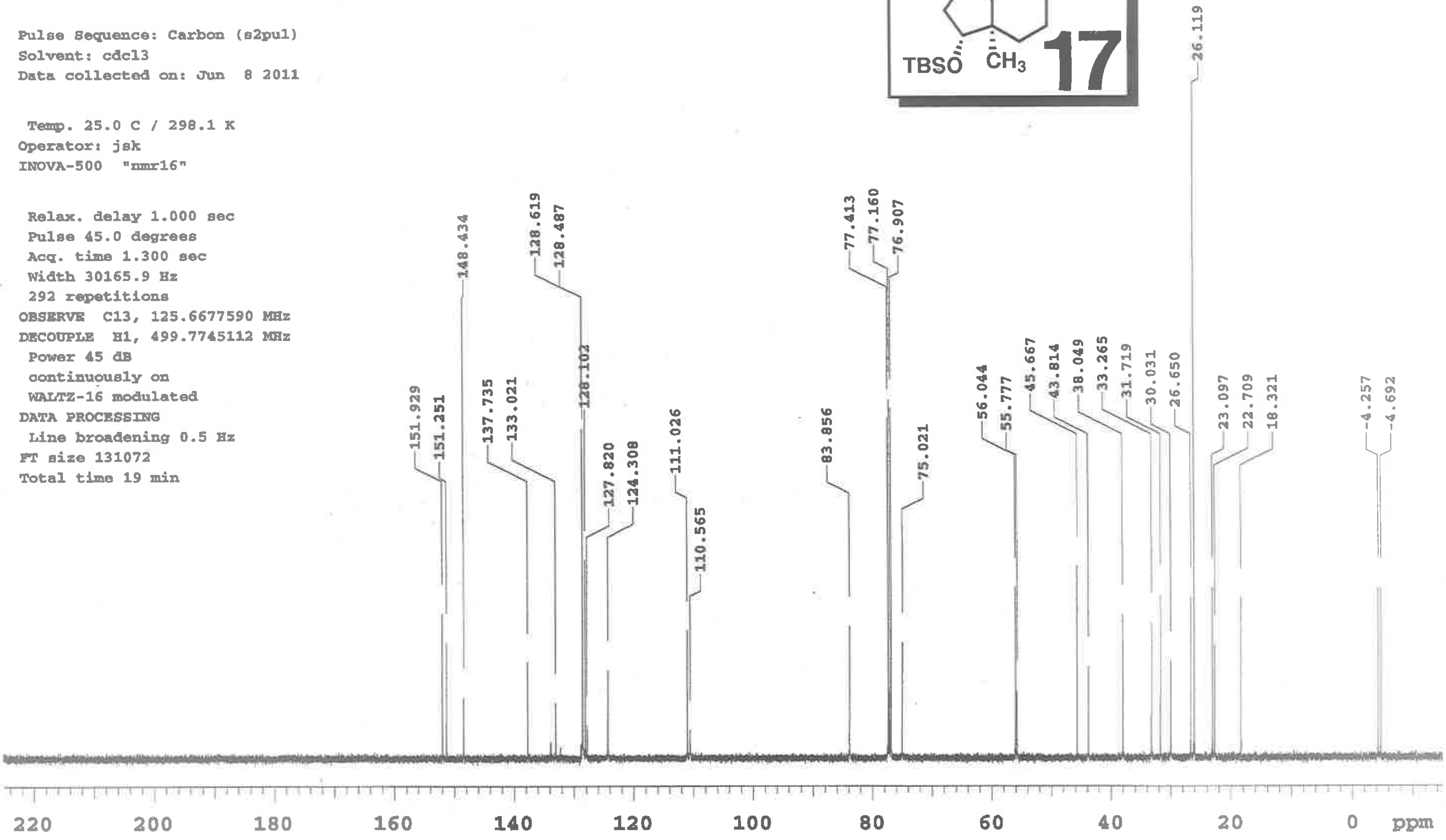

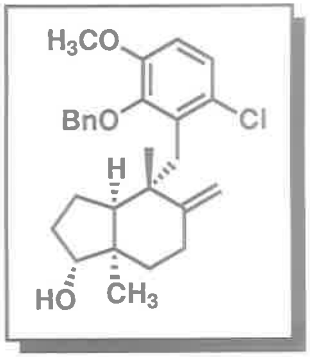

Sample Name:
VR-III-279f
Archive directory:

Sample directory:

FidFile: VR-III-279f

Pulse Sequence: Proton (s2pul)
Solvent: cdcl3
Data collected on: Jun 22 2011

Temp. 25.0 C / 298.1 K
Operator: jsk
INOVA-500 "nmr16"

Relax. delay 10.000 sec
Pulse 45.0 degrees
Acq. time 3.000 sec
Width 7996.0 Hz
16 repetitions
OBSERVE H1, 499.7720271 MHz
DATA PROCESSING
Resol. enhancement -0.0 Hz
FT size 65536
Total time 3 min 54 sec

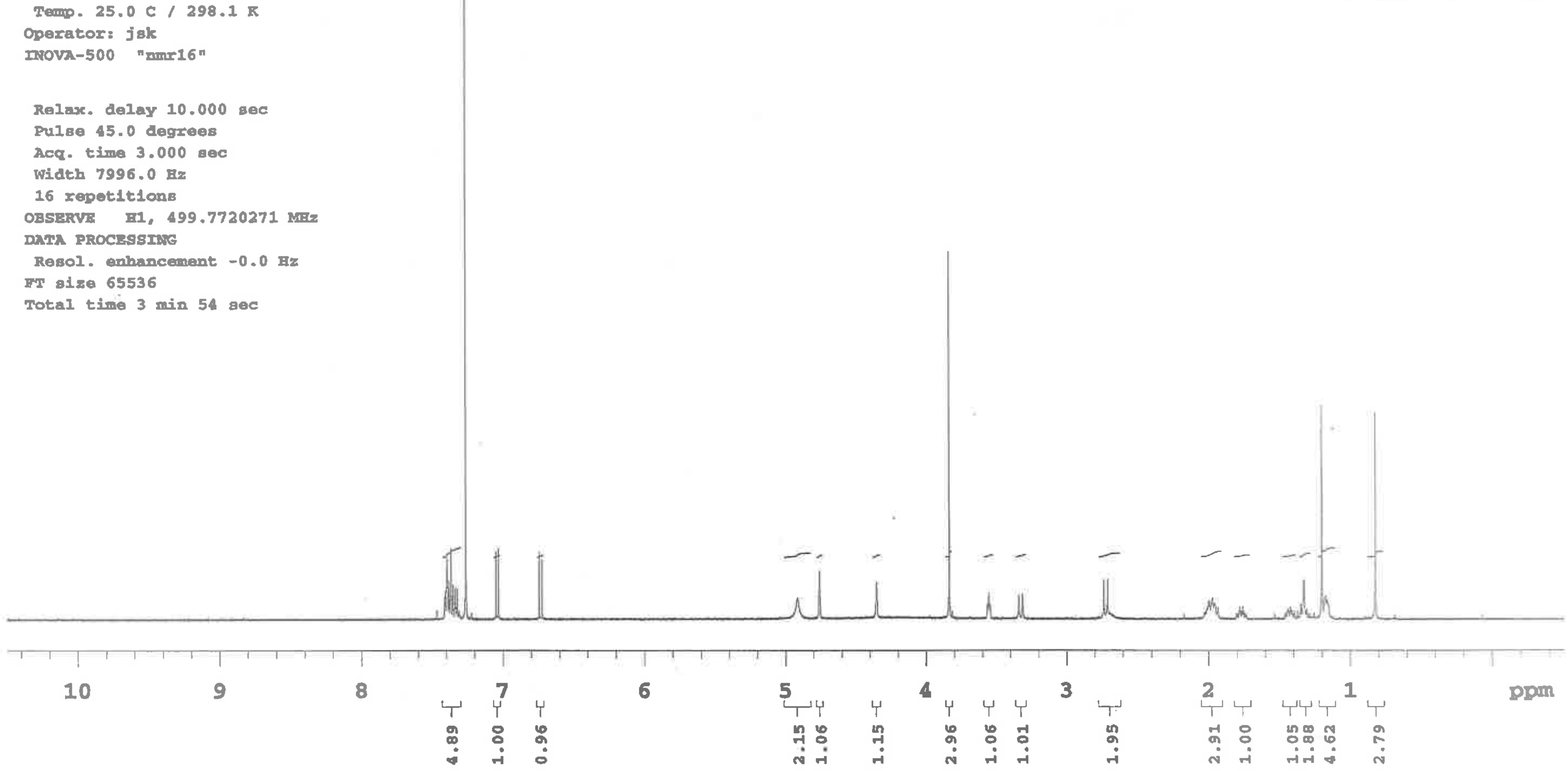

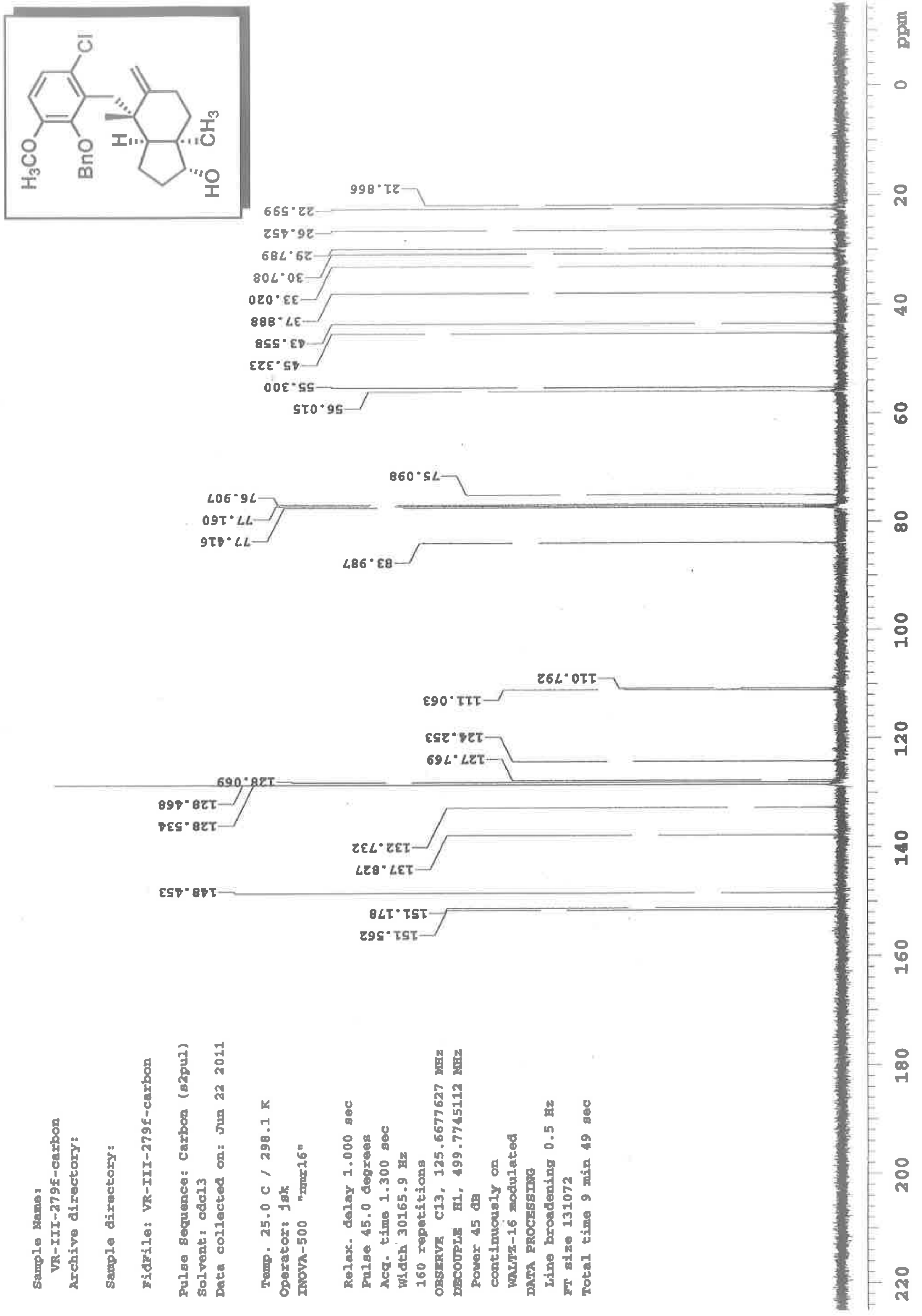
Cl
H3CO
BnO
H
CH3
HO
Sample Name:
VR-III-279f-carbon
Archive directory:
Sample directory:
FidFile: VR-III-279f-carbon
Pulse Sequence: Carbon (s2pul)
Solvent: cdcl3
Data collected on: Jun 22 2011
Temp. 25.0 C / 298.1 K
Operator: jsk
INOVA-500 "nmr16"
Relax. delay 1.000 sec
Pulse 45.0 degrees
Acq. time 1.300 sec
Width 30165.9 Hz
160 repetitions
OBSERVE C13, 125.6677627 MHz
DECOUPLE H1, 499.7745112 MHz
Power 45 dB
continuously on
WALTZ-16 modulated
DATA PROCESSING
Line broadening 0.5 Hz
FT size 131072
Total time 9 min 49 sec
21.866
22.599
26.452
29.789
30.708
33.020
37.888
43.558
45.323
55.300
56.015
75.098
76.907
77.160
77.416
83.987
110.792
111.063
124.253
127.769
128.069
128.468
128.534
132.732
137.827
148.453
151.178
151.562
ppm
0
20
40
60
80
100
120
140
160
180
200
220

HZK-III-253F

Sample Name:
HZK-III-253F
Archive directory:

Sample directory:

FidFile: HZK-III-253F

Pulse Sequence: Proton (s2pul)
Solvent: cdcl3
Data collected on: Jun 23 2011

Temp. 25.0 C / 298.1 K
Operator: jsk
INOVA-500 "nmr16"

Relax. delay 10.000 sec
Pulse 45.0 degrees
Acq. time 3.000 sec
Width 7996.0 Hz
8 repetitions
OBSERVE H1, 499.7720264 MHz
DATA PROCESSING
Resol. enhancement -0.0 Hz
FT size 65536
Total time 7 min 22 sec

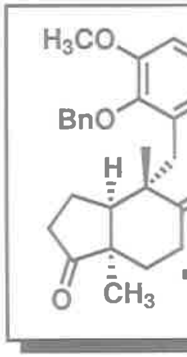

18

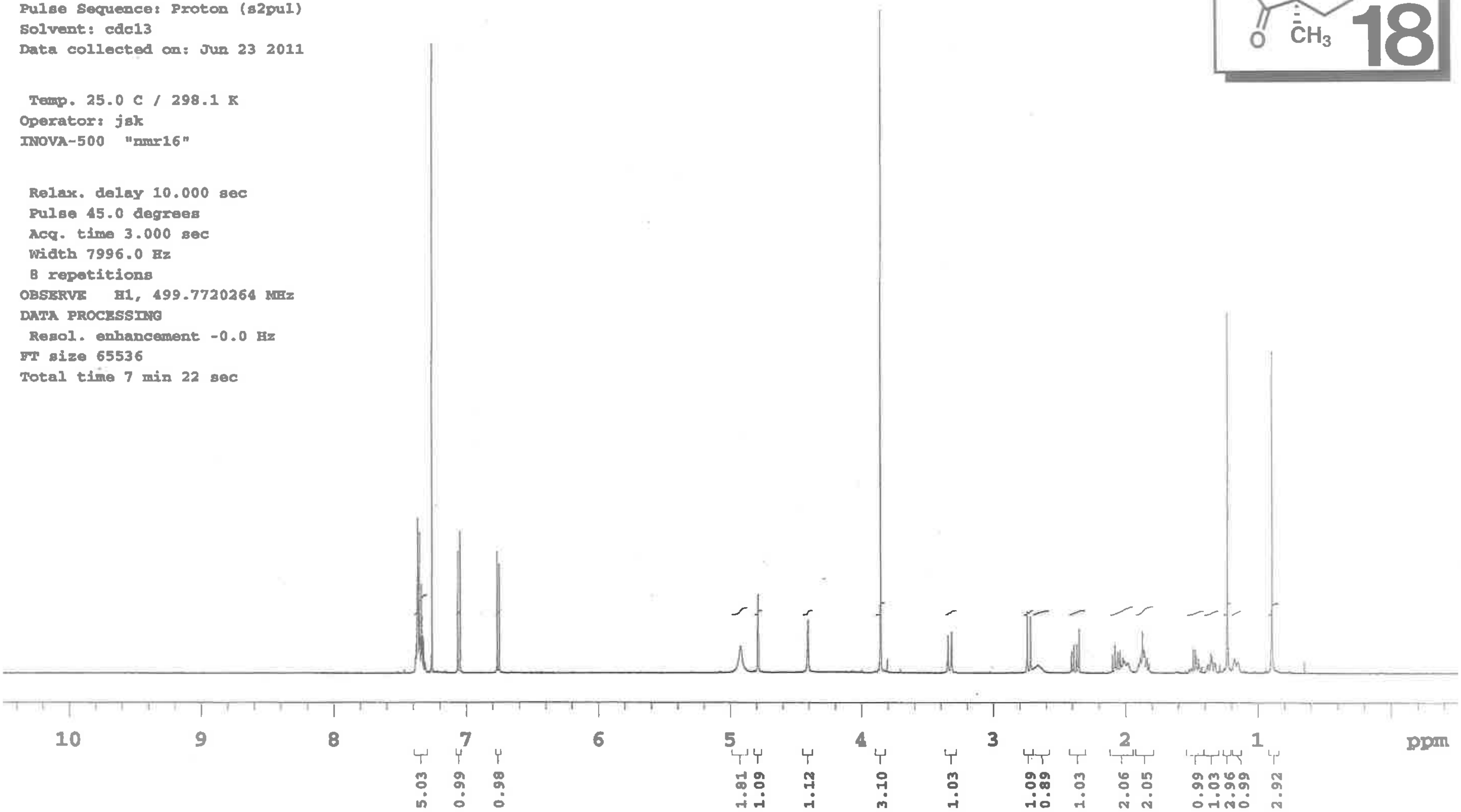

HZK-III-253F-carbon

Sample Name:
HZK-III-253F-carbon
Archive directory:

Sample directory:

FidFile: HZK-III-253F-carbon

Pulse Sequence: Carbon (s2pul)
Solvent: cdcl3
Data collected on: Jun 23 2011

Temp. 25.0 C / 298.1 K
Operator: jsk
INOVA-500 "nmr16"

Relax. delay 1.000 sec
Pulse 45.0 degrees
Acq. time 1.300 sec
Width 30165.9 Hz
346 repetitions
OBSERVE C13, 125.6677609 MHz
DECOUPLE H1, 499.7745112 MHz
Power 45 dB
continuously on
WALTZ-16 modulated
DATA PROCESSING
Line broadening 0.5 Hz
FT size 131072
Total time 19 min

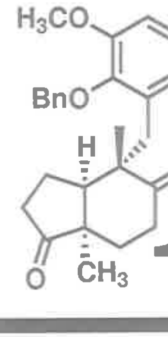

18

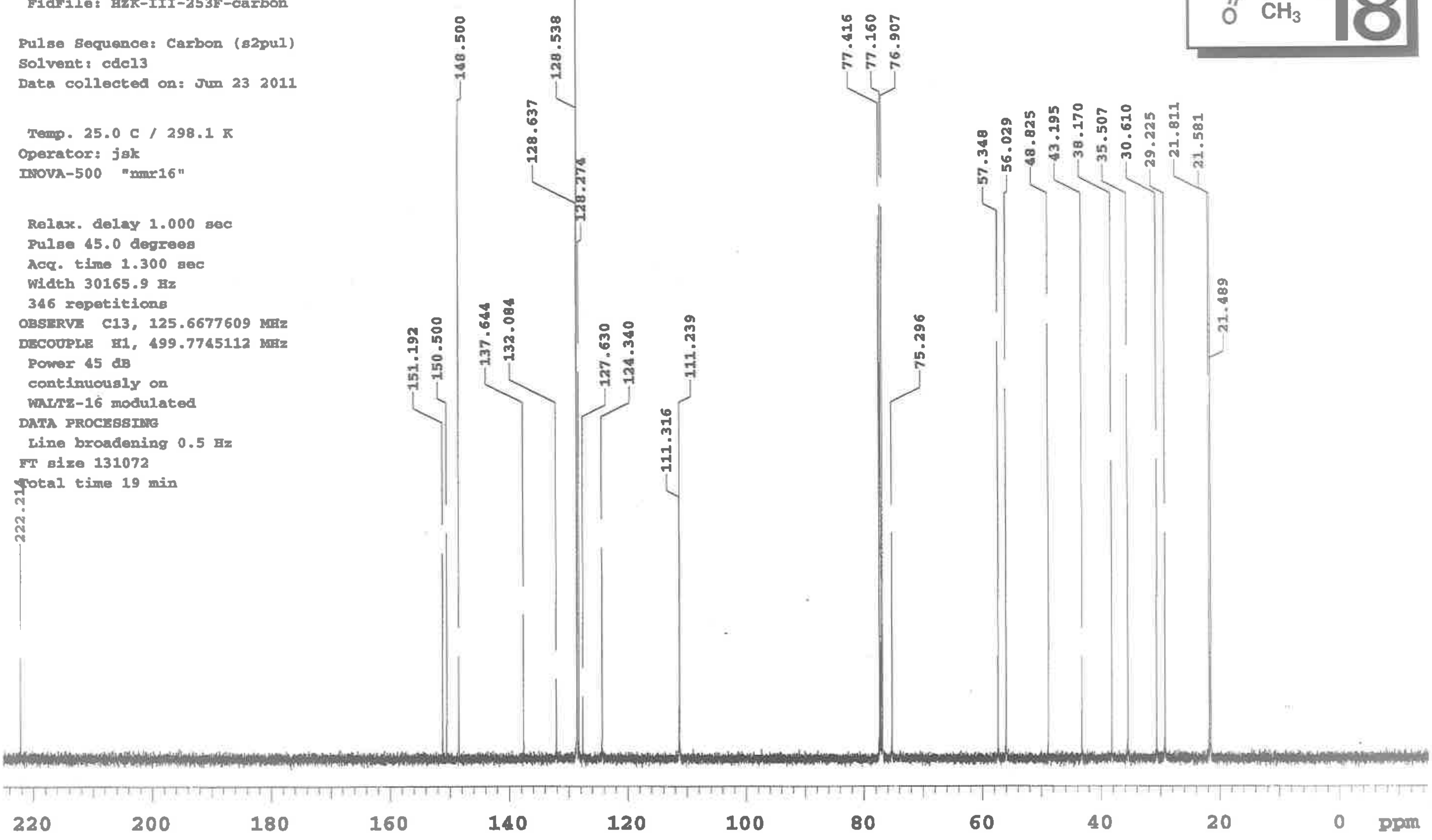

Sample Name:
HZK-III-250F
Archive directory:

Sample directory:

FidFile: HZK-III-250F

Pulse Sequence: Proton (s2pul)
Solvent: cdcl3
Data collected on: Jun 21 2011

Temp. 25.0 C / 298.1 K
Operator: jsk
INOVA-500 "nmr16"

Relax. delay 10.000 sec
Pulse 45.0 degrees
Acq. time 3.000 sec
Width 7996.0 Hz
3 repetitions
OBSERVE H1, 499.7720276 MHz
DATA PROCESSING
Resol. enhancement -0.0 Hz
FT size 65536
Total time 2 min 10 sec

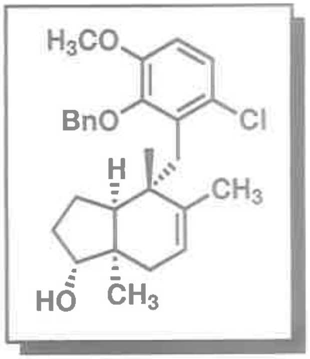

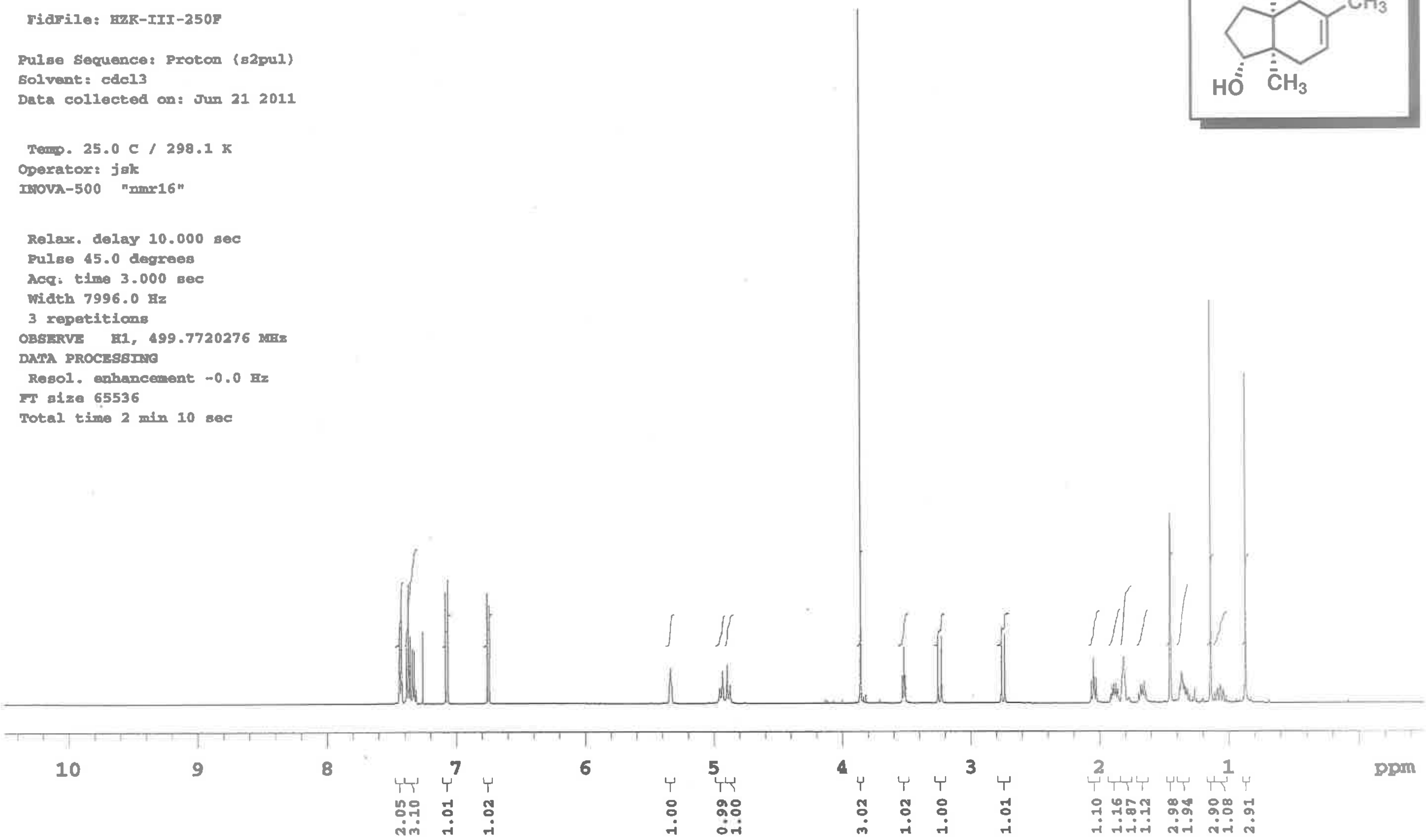

Sample Name:
HZK-III-250F-carbon
Archive directory:

Sample directory:

FidFile: HZK-III-250F-carbon

Pulse Sequence: Carbon (s2pul)
Solvent: cdcl3
Data collected on: Jun 21 2011

Temp. 25.0 C / 298.1 K
Operator: jsk
INOVA-500 "nmr16"

Relax. delay 1.000 sec
Pulse 45.0 degrees
Acq. time 1.300 sec
Width 30165.9 Hz
92 repetitions
OBSERVE C13, 125.6677604 MHz
DECOUPLE H1, 499.7745112 MHz
Power 45 dB
continuously on
WALTZ-16 modulated
DATA PROCESSING
Line broadening 0.5 Hz
FT size 131072
Total time 9 min 49 sec

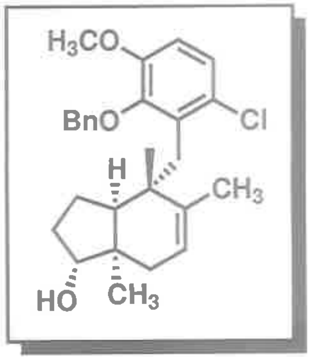

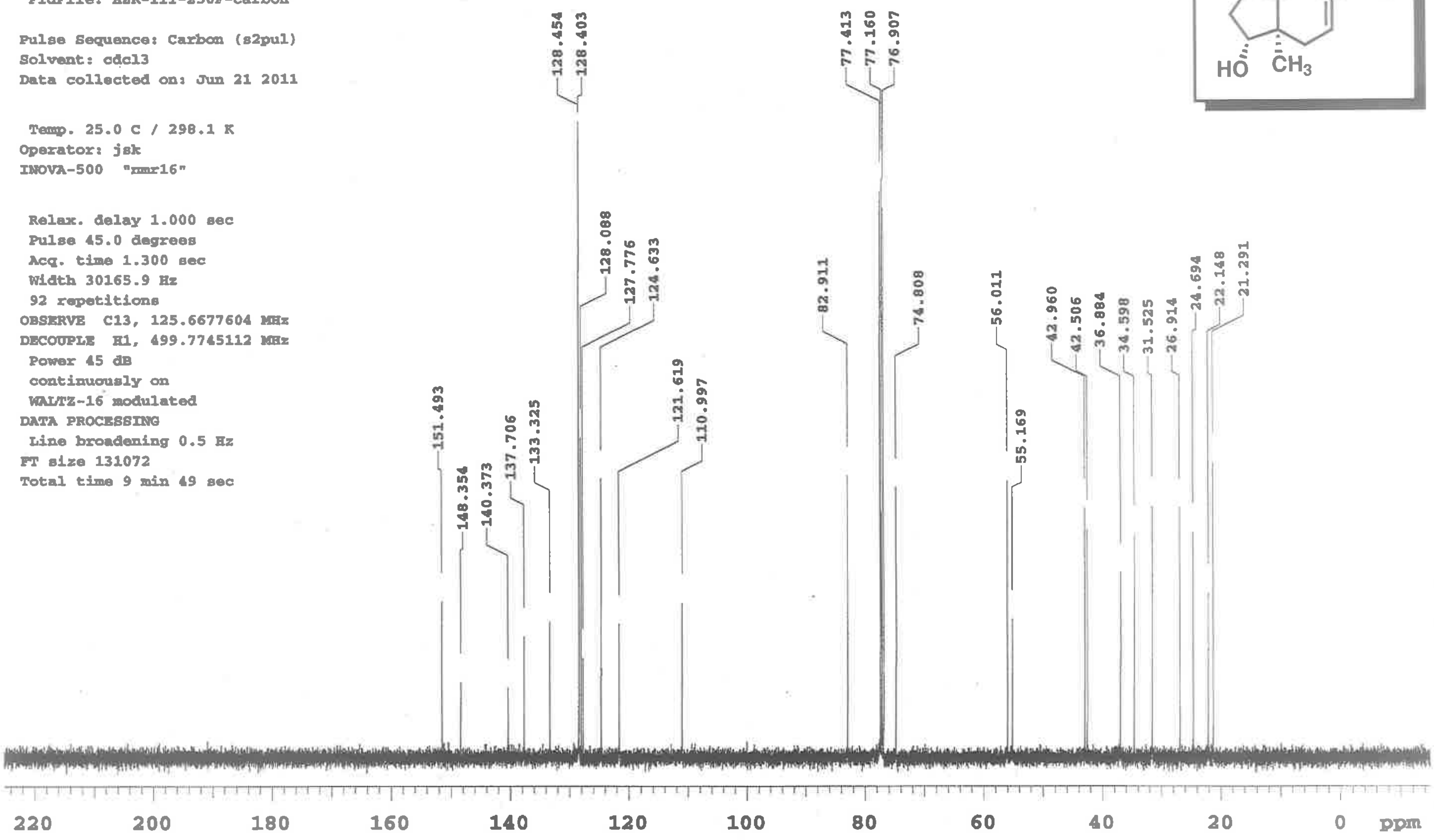

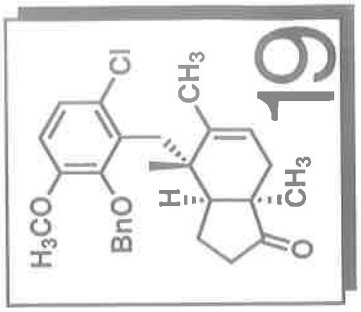

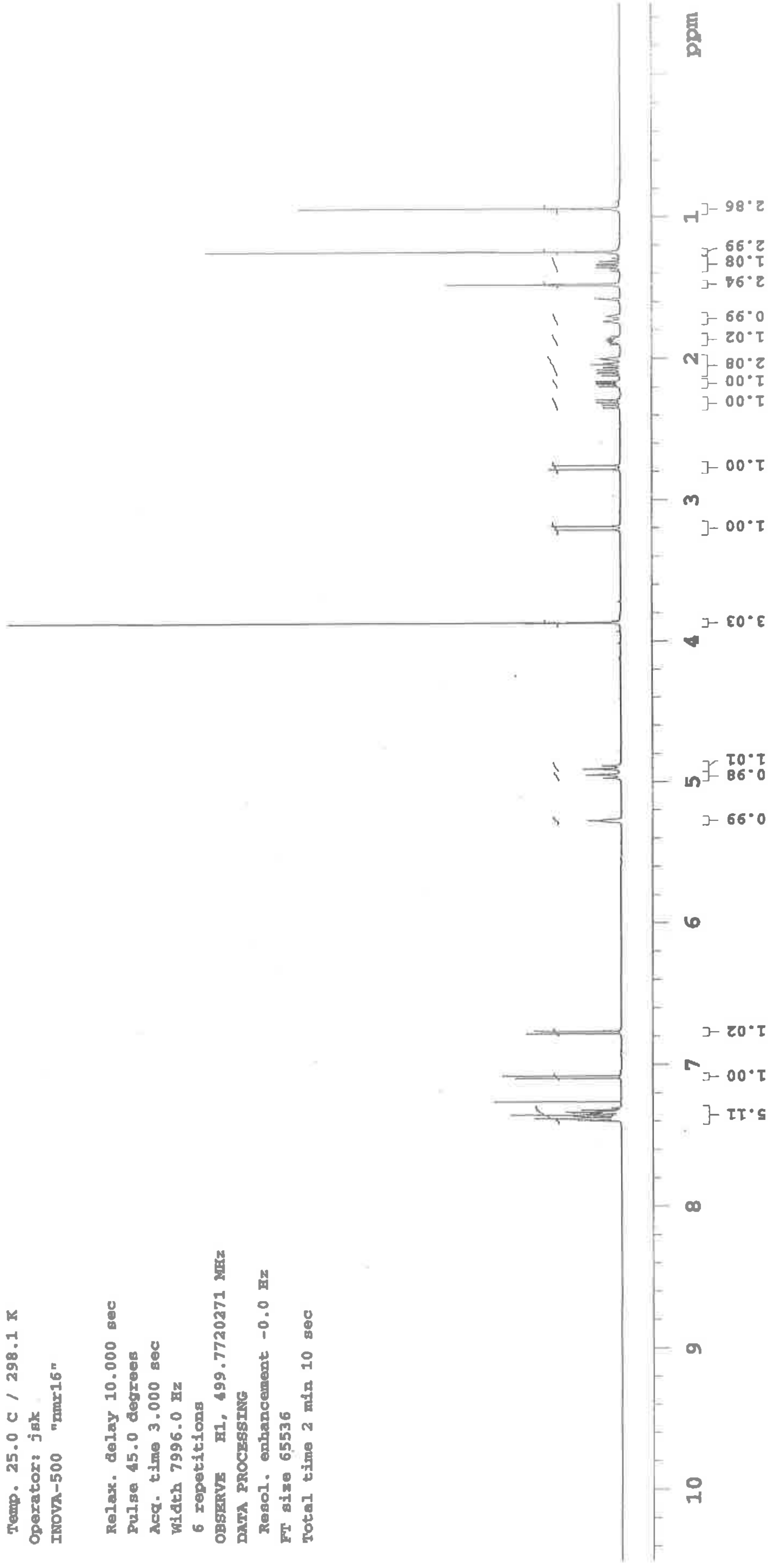

Sample Name:
VR-III-281f
Archive directory:

Sample directory:

FidFile: VR-III-281f

Pulse Sequence: Proton (s2pul)
Solvent: cdcl3
Data collected on: Jun 22 2011

Temp. 25.0 C / 298.1 K
Operator: jsk
INOVA-500 "nmr16"

Relax. delay 10.000 sec
Pulse 45.0 degrees
Acq. time 3.000 sec
Width 7996.0 Hz
6 repetitions
OBSERVE H1, 499.7720271 MHz
DATA PROCESSING
Resol. enhancement -0.0 Hz
FT size 65536
Total time 2 min 10 sec

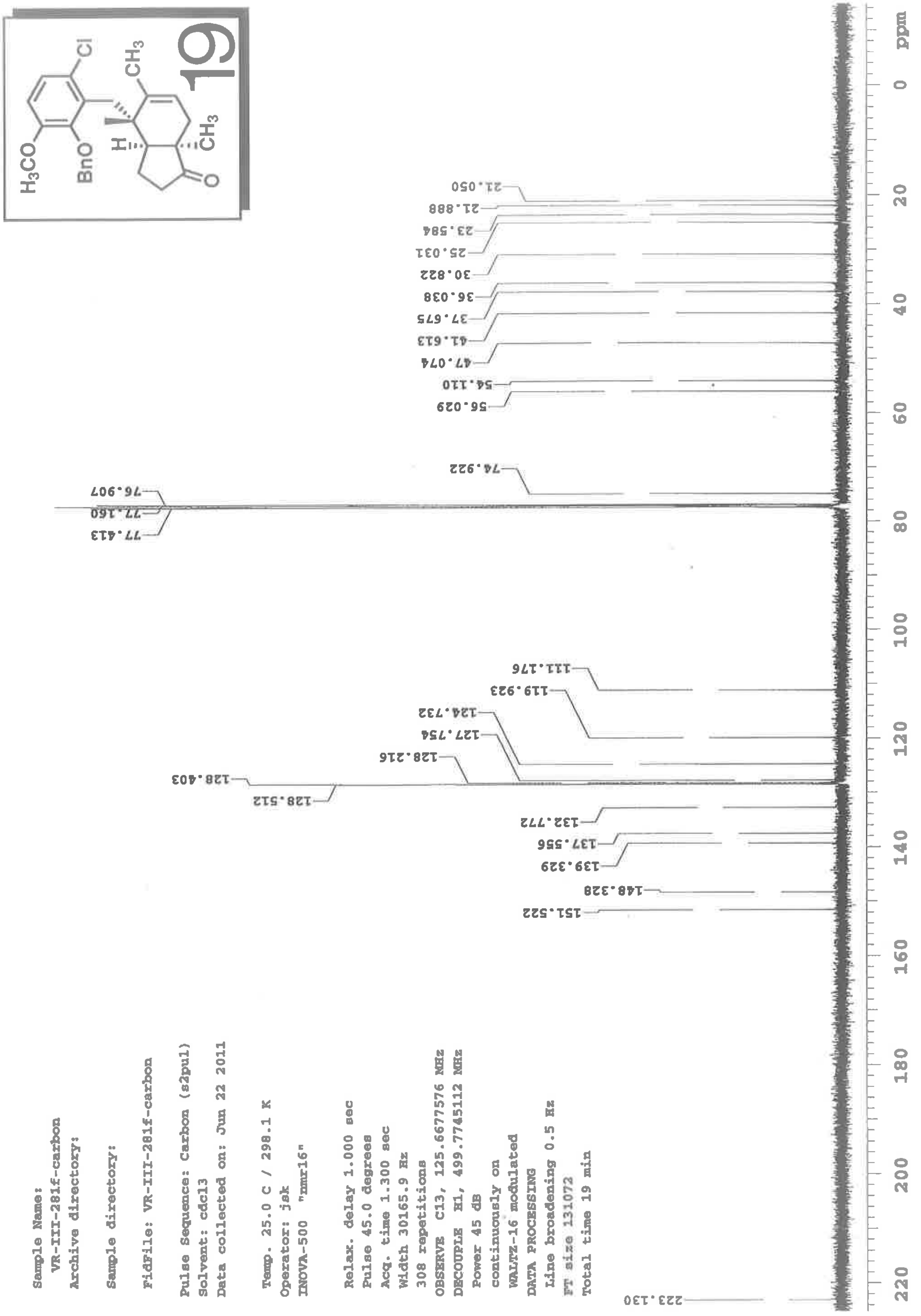
19
H3CO
BnO
Cl
CH3
CH3
H
Sample Name:
VR-III-281f-carbon
Archive directory:
Sample directory:
FidFile: VR-III-281f-carbon
Pulse Sequence: Carbon (s2pul)
Solvent: cdcl3
Data collected on: Jun 22 2011
Temp. 25.0 C / 298.1 K
Operator: jsk
INOVA-500 "nmr16"
Relax. delay 1.000 sec
Pulse 45.0 degrees
Acq. time 1.300 sec
Width 30165.9 Hz
308 repetitions
OBSERVE C13, 125.6677576 MHz
DECOUPLE H1, 499.7745112 MHz
Power 45 dB
continuously on
WALTZ-16 modulated
DATA PROCESSING
Line broadening 0.5 Hz
FT size 131072
Total time 19 min
223.130
151.522
148.328
139.329
137.556
132.772
128.512
128.403
128.216
127.754
124.732
119.923
111.176
77.413
77.160
76.907
74.922
56.029
54.110
47.074
41.613
37.675
36.038
30.822
25.031
23.584
21.888
21.050
ppm
220
200
180
160
140
120
100
80
60
40
20
0

Sample Name:
VR-III-283-2-fa
Archive directory:

Sample directory:

FidFile: VR-III-283-2-fa

Pulse Sequence: Proton (s2pul)
Solvent: cdcl3
Data collected on: Jul 10 2011

Operator: jsk
INOVA-500 "nmr16"

Relax. delay 10.000 sec
Pulse 45.0 degrees
Acq. time 3.000 sec
Width 7996.0 Hz
13 repetitions
OBSERVE H1, 499.7720269 MHz
DATA PROCESSING
Resol. enhancement -0.0 Hz
FT size 65536
Total time 3 min 54 sec

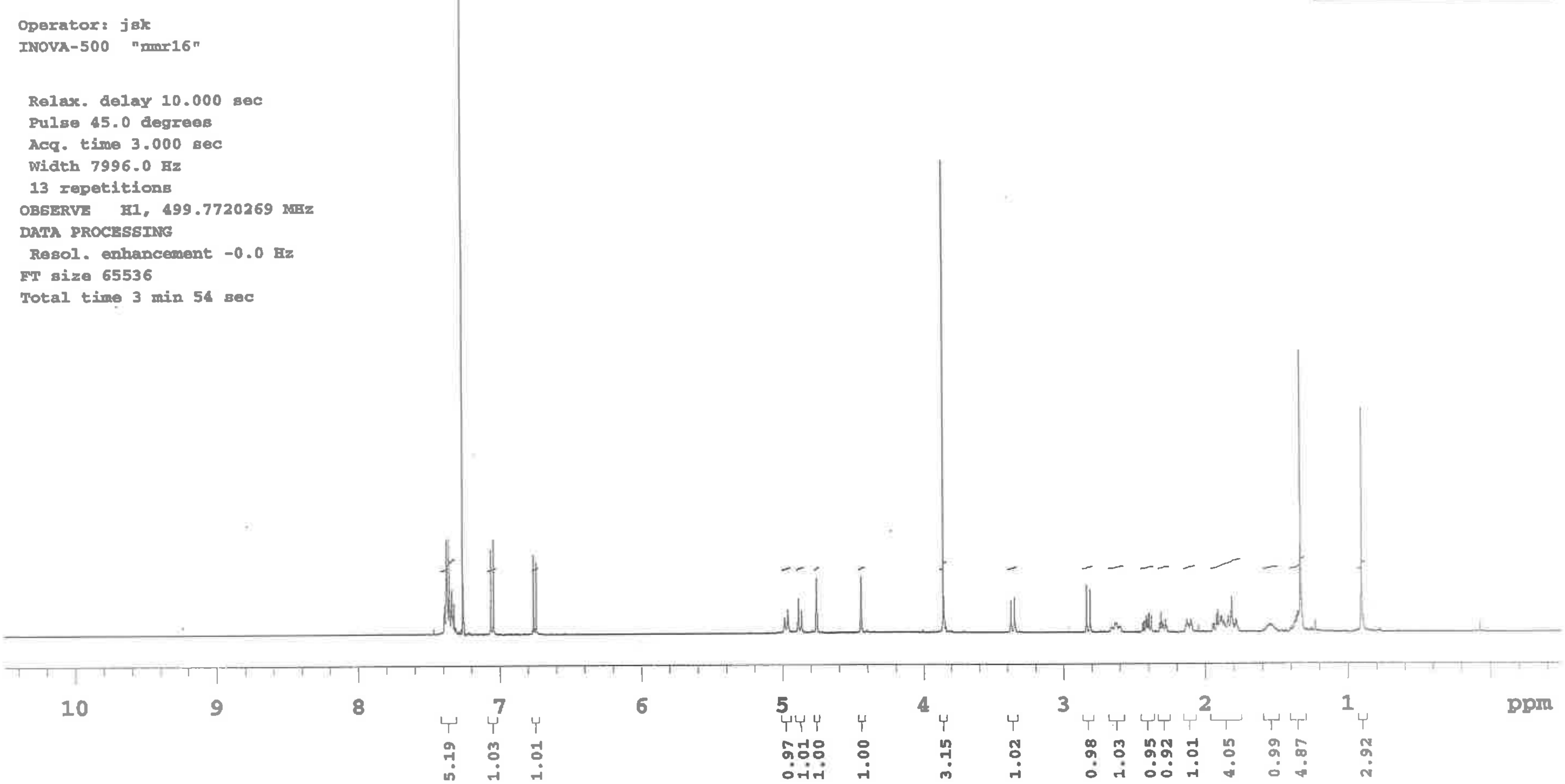

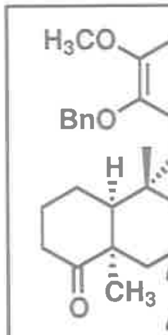

20

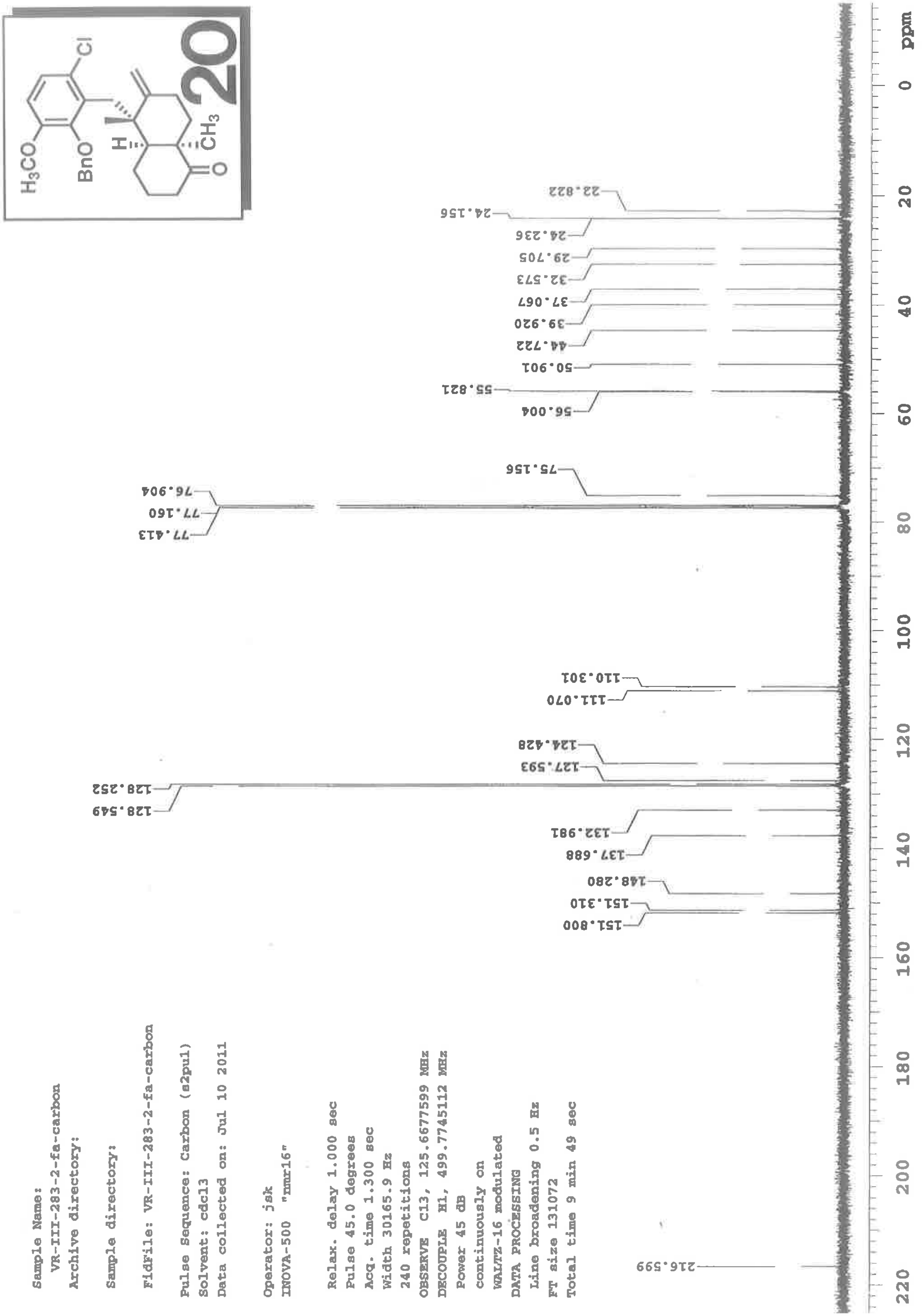

H_3CO
BnO
Cl
H
CH_3
O
20
Sample Name:
VR-III-283-2-fa-carbon
Archive directory:
Sample directory:
FidFile: VR-III-283-2-fa-carbon
Pulse Sequence: Carbon (s2pul)
Solvent: cdcl3
Data collected on: Jul 10 2011
Operator: jsk
INOVA-500 "nmr16"
Relax. delay 1.000 sec
Pulse 45.0 degrees
Acq. time 1.300 sec
Width 30165.9 Hz
240 repetitions
OBSERVE C13, 125.6677599 MHz
DECOUPLE H1, 499.7745112 MHz
Power 45 dB
continuously on
WALTZ-16 modulated
DATA PROCESSING
Line broadening 0.5 Hz
FT size 131072
Total time 9 min 49 sec
22.822
24.156
24.236
29.705
32.573
37.067
39.920
44.722
50.901
55.821
56.004
75.156
76.904
77.160
77.413
110.301
111.070
124.428
127.593
128.252
128.549
132.981
137.688
148.280
151.310
151.800
216.599
ppm
0
20
40
60
80
100
120
140
160
180
200
220

Sample Name:
VR-III-283fb
Archive directory:

Sample directory:

FidFile: VR-III-283fb

Pulse Sequence: Proton (s2pul)
Solvent: cdcl3
Data collected on: Jul 8 2011

Operator: jsk
INOVA-500 "nmr16"

Relax. delay 10.000 sec
Pulse 45.0 degrees
Acq. time 3.000 sec
Width 7996.0 Hz
16 repetitions
OBSERVE H1, 499.7720266 MHz
DATA PROCESSING
Resol. enhancement -0.0 Hz
FT size 65536
Total time 3 min 54 sec

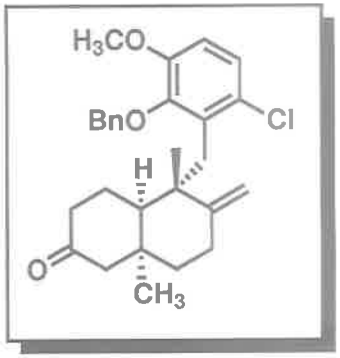

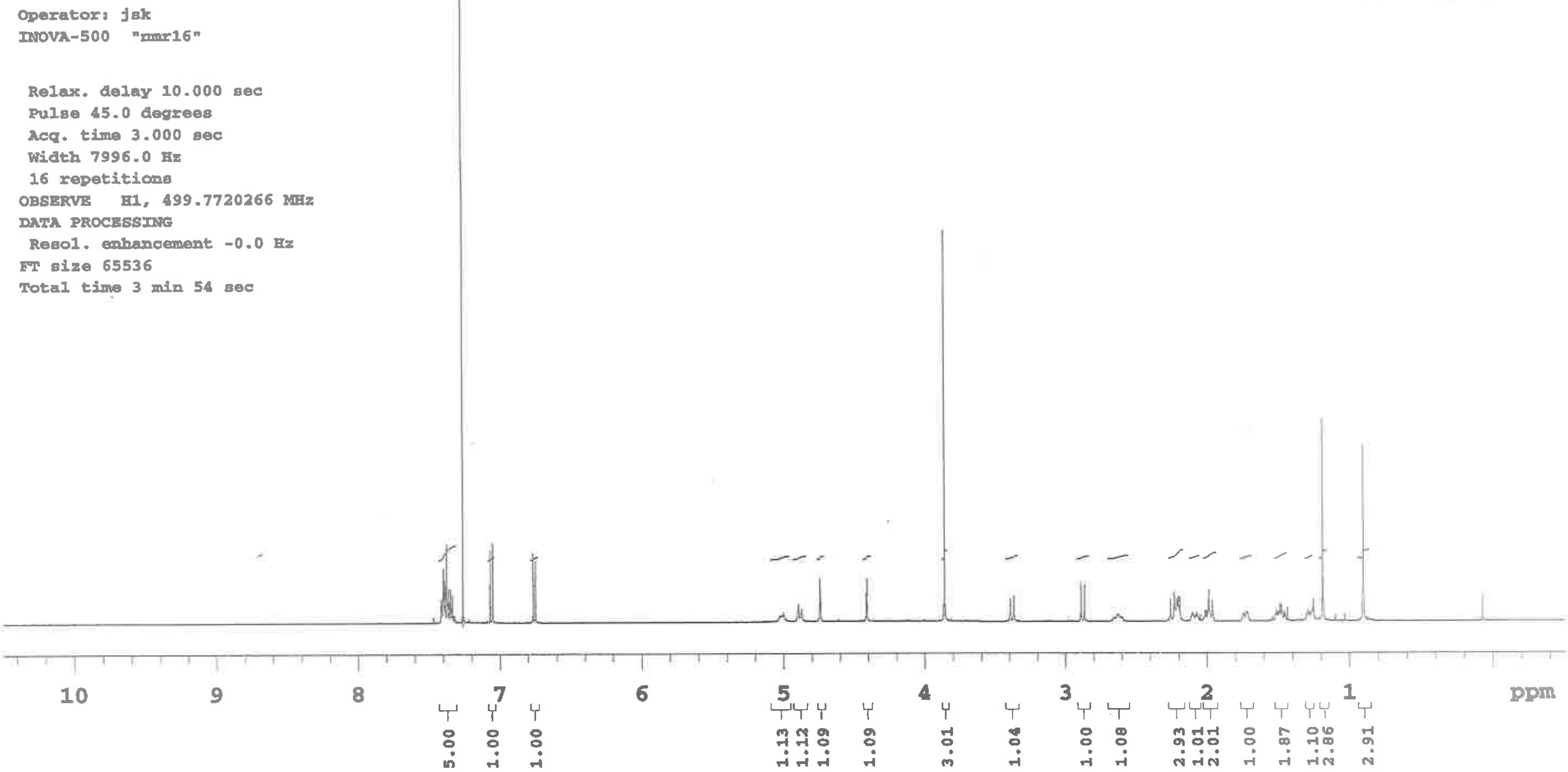

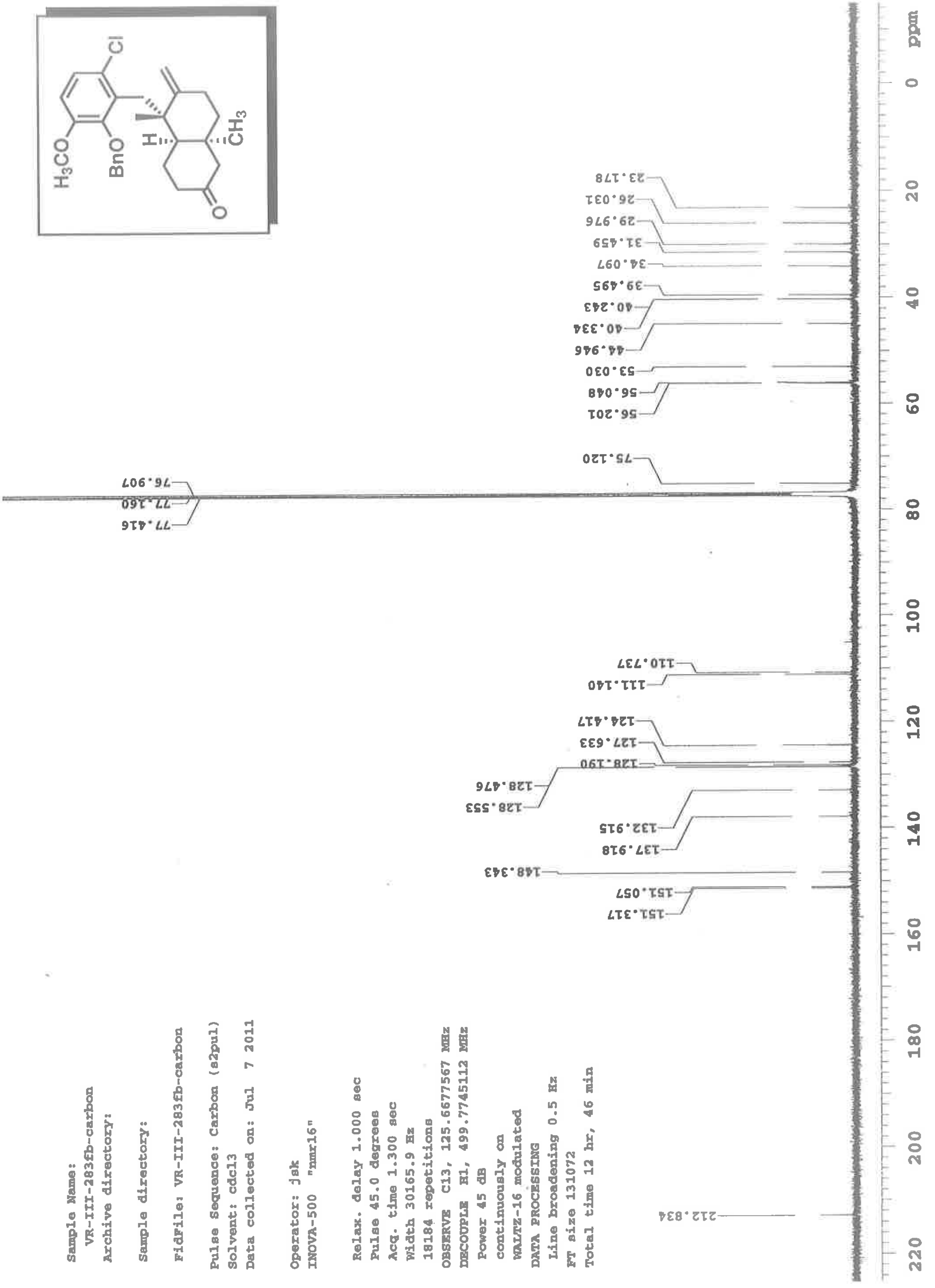
H3CO
BnO
Cl
H
CH3
O
Sample Name:
VR-III-283fb-carbon
Archive directory:
Sample directory:
FidFile: VR-III-283fb-carbon
Pulse Sequence: Carbon (s2pul)
Solvent: cdcl3
Data collected on: Jul 7 2011
Operator: jsk
INOVA-500 "nmr16"
Relax. delay 1.000 sec
Pulse 45.0 degrees
Acq. time 1.300 sec
Width 30165.9 Hz
18184 repetitions
OBSERVE C13, 125.6677567 MHz
DECOUPLE H1, 499.7745112 MHz
Power 45 dB
continuously on
WALTZ-16 modulated
DATA PROCESSING
Line broadening 0.5 Hz
FT size 131072
Total time 12 hr, 46 min
212.834
151.317
151.057
148.343
137.918
132.915
128.553
128.476
128.190
127.633
124.417
111.140
110.737
77.416
77.160
76.907
75.120
56.201
56.048
53.030
44.946
40.334
40.243
39.495
34.097
31.459
29.976
26.031
23.178
220
200
180
160
140
120
100
80
60
40
20
0
ppm

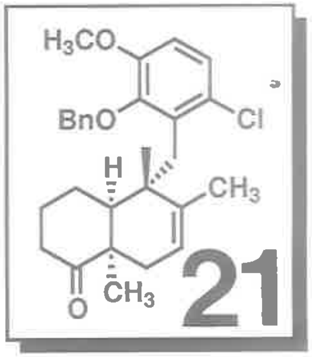

21

Sample Name:
VR-III-282f
Archive directory:

Sample directory:

FidFile: VR-III-282f

Pulse Sequence: Proton (s2pul)
Solvent: cdcl3
Data collected on: Jul 6 2011

Temp. 25.0 C / 298.1 K
Operator: jsk
INOVA-500 "nmr16"

Relax. delay 10.000 sec
Pulse 45.0 degrees
Acq. time 3.000 sec
Width 7996.0 Hz
12 repetitions
OBSERVE H1, 499.7720266 MHz
DATA PROCESSING
Resol. enhancement -0.0 Hz
FT size 65536
Total time 3 min 54 sec

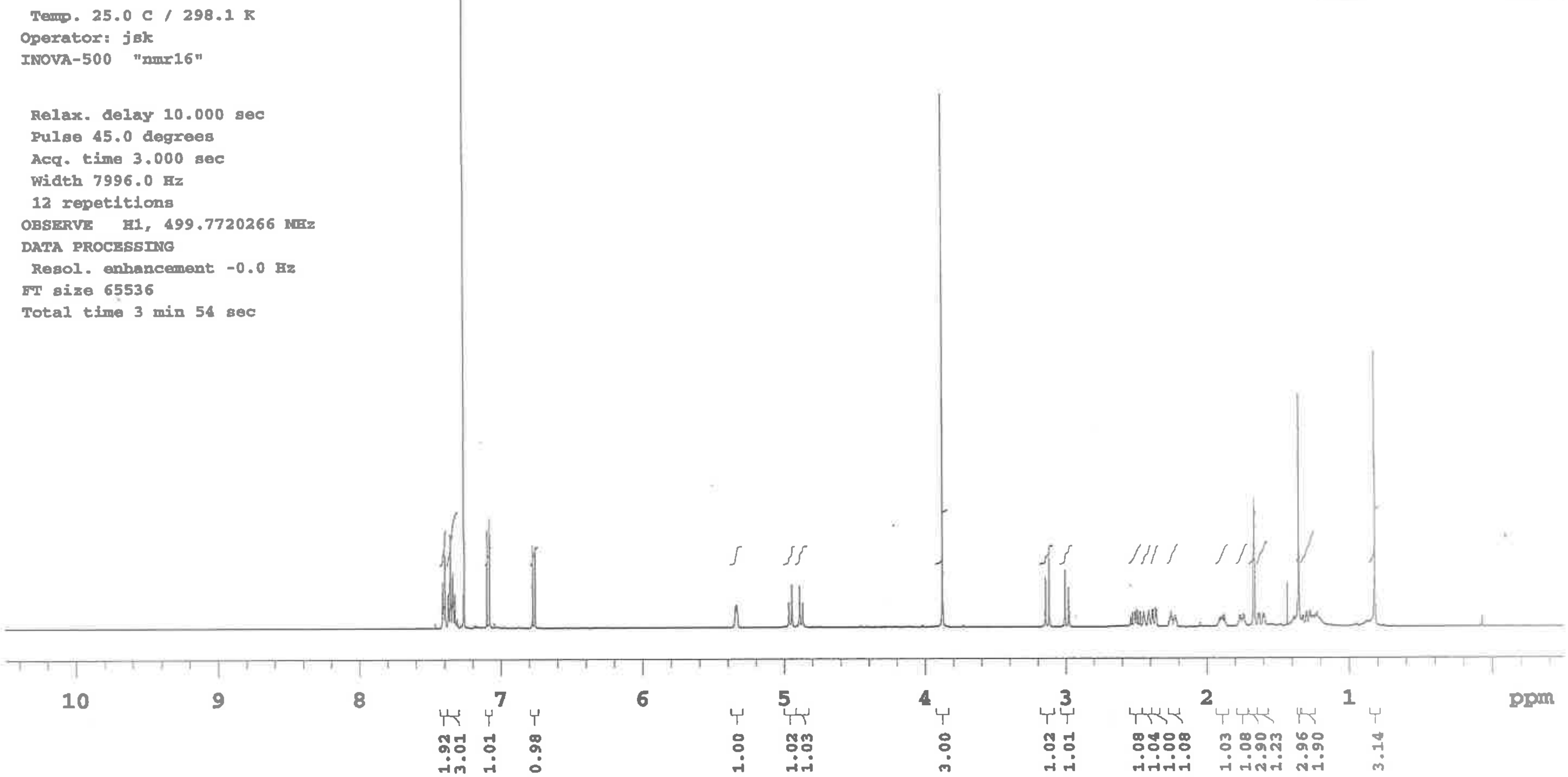

Sample Name:
VR-III-282f-carbon
Archive directory:

Sample directory:

FidFile: VR-III-282f-carbon

Pulse Sequence: Carbon (s2pul)
Solvent: cdcl3
Data collected on: Jul 6 2011

Temp. 25.0 C / 298.1 K
Operator: jsk
INOVA-500 "nmr16"

Relax. delay 1.000 sec
Pulse 45.0 degrees
Acq. time 1.300 sec
Width 30165.9 Hz
204 repetitions
OBSERVE C13, 125.6677609 MHz
DECOUPLE H1, 499.7745112 MHz
Power 45 dB
continuously on
WALTZ-16 modulated
DATA PROCESSING
Line broadening 0.5 Hz
FT size 131072
Total time 19 min

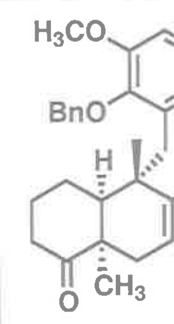

21

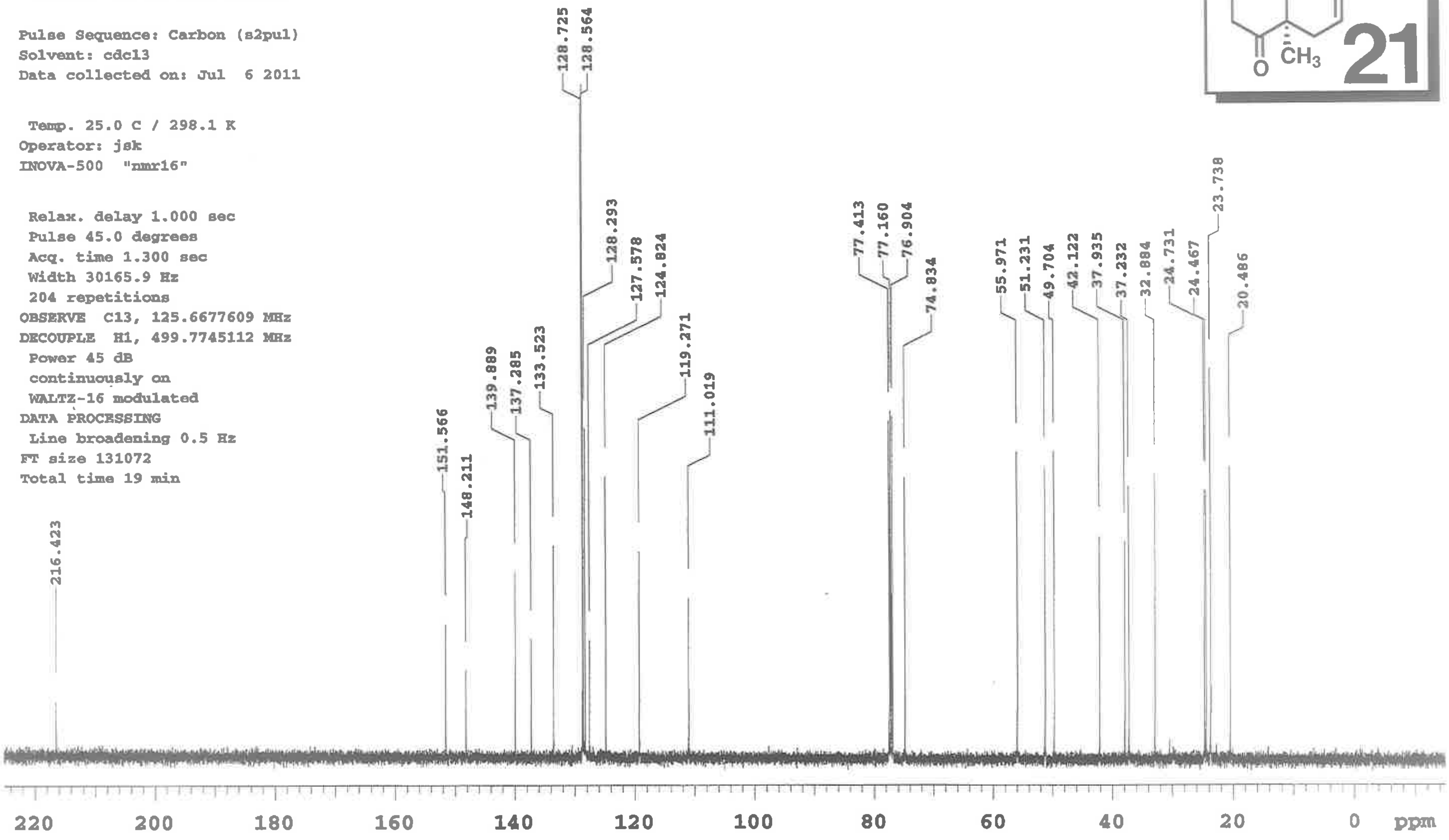

Supplement: Supplementary file 1 [file molecules-22-01041-s001.pdf]
